# A Human Trafficking Educational Program and Point-of-Care Reference Tool for Pediatric Residents

**DOI:** 10.15766/mep_2374-8265.11179

**Published:** 2021-09-13

**Authors:** Anjali Garg, Preeti Panda, Sindhoosha Malay, Jerri A. Rose

**Affiliations:** 1 Resident Physician, Department of Pediatrics, Rainbow Babies and Children's Hospital; 2 Statistician, Case Western Reserve University School of Medicine; 3 Associate Professor, Department of Pediatrics, Rainbow Babies and Children's Hospital and Case Western Reserve University School of Medicine

**Keywords:** Human Trafficking, Sex Trafficking, Pediatrics, Commercial Sexual Exploitation of Children, Advocacy, Trauma-Informed Care, Child Abuse, Adolescent Medicine, Case-Based Learning

## Abstract

**Introduction:**

Trafficked youth experience complex health issues such as substance abuse, severe physical trauma, and sexual health problems, with many entering trafficking between 15 and 17 years old. There is increasing awareness of the need to educate pediatric health care providers on identifying and aiding trafficked children; however, critical gaps in the literature exist regarding educational sessions specific to the human trafficking of pediatric patients. Our objective was to implement and evaluate a survivor-informed educational session for pediatric resident physicians to improve identification of and assistance to trafficked youth in the clinical setting.

**Methods:**

We designed an educational session on human trafficking, which included a 60-minute interactive didactic presentation and distribution of a point-of-care reference tool, in collaboration with a survivor of human trafficking, for 59 pediatric trainees in 2019. We utilized pre/post knowledge assessments. Data analysis included descriptive statistics and Fisher exact analysis.

**Results:**

Of 99 total eligible residents, 59 (59%) participated. Statistically significant increases in correct identification of hypothetical trafficked youth and next steps for intervention were observed. Over 80% of participants reported comfort with defining, recognizing, referring to, and understanding health consequences of human trafficking on the postassessment, compared to 25% on the preassessment (*p* < .001).

**Discussion:**

Our educational session resulted in statistically significant increased comfort in identification of human trafficking victims and can be replicated at other institutions. The point-of-care reference tool—which can be adapted for use in different settings—can guide pediatric residents in managing suspected trafficked youth in the clinical setting.

## Educational Objectives

By the end of this educational session, learners will be able to:

1. Define human trafficking.
2. Describe health consequences of human trafficking.
3. Screen and intervene for human trafficking victims in the clinical setting.
4. Define trauma-informed care and its utilization with the trafficked population.
5. Report increased comfort levels around the topic of, identification of, and intervention for trafficked youth.

## Introduction

Human trafficking is the use of force, fraud, or coercion with adults and minors to perform sex or labor acts, and it affects domestic citizens as prevalently as international subjects.^1–3^ More than 80% of survivors have had contact with the health care system during the course of their exploitation, putting health care providers in a unique position to be able to intervene.^1,2,4–6^ Many individuals enter their trafficking situation while still a minor.^7^ Globally, over 4 million children are estimated to be trafficked, comprising 20% of the forced labor burden.^6^ In the US, over 2,500 of 11,500 cases identified by the National Human Trafficking Hotline in 2019 were children.^8^ It is therefore imperative that pediatric trainees are educated and well prepared to recognize and appropriately intercede for these children and adolescents.^4^

Health problems experienced by trafficked youth as a result of their exploitation have been well described in the literature.^2,4,5^ The threats, persistent stress, and repeated abuse they experience adversely affect mental health and may contribute to the development of conditions including post-traumatic stress disorder, depression, and anxiety.^9^ Survivors of trafficking often experience malnutrition due to inadequate dietary intake and poor hygiene given their living circumstances.^6^ Many suffer from substance abuse, physical injuries, and sexual health problems.^6^ Early recognition of and intervention for trafficked children are imperative to prevent these serious consequences.^2,3^

Trafficked children are cared for throughout the health care system: in emergency departments, primary care offices, urgent care centers, community health clinics, reproductive health clinics, and hospital inpatient units.^10^ Therefore, educating physicians across a variety of practice settings is essential.^1,2,4,5^ The few existing previous studies on human trafficking education have demonstrated an increase in victim intervention and have documented increased provider sensitization to signs of victimization.^4^ However, the existing literature and available online training modules are focused on adult trafficking, and studies have largely targeted emergency medicine providers.^11,12^ To date, a survivor-informed human trafficking training for pediatric trainees has not been comprehensively evaluated.^2,4,12^ Additionally, there are no existing *MedEdPORTAL* publications focusing on child trafficking.

We designed and implemented this module to create a survivor-informed educational program and point-of-care reference tool for pediatric trainees to improve identification of and assistance to trafficked youth. We fill a critical gap in medical education literature by providing training specific to the trafficking of pediatric patients and a model for implementing survivor guidance that can be replicated at other institutions.

## Methods

### Setting and Participants

The educational program team consisted of two pediatric resident physicians passionate about antitrafficking advocacy, a sexual assault nurse examiner who was a local antitrafficking expert, a survivor leader, and an attending physician for guidance and oversight. The survivor leader worked with an existing leadership development program for survivors of human trafficking and had previous experience speaking to health care professionals. The guiding principle of the team was to introduce trainees to the topic of human trafficking of children, explain its relevance to pediatrics, and teach ways to appropriately and safely intervene to aid trafficked youth.

Our team designed and implemented an educational session focused on child trafficking within a pediatric residency program at an urban tertiary children's hospital in Cleveland, Ohio, from February to April 2019. All categorical pediatric, medicine-pediatrics, genetics-pediatrics, and neurology-pediatrics residents training at the institution during the time period were eligible for participation. We offered sessions during prescheduled didactic times held by the residency program.

### The Didactic Session

The educational program included (1) a live, interactive, didactic session focused on recognition and management of trafficked children and (2) implementation of a novel point-of-care reference tool that was provided to participants for quick reference and guidance in the clinical setting. Main goals of our session were to teach learners to define human trafficking, describe health consequences of trafficked youth, and screen and intervene on behalf of trafficking victims in the clinical setting. We also covered the concept of trauma-informed care as it applied to this patient population. We aimed to increase comfort around the topic of human trafficking so that trainees could better identify and aid trafficked youth. The included preceptor guide provides details regarding implementation of the education session (Appendix A).

The didactic session consisted of a 60-minute interactive PowerPoint presentation (Appendix B). Our learning objectives and session content were informed by a review of the current literature on human trafficking, including an adult-based online training module, the local survivor's personal experiences and perspectives, local statistics as they pertained to human trafficking, and the experiences of the instructors with treating child survivors of human trafficking.^9^ The instructors developed educational content in collaboration with a local human trafficking expert leader, who was appropriately compensated as a professional consultant for their time and efforts. We verified that the educational content met existing evidence-based standards for comprehensive training through the Health Care Provider Human Trafficking Training: Assessment Tool.^10^ Two instructors and the local survivor led the didactic sessions.

The second component of the educational session involved distribution of a novel point-of-care reference card that we created to support long-term retention of health care provider knowledge regarding human trafficking identification and intervention in the clinical setting (Appendices C and D). Lists of trafficking red flags, screening questions, and contact information for key human trafficking support resources were included on the card. The cost of the laminated badge cards we distributed was $350 for 500 cards. We reviewed and distributed the point-of-care algorithm card and postsession assessment (Appendices E and F) at the end of the session. Postsession assessments were scored against our answer sheet as well as compared to presession assessments to determine which educational gaps were corrected.

The following time line was used for the education session:

• 5 minutes: completion of presession assessment.
• 5 minutes: welcome and introduction of presenters (including survivor if present).
• 60 minutes: interactive didactic session (guided by slides) and explanation of algorithm cards.
• 1-2 minutes: distribution of algorithm cards.
• 5 minutes: completion of postsession assessment.

### Effective Survivor Partnership

Responsibly including survivor leadership was important to the education team. Given a survivor's personal experience with human trafficking, avoiding any situations that would retraumatize them was critical to our team. The survivor leader provided feedback and guidance on inclusion of survivor- and trauma-sensitive language in our session, as well as on content validity. Additionally, the survivor leader was a key teacher during the didactic session. We defined roles and responsibilities clearly prior to the sessions for accountability. The survivor leader was compensated as a professional consultant for their time and contributions to the program.

### Educational Assessment Tools

We assessed the impact of the educational content through an anonymous assessment completed immediately before and after the didactic session to measure participants’ knowledge, skills, and attitudes regarding caring for trafficked children (Appendices E and F). We organized this assessment into three sections. Section one (questions 2A-3B) evaluated participants’ ability to identify trafficked children based on details provided in hypothetical case scenarios. We adapted scenarios from a previous study of provider knowledge about human trafficking to focus on pediatric patients.^2^ Section two (questions 4A-5) assessed participants’ knowledge shared during the educational session. Section three (questions 7A-7E) assessed participants’ comfort levels related to recognizing trafficked youth in clinical practice and implementing appropriate interventions. While all participants were present for the educational session and were given the point-of-care reference card, learner completion of the assessments was completely voluntary and occurred without the educators present.

Regarding the knowledge assessment results, we treated the pre- and postsession results as two independent groups. Fisher's exact two-tailed analysis was used to compare unpaired pre- and postsession responses. Participants received a score of 1 for each correct answer on multiple-choice and true-or-false items or a score of 0 for incorrect answers. The percentage of total correct answers for pre- and postsession results was compared. For items that utilized a 5-point Likert scale, we dichotomized responses into scores of 1–3 (1 = *very uncomfortable*/*likely*, 2 = *uncomfortable*/*unlikely*, 3 = *neutral*) and scores of 4–5 (4 = *comfortable*/*likely*, 5 = *very comfortable*/*likely*) for statistical comparison. We compared responses for participants reporting prior training related to human trafficking both separately and along with the whole group of participants using Fisher's exact two-tailed analysis.

### Institutional Review Board Statement

The University Hospitals Cleveland Medical Center Institutional Review Board reviewed and fully approved our educational session and assessments prior to implementation.

## Results

Of 99 total residents enrolled in participating residency programs, 59 (59%) participated in our sessions. We conducted four didactic sessions with roughly 15 participants each. In total, 27 PGY 1, 13 PGY 2, 16 PGY 3, and three PGY 4 participants took part in the didactic sessions, with 22 of those participants having previous human trafficking training.

All 59 (100%) participants completed the session preassessment, while 58 of 59 (98%) completed the postassessment. To maintain the anonymity of participants, postgraduate year was the only demographic information collected (Table 1). Of the 59 participating residents, 37% (22 of 59) reported they had previously completed training related to human trafficking, and 20% (12 of 59) had prior training on child trafficking.

**Table 1.**
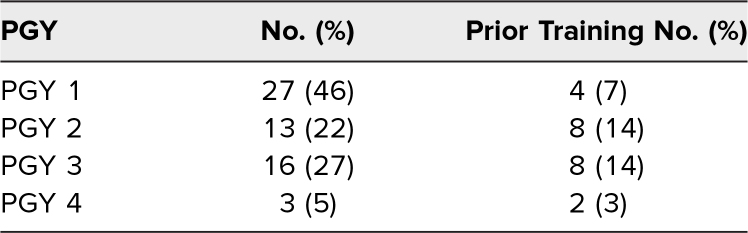
Resident Participant Demographics

Table 2 summarizes participants’ performance on knowledge assessment items before and after the educational session, while Table 3 summarizes performance related to the subgroup of participants with prior human trafficking training.

**Table 2.**
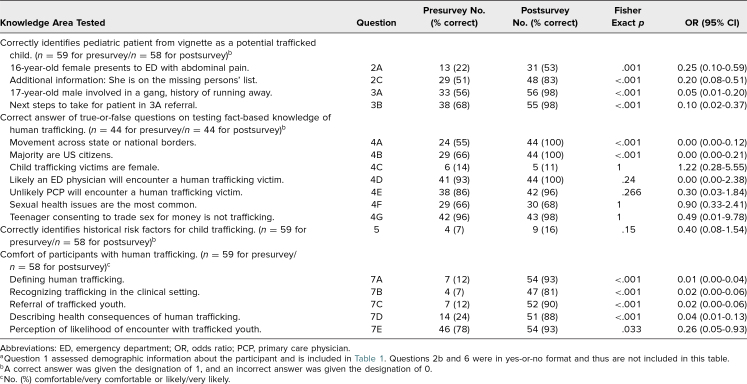
Resident Participant Performance on Assessment^a^ Before and After Educational Intervention

**Table 3.**
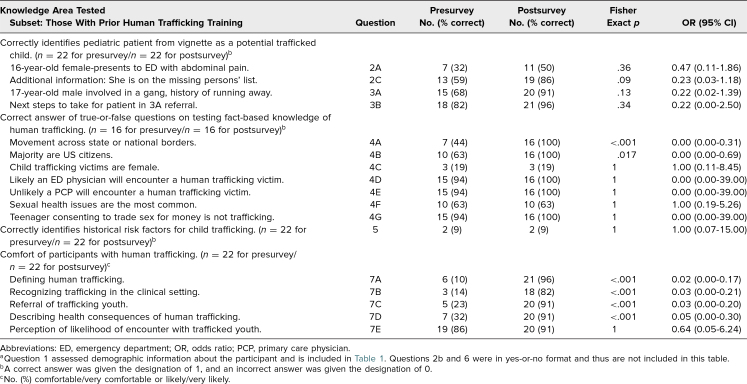
Resident Participant Performance on Assessment^a^ Before and After Educational Intervention for Those With Prior Training

### Knowledge Assessment Section 1: Identification of Trafficked Youth

This section was targeted at Educational Objective 3, which focused on training participants to screen and intervene for children in a trafficking situation. Overall, statistically significant increases in the correct identification of hypothetical trafficked youth and next steps for intervention were seen amongst the total resident cohort when comparing the pre- and postsession results. For example, when provided with a hypothetical case involving an adolescent female who was trafficked, 13 of 59 participants (22%) correctly identified her as a trafficked child prior to the education session, compared to 31 out of 58 (53%) on the postassessment (*p* = .001). When an additional scenario for identification of a male trafficked youth was presented, 33 of 59 participants (56%) correctly identified human trafficking as the likely diagnosis for the patient on the preassessment, compared to 56 of 58 (98%) on the postassessment (*p* < .001), during which participants also correctly identified next steps for intervention (*p* < .001).

### Knowledge Assessment Section 2: Assessing Knowledge Retention

The second section targeted Educational Objectives 1 and 2, which focused on defining human trafficking and the health consequences of trafficked youth. Of 59 participants, 44 (75%) provided answers to items in the second section of the knowledge assessment on both the pre- and postassessments. Questions regarding citizenship and necessity to cross borders (questions 4A and 4B) showed statistically significant increases in correct answers following the session, with an increase from 24 correct responses in the preassessment to 44 correct responses in the postassessment for question 4A and from 29 to 44 for question 4B (*p* < .001). Amongst residents with prior training, only one question (4A) had a statistically significant increase (*p* < .001). Several questions (4D, 4E, and 4G) received a high percentage of correct answers both before and after the session, and therefore, no statistically significant changes were observed.

### Knowledge Assessment Section 3: Self-Reported Attitudes Related to Caring for Trafficked Children

This last section targeted Educational Objective 5, which focused on increasing comfort around the topic of, identification of, and intervention for trafficked youth. Self-reported comfort levels related to various aspects of caring for trafficked children increased significantly following the session (Figure). Over 80% of participants reported they were comfortable or very comfortable with defining, recognizing, referring to, and understanding health consequences of human trafficking on the postassessment (questions 7A-7D), while less than 25% of participants reported these same comfort levels on the preassessment (*p* < .001). These same items also showed statistically significant increases in comfort amongst participants reporting previous human trafficking training (*p* < .001).

**Figure.**
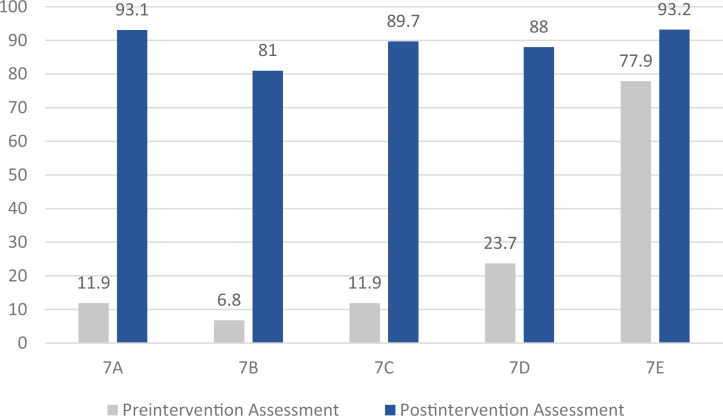
Self-reported comfort levels related to various aspects of caring for trafficked children increased significantly following the intervention. Numbers represent percentages of participants in pre- (*n* = 59) and postintervention (*n* = 58) assessments. For comparisons 7A–7D, *p* < .001, and for 7E, *p* = .033. The comfort levels reported are related to the following statements: 7A, comfort defining human trafficking; 7B, comfort recognizing trafficking in the clinical setting; 7C, comfort knowing how to refer a potential child who has been trafficked; 7D, comfort describing health consequences of human trafficking; 7E, perception of likelihood of seeing a trafficked child as a patient during residency.

## Discussion

We developed this resource to address the important need for an educational program teaching pediatric health care providers how to recognize and appropriately intervene for victims of child trafficking. Including the perspective of a trafficking survivor in our educational sessions was incredibly impactful for our participants. The survivor leader's input was invaluable for creating educational tools that could be used in pediatric programs to improve care for vulnerable children who are currently or at risk of becoming trafficked. Our findings indicate that a relatively brief (60 minutes) and interactive didactic session and point-of-care clinical reference tool positively impacted pediatric residents’ knowledge, skills, and self-reported attitudes related to caring for trafficked children.

Following the educational program, participants were better able to identify trafficked children presented in hypothetical case scenarios, as well as the correct initial management steps. Residents’ confidence levels related to recognizing and caring for trafficked children increased significantly following our program (Figure). These statistically significant increases in comfort/knowledge levels have not been seen in previous studies. For example, a study of a brief educational session amongst emergency medicine providers found a statistically significant increase only in self-rated knowledge of human trafficking but did not find significant increases in the importance of understanding human trafficking as a physician or in comfort with referring potentially trafficked persons.^5^

Though 37% of participants reported completing prior training regarding human trafficking, this subgroup still showed a statistically significant increase in self-reported comfort levels related to caring for trafficked children. This finding highlights the benefit of and need for continuing education on child trafficking for physicians periodically, regardless of previous training. Provider education should be implemented in a longitudinal manner so physicians can review the pertinent information over time to ensure effective identification of trafficked children.

A major strength of our educational program is that it was designed and presented in close collaboration with a survivor of human trafficking from our local community. The survivor leader served as an expert consultant and was compensated for their time and contributions.^13^ Close collaboration with this expert helped to ensure that the content and language in our training materials were accurate and sensitive to the trauma experienced by trafficking survivors. Incorporating the perspective of a trafficking survivor should be the standard for similar educational programs to ensure they are both survivor- and trauma-informed.^14^ Anecdotal feedback from participants reflected that the survivor's input and presence during our didactic had a powerful, positive impact on participants' perspective on the importance of this issue and on the importance of working with potential trafficking victims using a gentle, open, and trauma-informed approach. Feedback from the survivor expert indicated that they personally derived important benefits, including a sense of empowerment, from their involvement in this educational program.

The point-of-care reference tool introduced in our educational session was designed to support providers with a quick way to implement appropriate management steps when caring for suspected (or confirmed) trafficked children in the clinical setting. This novel tool can be referenced in the long term to reinforce crucial concepts central to recognition and intervention for trafficked children. It can both support physicians caring for children who have been trafficked and be easily adapted for use by providers at other institutions.

We encountered several challenges in the process of developing and implementing this curriculum. While our collaboration with the survivor expert greatly enhanced the content and interactivity of the program, we were challenged to ensure the project did not retraumatize or exploit them. We took several steps to mitigate this risk, including compensating them as an expert consultant, allowing them to choose their desired level of involvement, and debriefing after each session.

Scheduling the education session within a busy residency program was also a challenge. We utilized the protected resident education days, which allowed us to reach a higher number of residents but limited the length of sessions. Implementing the program within the resident education curriculum also addressed the challenge of sustainability, ensuring trafficking education would be continued in the future.

### Adaptation to Online Learning

This program can be adapted to online learning. The use of a virtual platform would improve the feasibility of including perspectives of trafficking survivors—as well as experts including social workers and law enforcement professionals—since participants can join from multiple locations. The ability to join without video may increase trafficking survivors' comfort in contributing to educational discussions while preserving their desired level of anonymity. If a virtual format is utilized, steps should be taken to maintain confidentiality regarding any patient information used during the program. Session pre- and postassessments could be distributed through an electronic survey system or through a virtual poll. Algorithm cards could be distributed by mail or electronically to participants.

### Limitations

The use of pre- and postassessment methodology carried limitations. Some participants submitted incomplete assessments, which limited our ability to conduct paired analyses. Given that the pre- and postassessments were identical, participants could have simply memorized answers from the preassessment rather than truly learning the content presented in our session. The postassessment was given immediately following the didactic session; therefore, our results do not reflect long-term knowledge retention. We observed that participant performance on two specific items of our assessment (items 4C and 5) was consistently poor, both before and after our educational program. While this observation may demonstrate that our session was not effective in training participants on these concepts, it could also be explained by poor wording and structure of these questions. These specific items were subsequently rewritten to improve clarity.

We acknowledge that participants may have selected the correct answers on certain knowledge assessment items due to test-taking bias arising from their awareness that the focus of the educational program was on human trafficking, which they learned through reviewing an informed consent sheet prior to the session. This limitation was addressed through the comparison of the pre- and postsession knowledge assessment data to account for baseline knowledge and biases. Lastly, knowledge related to the trauma-informed care Educational Objective 4 was not evaluated in the assessments.

### Conclusion

Our findings support that a survivor-informed educational program combined with a novel point-of-care clinical reference tool can lead to significant positive effects on pediatric residents’ knowledge, skills, and self-reported attitudes related to caring for trafficked children. This educational program is a feasible and effective method for training resident physicians to identify and intervene for trafficked children and can be easily adapted for use by other training programs and health care organizations.

## Appendices


Preceptor Guide.docxPediatric Human Trafficking Presentation.pptxAlgorithm Card Editable.pptxAlgorithm Card.pdfPre- and Postsession Knowledge Assessment.docxKnowledge Assessment with Answers.docx
*All appendices are peer reviewed as integral parts of the Original Publication.*

## References

[1] Curtis R, Terry K, Dank M, Dombrowski K, Khan B. The Commercial Sexual Exploitation of Children in New York City, Volume One: The CSEC Population in New York City: Size, Characteristics, and Needs. National Institute of Justice; 2008. Accessed June 21, 2021. https://www.ojp.gov/pdffiles1/nij/grants/225083.pdf

[2] Beck ME, Lineer MM, Melzer-Lange M, Simpson P, Nugent M, Rabbitt A. Medical providers’ understanding of sex trafficking and their experience with at-risk patients. Pediatrics. 2015;135(4):e895–e902. 10.1542/peds.2014-2814 25780076

[3] Greenbaum VJ. Child sex trafficking in the United States: challenges for the healthcare provider. PLoS Med. 2017;14(11):e1002439. 10.1371/journal.pmed.1002439 29166405 PMC5699805

[4] Grace AM, Ahn R, Konstantopoulos WM. Integrating curricula on human trafficking into medical education and residency training. JAMA Pediatr. 2014;168(9):793–794. 10.1001/jamapediatrics.2014.999 25048726

[5] Grace AM, Lippert S, Collins K, et al. Educating health care professionals on human trafficking. Pediatr Emerg Care. 2014;30(12):856–861. 10.1097/PEC.0000000000000287 25407038 PMC4392380

[6] *Global Estimates of Modern Slavery: Forced Labour and Forced Marriage*. International Labour Office; 2017. Accessed June 21, 2021. http://www.ilo.org/wcmsp5/groups/public/---dgreports/---dcomm/documents/publication/wcms_575479.pdf

[7] *Sex Trafficking in the U.S.: A Closer Look at U.S. Citizen Victims*. Polaris Project; 2015. Accessed June 21, 2021. https://polarisproject.org/wp-content/uploads/2019/09/us-citizen-sex-trafficking.pdf

[8] Hotline statistics. National Human Trafficking Hotline. Accessed January 12, 2021. https://humantraffickinghotline.org/states

[9] Zimmerman C, Hossain M, Yun K, Roche B, Morison L, Watts C. *Stolen Smiles: A Summary Report on the Physical and Psychological Health Consequences of Women and Adolescents Trafficked in Europe*. The London School of Hygiene & Tropical Medicine; 2006. Accessed June 21, 2021. https://www.icmec.org/wp-content/uploads/2015/10/Stolen-Smiles-Physical-and-Psych-Consequences-of-Traffic-Victims-in-Europe-Zimmerman.pdf/

[10] Lederer LJ, Wetzel CA. The health consequences of sex trafficking and their implications for identifying victims in healthcare facilities. Ann Health Law. 2014;23(1):61–91.

[11] SOAR to health and wellness training. Office on Trafficking in Persons. Updated September 24, 2019. Accessed June 21, 2021. https://www.acf.hhs.gov/otip/training/soar-health-and-wellness-training

[12] Fraley HE, Aronowitz T, Stoklosa HM. Systematic review of human trafficking educational interventions for health care providers. West J Nurs Res. 2020;42(2):131–142. 10.1177/0193945919837366 30924735

[13] Powers L, Paul N. The need for survivor-informed research to fight human trafficking. Delta 8.7. November 1, 2018. Accessed January 2019. https://delta87.org/2018/11/need-survivor-informed-research-fight-human-trafficking/

[14] Miller C, Greenbaum J, Napolitano K, et al. Assessment tool for health care provider human trafficking training. HEAL Trafficking. 2018. Accessed June 21, 2021. https://healtrafficking.org/2018/12/assessment-tool-for-health-care-provider-human-trafficking-training/

