# Supplementary material for: A Human Trafficking Educational Program and Point-of-Care Reference Tool for Pediatric Residents
Source: MedEdPORTAL. 2021 Sep 13;17:11179. doi: 10.15766/mep_2374-8265.11179 (PMC8435556; doi:10.15766/mep_2374-8265.11179)
Supplement: Supplementary file 1 — Preceptor Guide.docxPediatric Human Trafficking Presentation.pptxAlgorithm Card Editable.pptxAlgorithm Card.pdfPre- and Postsession Knowledge Assessment.docxKnowledge Assessment with Answers.docx [file mep_2374-8265.11179-s001.zip › A. Preceptor Guide.docx]

**Human Trafficking Education Preceptor Guide**

In today’s session, the preceptors should:

- Provide pre-surveys for participants to complete prior to the educational intervention
- Discuss human trafficking curriculum, health consequences of human trafficking, nuances of the algorithm, and trauma informed care as it applies to trafficked youth
- Provide and invite participant completion of post-session surveys
- Distribute and review point-of-care human trafficking algorithm cards for reference after the intervention

**Details of the Program**

The main goals of this teaching session are to introduce the trainees to the topic of commercial and sexual exploitation of children/human trafficking of children, explain its relevance to pediatrics, and instruct trainees on ways to appropriately and safely intervene to aide trafficked youth. Additionally, the concept of trauma informed-care as it applies to this patient population will be covered as well.

First, ask participants to complete the pre-session survey so as to gauge their baseline knowledge. This pre-session survey will help inform the preceptors on the increase in knowledge after the educational intervention. During the educational intervention, preceptors can engage learners in discussing common myths related to human trafficking, and explain through the presentation why those myths are actually untrue based on current evidence. Have the trainees engage in discussion of the clinical vignettes and vignette “answers.” Preceptors are encouraged to incorporate discussion of personal vignettes if the preceptors or learners have worked with survivors or have experience working with trafficked youth. Additionally, please incorporate statistics that are relevant to the local area of the educational setting. The point-of-care algorithm card and post-session assessment should be distributed at the end of the session. The post-session assessment should be scored against the answer sheet as well as compared to the pre-session survey to see what educational gaps were corrected.

- 5-10 minutes: completion of pre-session assessment
- 5-10 minutes: welcome and introduction of session presenters (including survivor leader if he/she is present)
- 40 minutes: interactive didactic session (guided by slideset)
- 1-2 minutes: distribution and review of algorithm cards
- 5-10 minutes: completion of post-session assessment

After the session, the preceptors can share optional supplementary readings to augment the educational intervention:

1. Zimmerman, C., Hossain, M., Yun, K., Roche, B., Morison, L., & Watts, C. (2009). The physical and psychological health consequences of women and adolescents trafficked in Europe. *The London School of Hygiene & Tropical Medicine*. Retrieved from http://genderviolence.lshtm.ac.uk/files/Stolen-Smiles-Trafficking-and-Health-2006.pdf
2. Greenbaum, J., & Crawford-Jakubiak, J. E. (2015). Child Sex Trafficking and Commercial Sexual Exploitation: Health Care Needs of Victims. *PEDIATRICS*, *135*(3). https://doi.org/10.1542/peds.2014-4138
3. Alpert, E. J. (n.d.). Human Trafficking: Guidebook on Identification, Assessment, and Response in the Health Care Setting.

**Effective Survivor Partnership**

Effectively and responsibly including survivor leadership is important to the impact of a human trafficking educational intervention. Survivor leadership will be able to inform the intervention on inclusion of survivor ad trauma sensitive language, as well as on content validity. A responsible and equal partnership between both parties is important to avoid re-traumatization and exploitation. The survivor leader should be appropriately compensated as well for their time and commitment to the program so as to reduce the cycle of exploitation they have overcome previously. Local anti-trafficking organizations can aide in partnership with survivors, but if the local community is lacking – national and regional organizations can be contacted. These organizations include:

1. National Human Trafficking Hotline Organization Directory: <https://humantraffickinghotline.org/training-resources/referral-directory>
2. National Survivor Network: <https://nationalsurvivornetwork.org/>

Clearly define roles/responsibilities of both parties, as well as ways to be accountable to each other. If additional resources are necessary, please review the optional reading for following best practices for working with survivors of human trafficking when considering creating this collaboration:

- Trauma Informed Code of Conduct for Working with Survivors of Human Trafficking: <http://www.helenbamber.org/wp-content/uploads/2019/01/Trauma-Informed-Code-of-Conduct.pdf>

**Trauma Informed Care**

Human trafficking and other nuances of trauma that are discussed in this presentation can be triggering for trainees and faculty alike that may be trauma survivors. The material in this presentation and discussing various forms of trauma can evoke strong emotional and post-traumatic responses. Please contact have the trainees/faculty contact the preceptors for support in these circumstances or refer to the resources on the assessment surveys. Allow individuals to participate to the level of their comfort, leaving as necessary.

The PEARR tool can be provided to your participants for guidance on effective ways to interview and engage with trafficked youth in a trauma informed manner. <https://www.dignityhealth.org/hello-humankindness/human-trafficking/victim-centered-and-trauma-informed/using-the-pearr-tool>

**Further Training Resources For Participants and Preceptors**

Department of Health and Human Services SOAR Training: <https://www.acf.hhs.gov/otip/training/soar-to-health-and-wellness-training>

American Medical Women’s Association Learn to Identify and Fight Trafficking (LIFT) training: <https://www.amwa-doc.org/our-work/initiatives/human-trafficking/>

HEAL Trafficking Compendium of Education Resources for Health Professionals: <https://healtrafficking.org/resources/compendium-of-educational-and-training-resources-for-health-professionals/>

**Adaptation to Online Learning**

This education session can be easily adapted to online learning. If using an online video meeting system such as Zoom, the information can be presented as if this were a live session. The pre- and post-surveys can either be distributed through an institutional survey system, or as a virtual poll before and after the session to obtain real time results. Algorithm cards would need to be distributed in person, however, and may be easily done through the participants’ mailboxes.
