# Supplementary material for: A Human Trafficking Educational Program and Point-of-Care Reference Tool for Pediatric Residents
Source: MedEdPORTAL. 2021 Sep 13;17:11179. doi: 10.15766/mep_2374-8265.11179 (PMC8435556; doi:10.15766/mep_2374-8265.11179)
Supplement: Supplementary file 1 — Preceptor Guide.docxPediatric Human Trafficking Presentation.pptxAlgorithm Card Editable.pptxAlgorithm Card.pdfPre- and Postsession Knowledge Assessment.docxKnowledge Assessment with Answers.docx [file mep_2374-8265.11179-s001.zip › B. Pediatric Human Trafficking Presentation.pptx]

## Slide 1
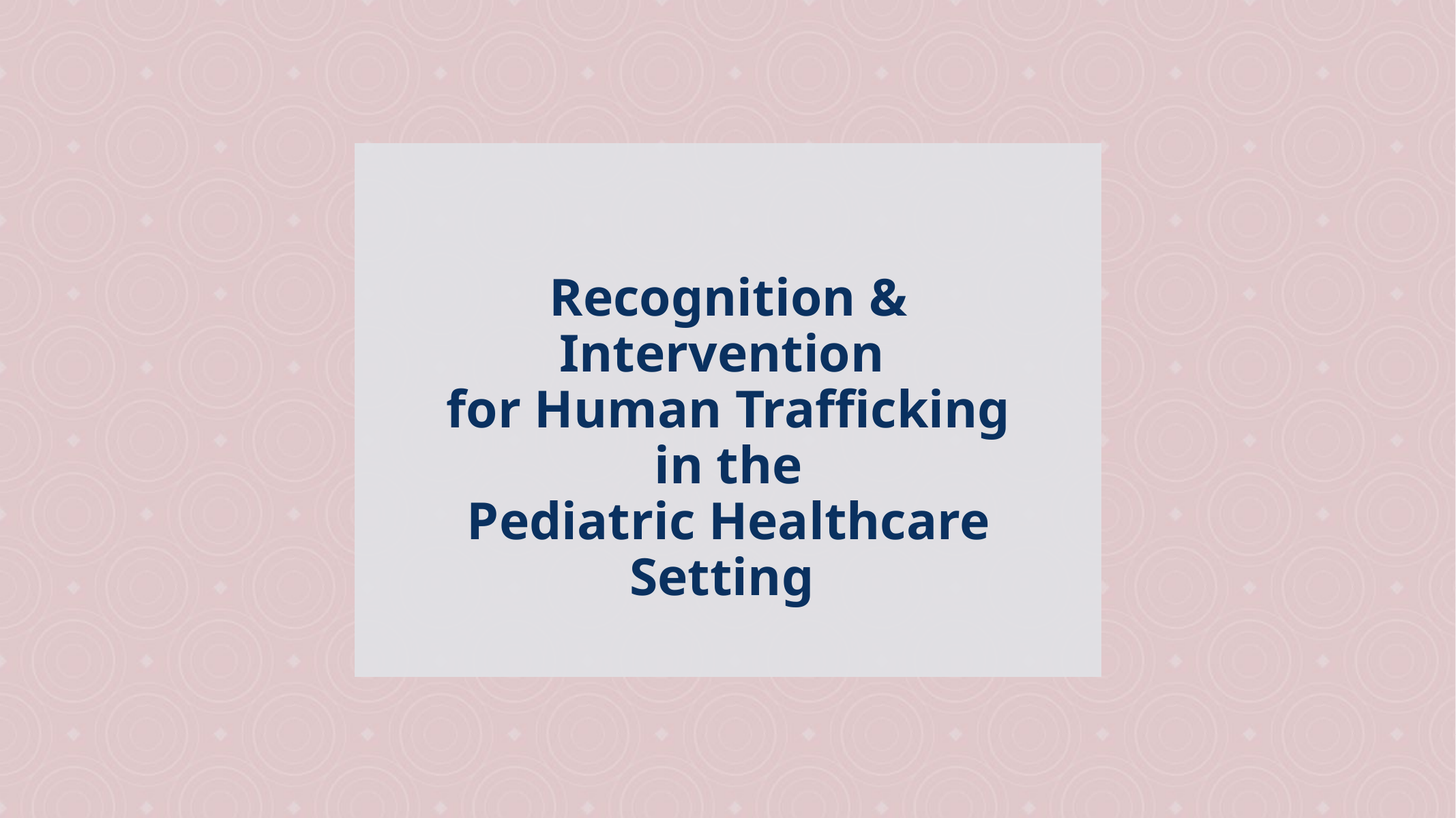

Recognition & Intervention
for Human Trafficking
in the
Pediatric Healthcare Setting

## Slide 2
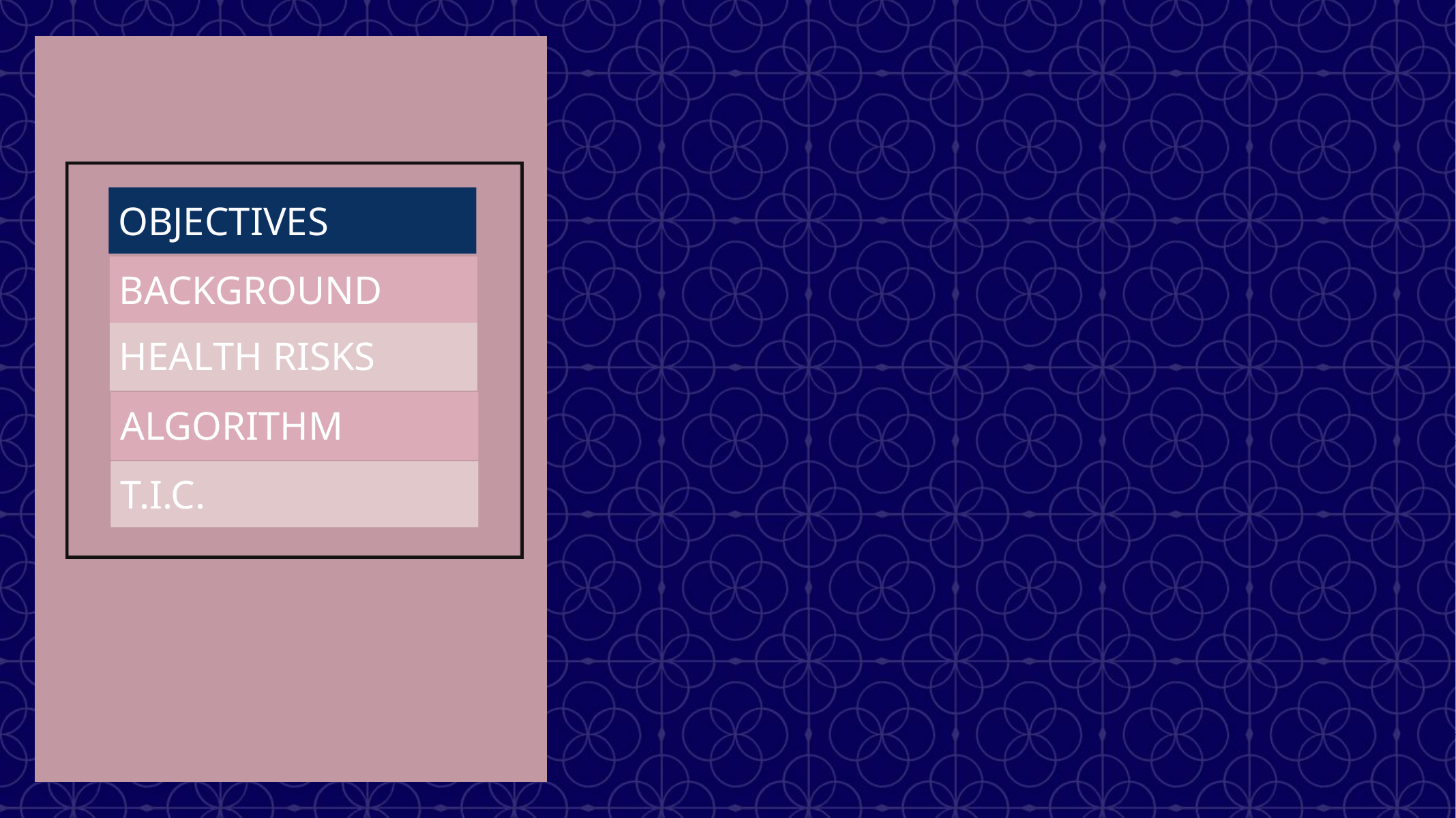

OBJECTIVES
BACKGROUND
HEALTH RISKS
ALGORITHM
T.I.C.

## Slide 3
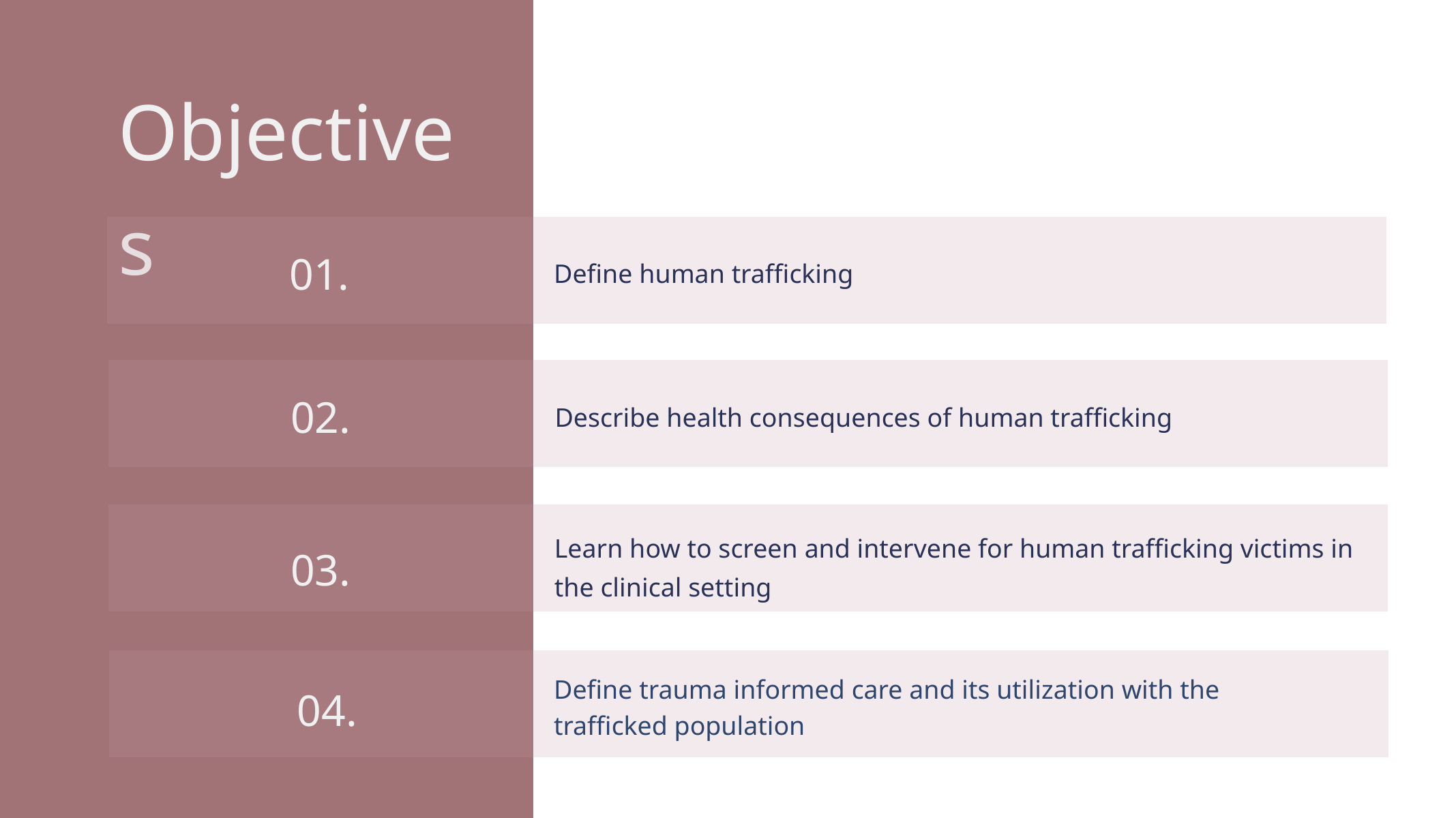

Objectives
01.
Define human trafficking
02.
Describe health consequences of human trafficking
Learn how to screen and intervene for human trafficking victims in the clinical setting
03.
Define trauma informed care and its utilization with the trafficked population
04.

## Slide 4
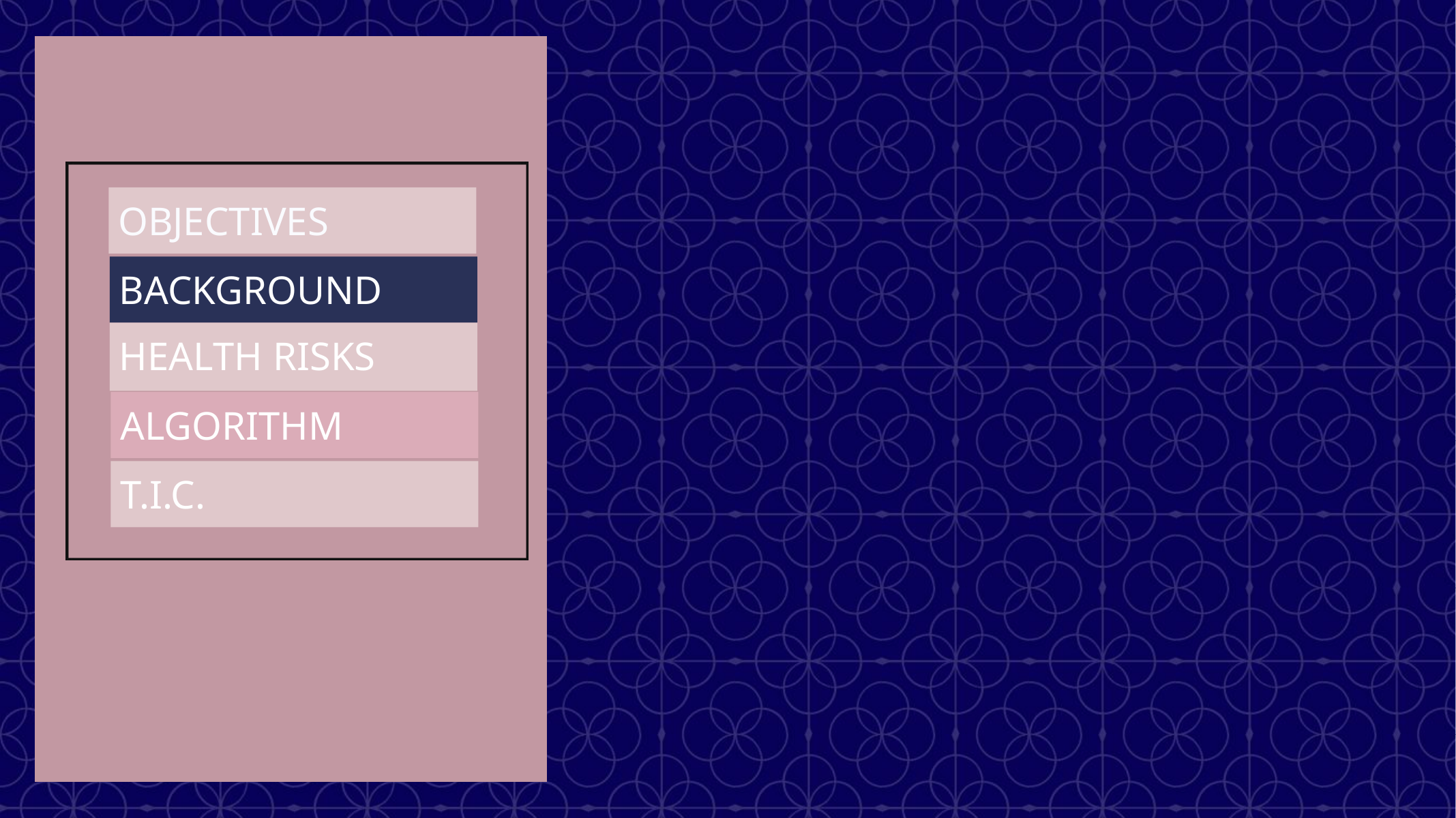

OBJECTIVES
BACKGROUND
HEALTH RISKS
ALGORITHM
T.I.C.

## Slide 5
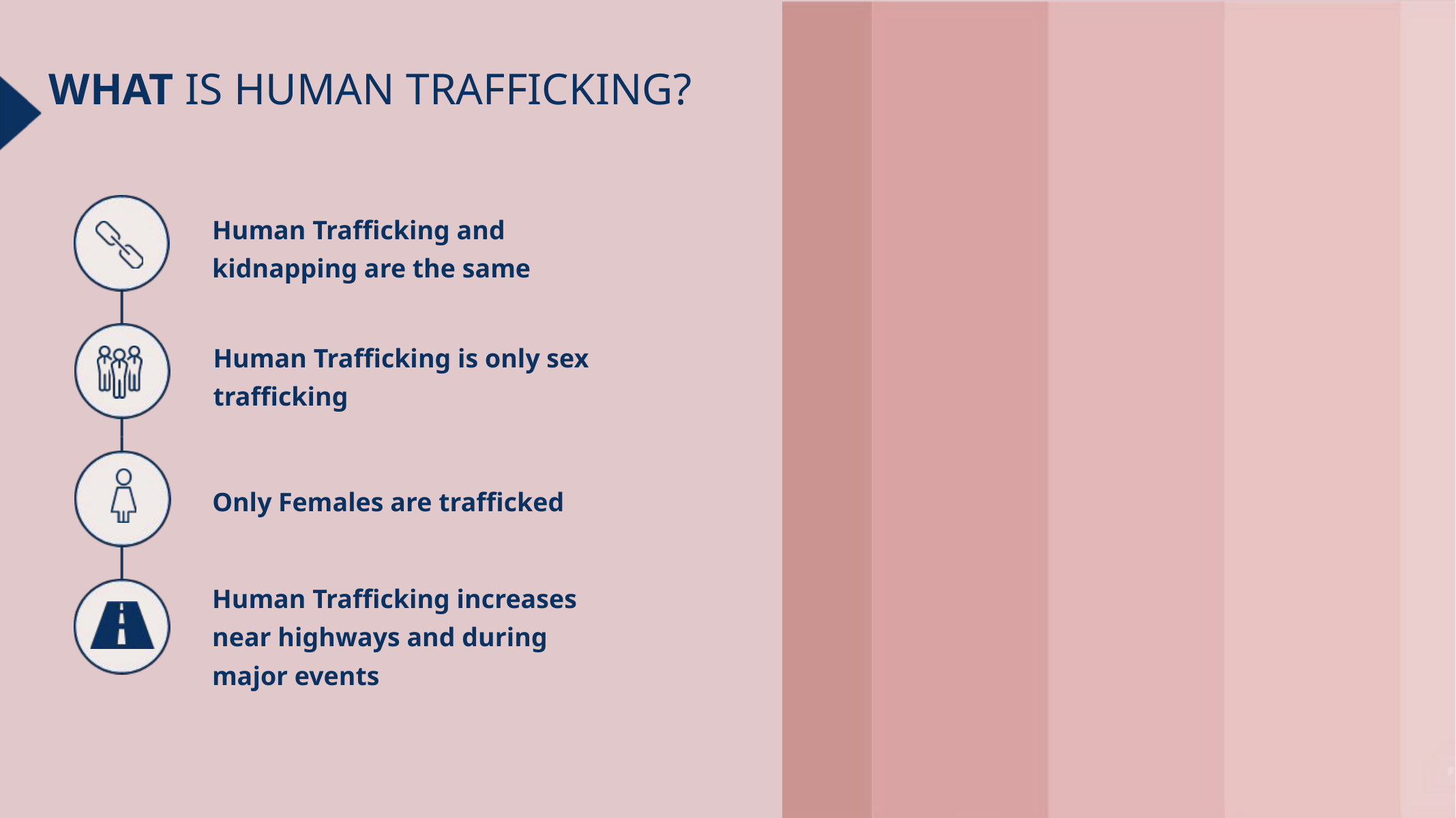

WHAT IS HUMAN TRAFFICKING?
Human Trafficking and kidnapping are the same
Human Trafficking is only sex trafficking
Only Females are trafficked
Human Trafficking increases near highways and during major events

## Slide 6
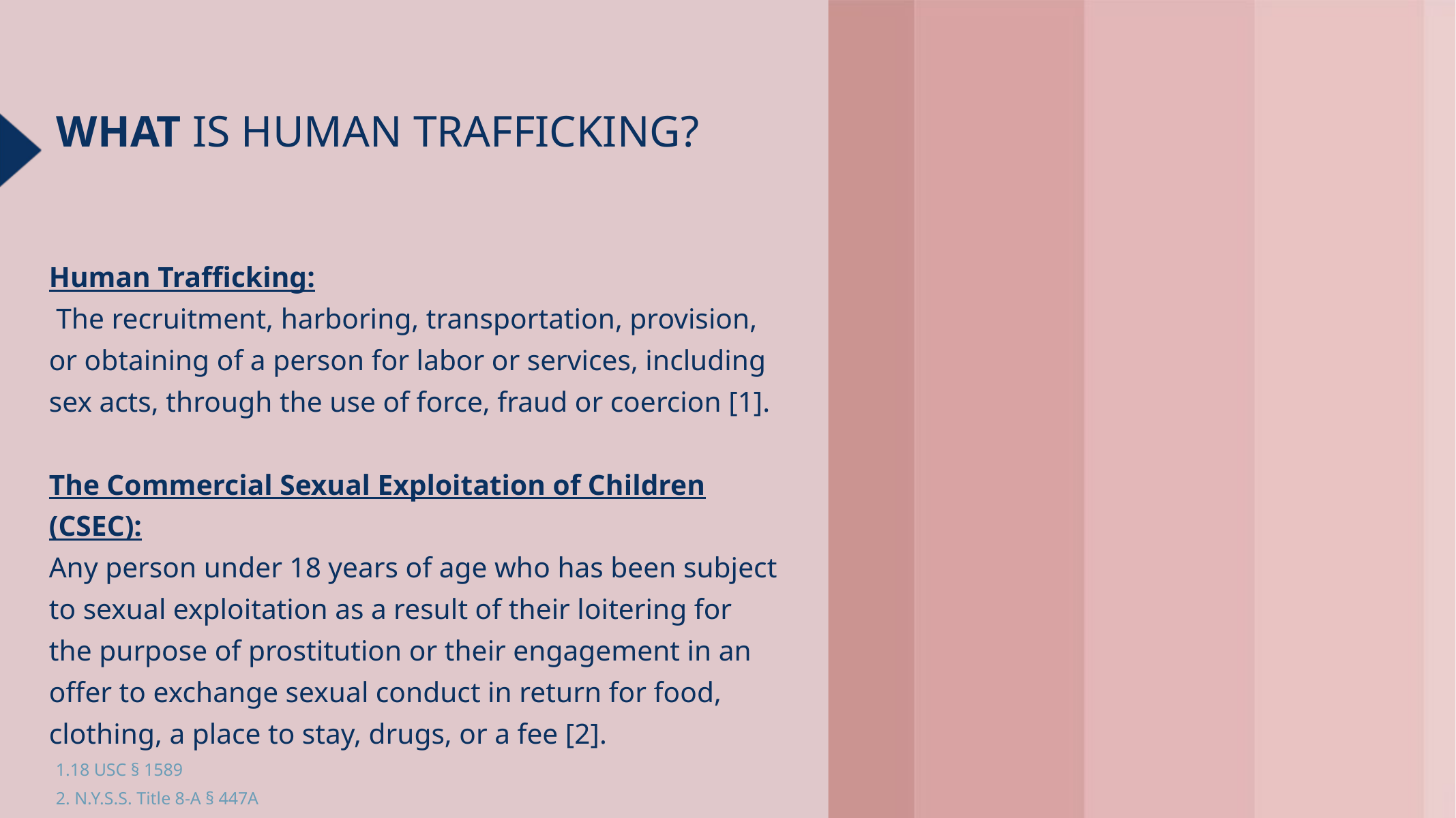

WHAT IS HUMAN TRAFFICKING?
Human Trafficking:
 The recruitment, harboring, transportation, provision, or obtaining of a person for labor or services, including sex acts, through the use of force, fraud or coercion [1].
The Commercial Sexual Exploitation of Children (CSEC):
Any person under 18 years of age who has been subject to sexual exploitation as a result of their loitering for the purpose of prostitution or their engagement in an offer to exchange sexual conduct in return for food, clothing, a place to stay, drugs, or a fee [2].
1.18 USC § 1589
2. N.Y.S.S. Title 8-A § 447A

## Slide 7
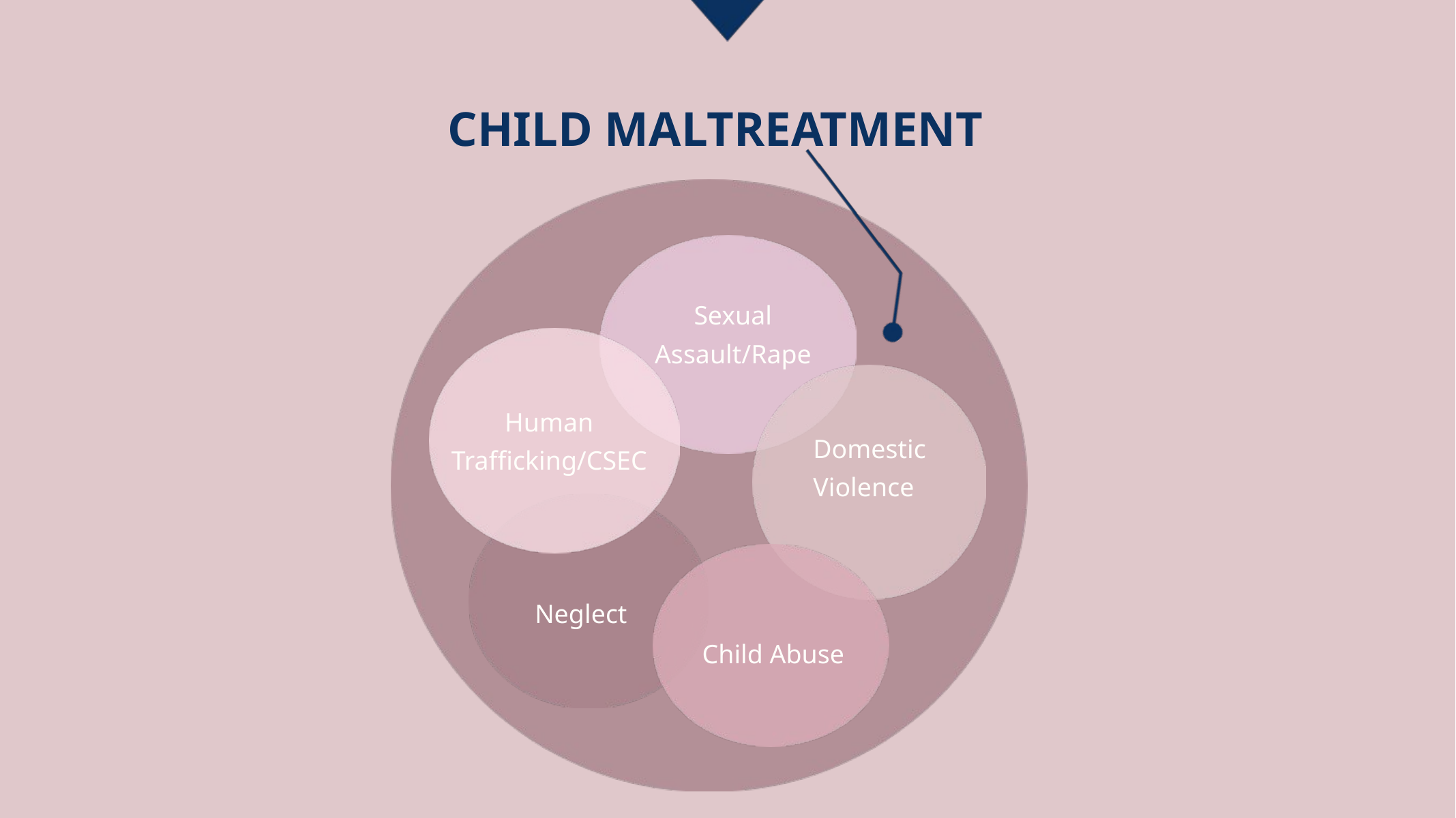

CHILD MALTREATMENT
Sexual Assault/Rape
Human Trafficking/CSEC
Domestic Violence
Neglect
Child Abuse

## Slide 8
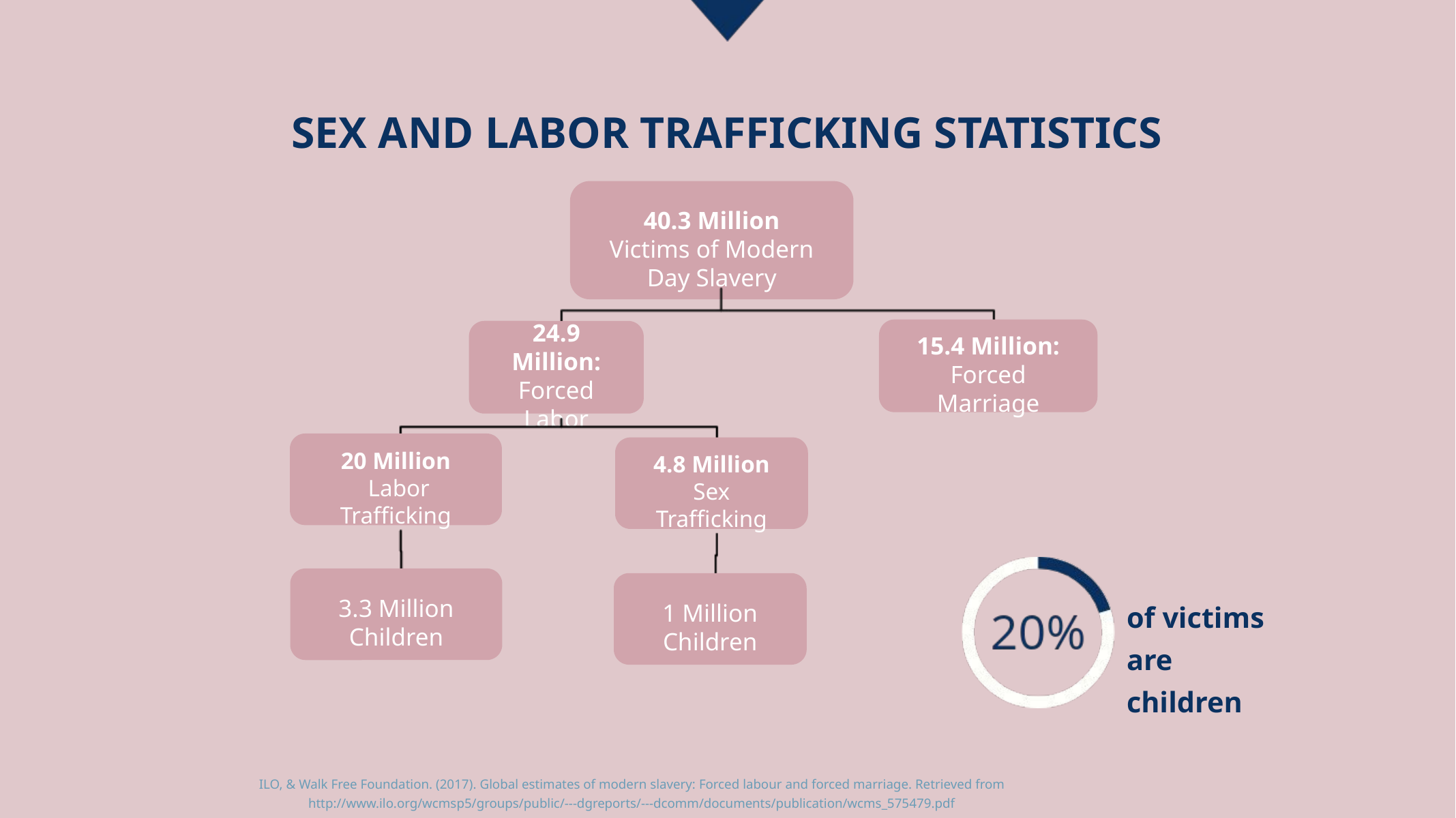

SEX AND LABOR TRAFFICKING STATISTICS
40.3 Million
Victims of Modern Day Slavery
15.4 Million: Forced Marriage
24.9 Million: Forced Labor
20 Million
 Labor Trafficking
4.8 Million
Sex Trafficking
of victims
are children
3.3 Million Children
1 Million Children
ILO, & Walk Free Foundation. (2017). Global estimates of modern slavery: Forced labour and forced marriage. Retrieved from http://www.ilo.org/wcmsp5/groups/public/---dgreports/---dcomm/documents/publication/wcms_575479.pdf

## Slide 9
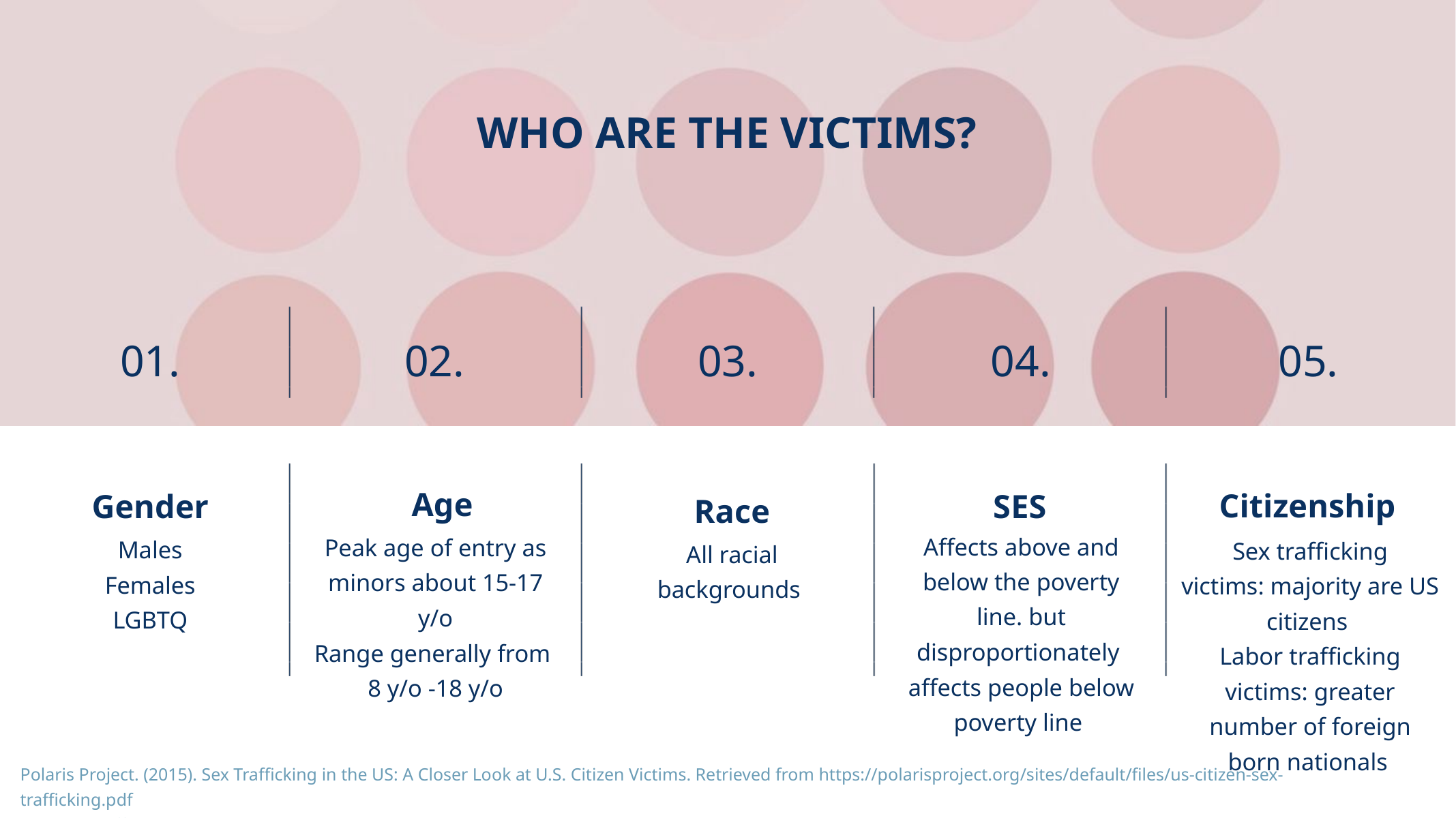

WHO ARE THE VICTIMS?
01.
02.
03.
04.
05.
Age
Citizenship
Gender
SES
Race
Affects above and below the poverty line. but disproportionately  affects people below poverty line
Peak age of entry as minors about 15-17 y/o
Range generally from
8 y/o -18 y/o
Males
Females
LGBTQ
Sex trafficking victims: majority are US citizens
Labor trafficking victims: greater number of foreign born nationals
All racial backgrounds
Polaris Project. (2015). Sex Trafficking in the US: A Closer Look at U.S. Citizen Victims. Retrieved from https://polarisproject.org/sites/default/files/us-citizen-sex-trafficking.pdf
Counter-Trafficking Data Collaborative (CTDC), [February, 2020].

## Slide 10
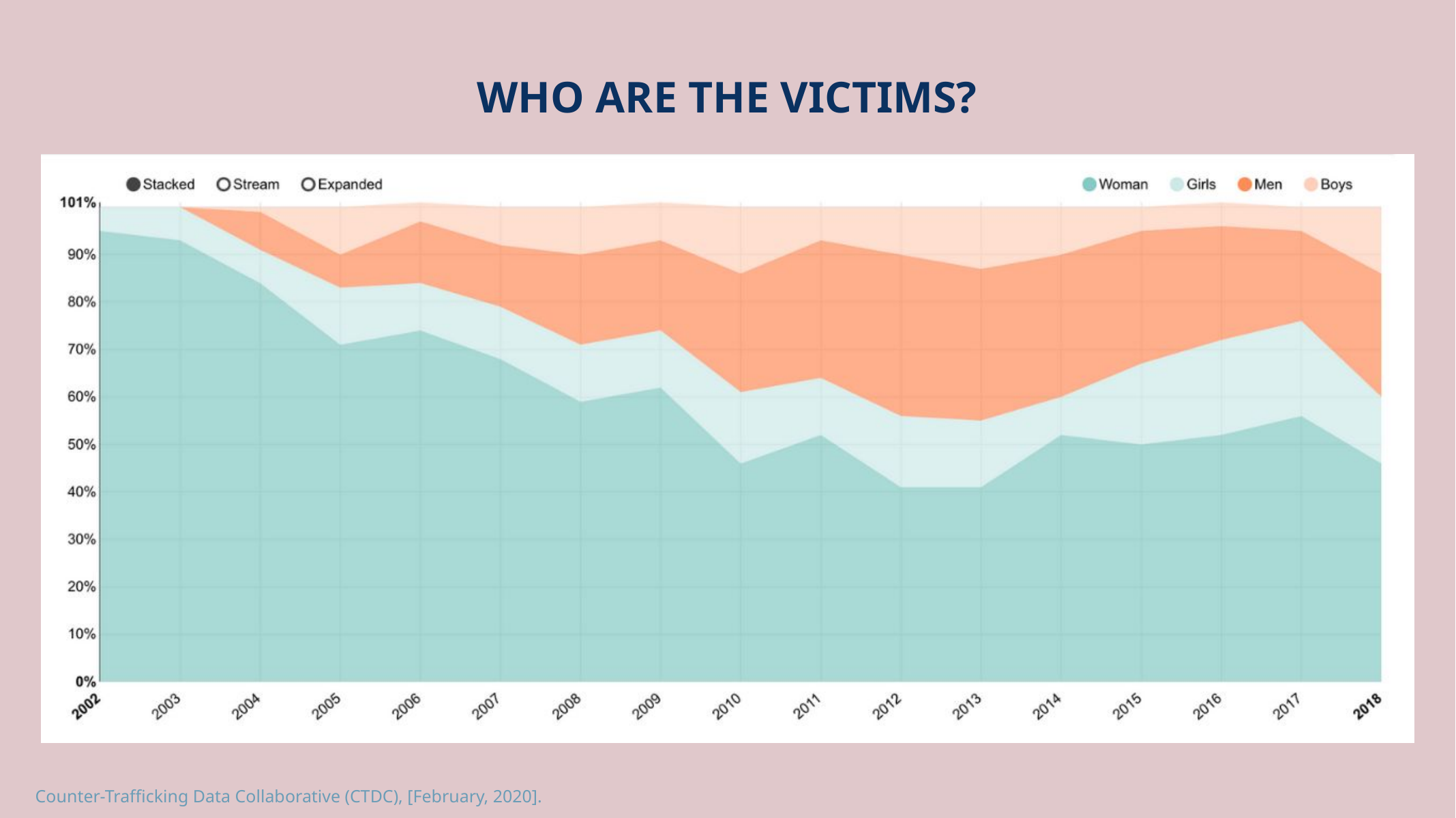

WHO ARE THE VICTIMS?
Counter-Trafficking Data Collaborative (CTDC), [February, 2020].

## Slide 11
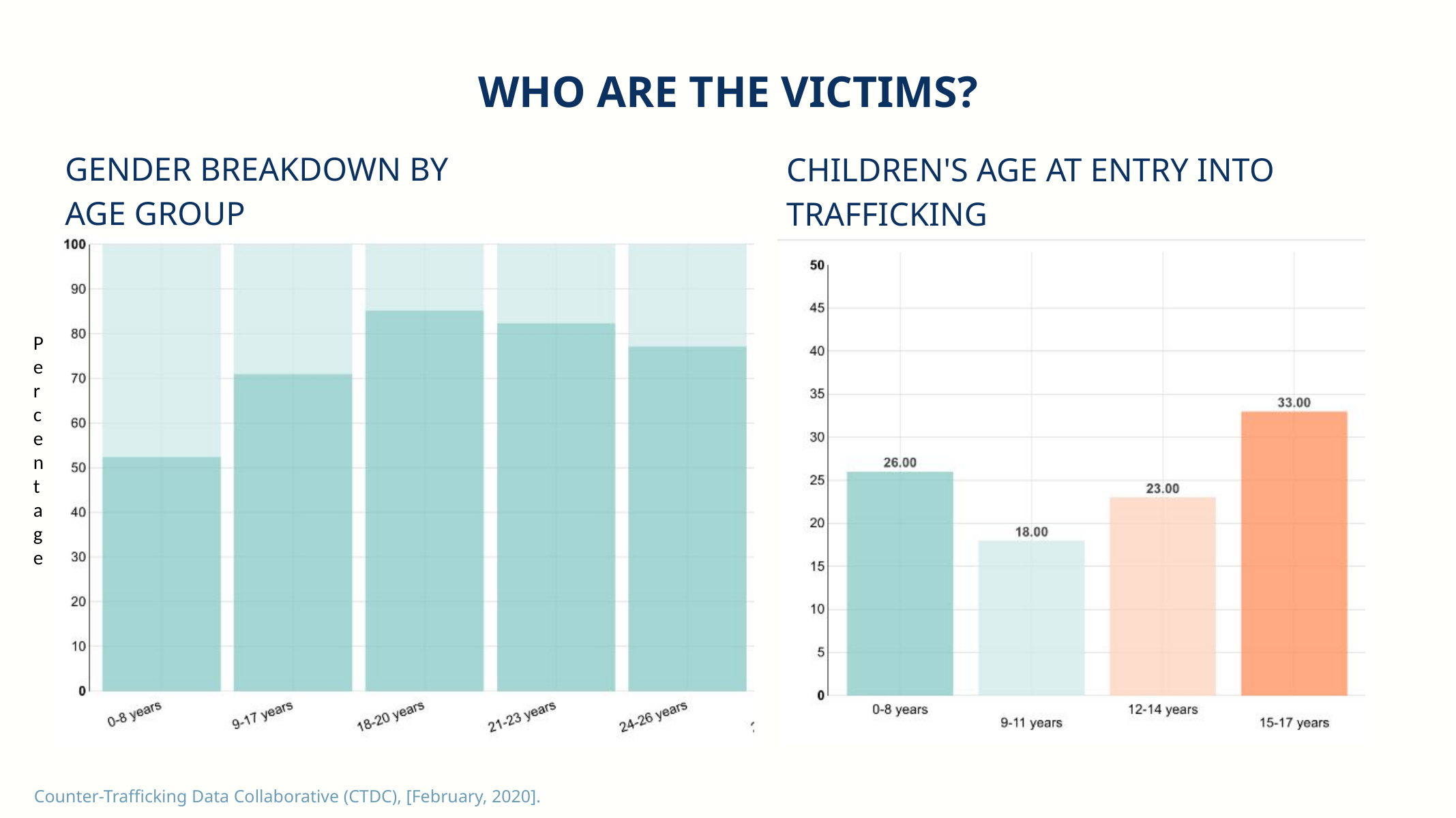

WHO ARE THE VICTIMS?
GENDER BREAKDOWN BY AGE GROUP
CHILDREN'S AGE AT ENTRY INTO TRAFFICKING
Percentage
Counter-Trafficking Data Collaborative (CTDC), [February, 2020].

## Slide 12
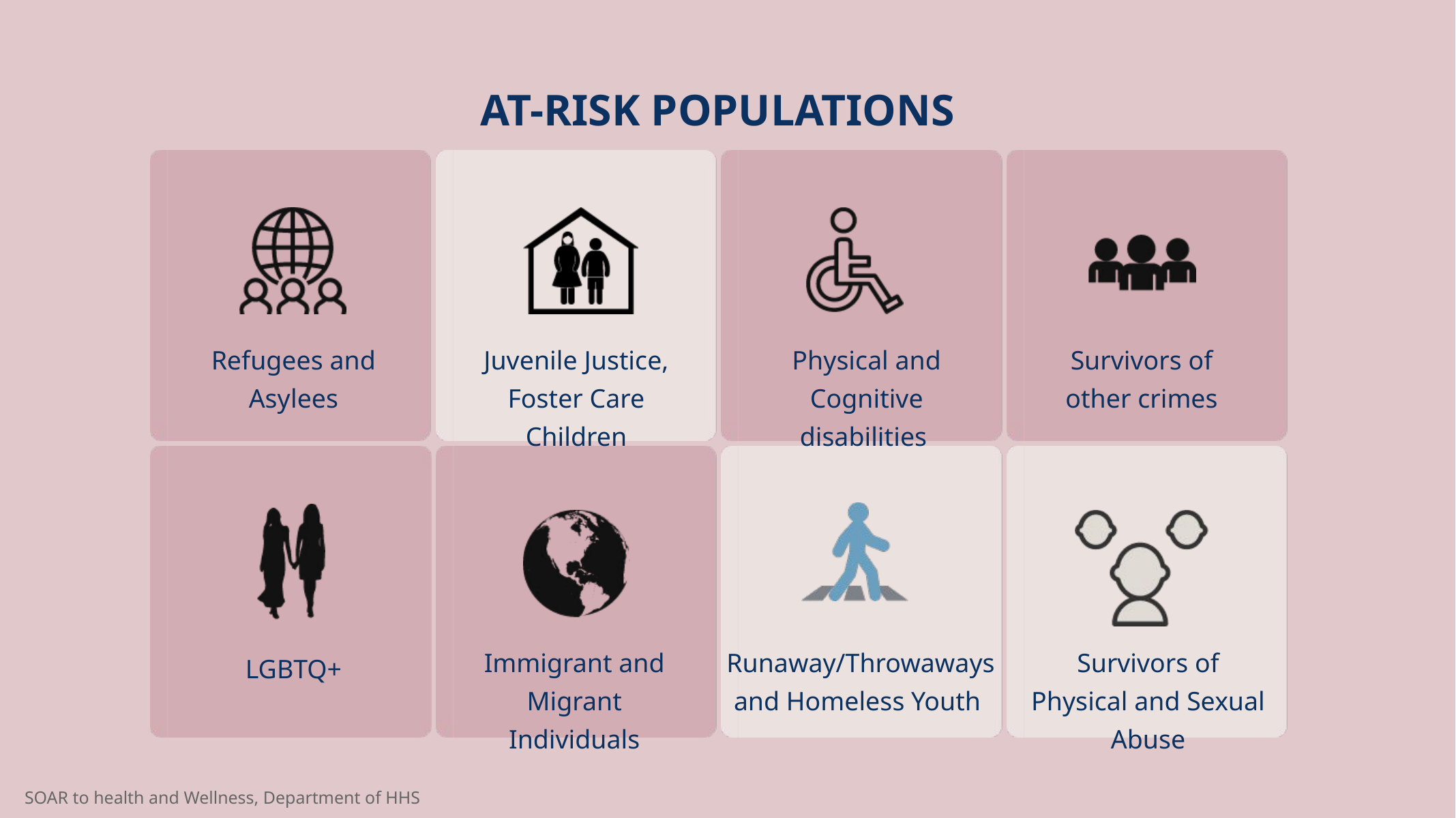

AT-RISK POPULATIONS
Refugees and Asylees
Juvenile Justice, Foster Care Children
Physical and Cognitive disabilities
Survivors of other crimes
Immigrant and Migrant Individuals
Runaway/Throwaways and Homeless Youth
Survivors of Physical and Sexual Abuse
LGBTQ+
SOAR to health and Wellness, Department of HHS

## Slide 13
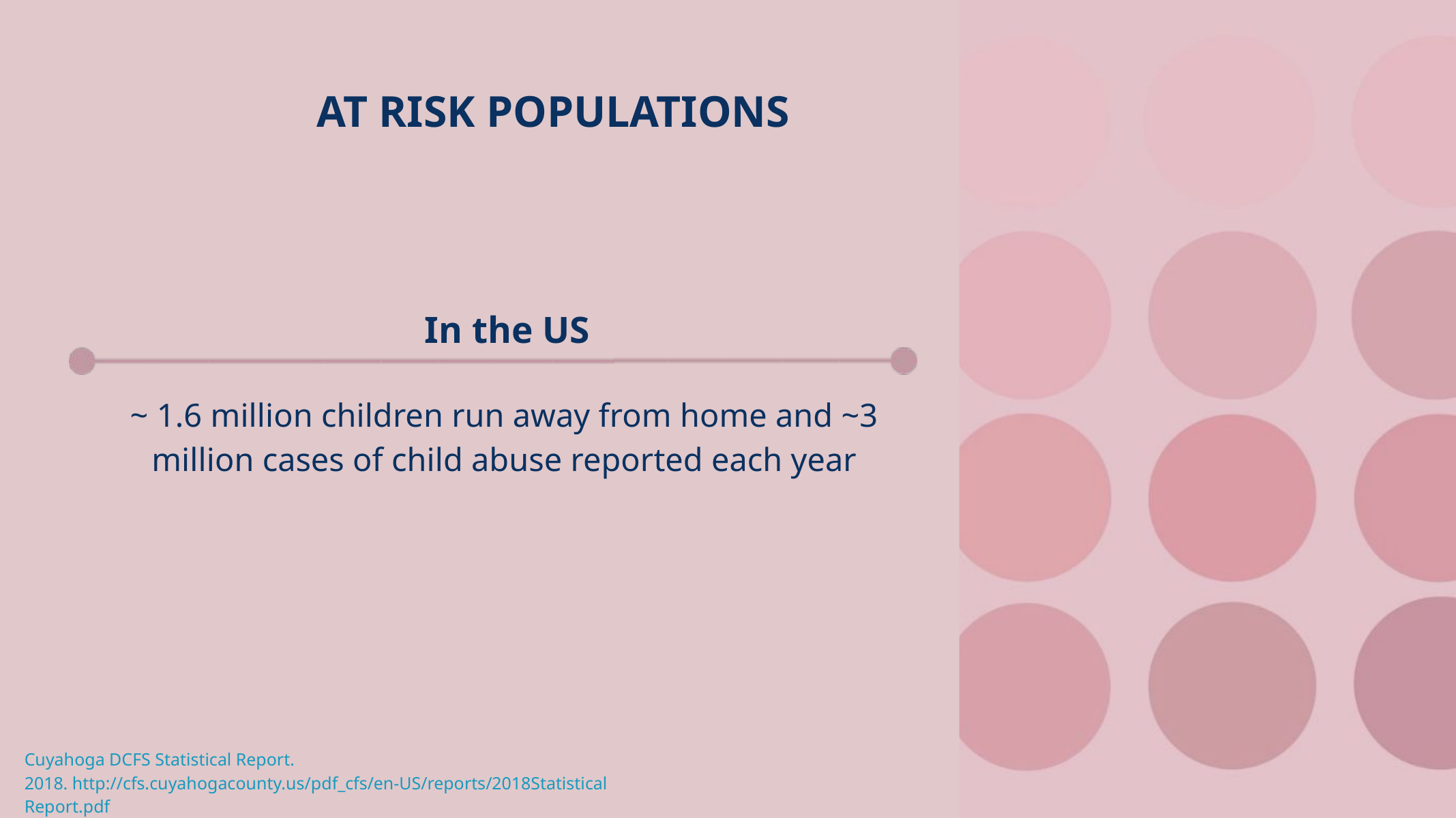

AT RISK POPULATIONS
In the US
~ 1.6 million children run away from home and ~3 million cases of child abuse reported each year
Cuyahoga DCFS Statistical Report. 2018. http://cfs.cuyahogacounty.us/pdf_cfs/en-US/reports/2018StatisticalReport.pdf

## Slide 14
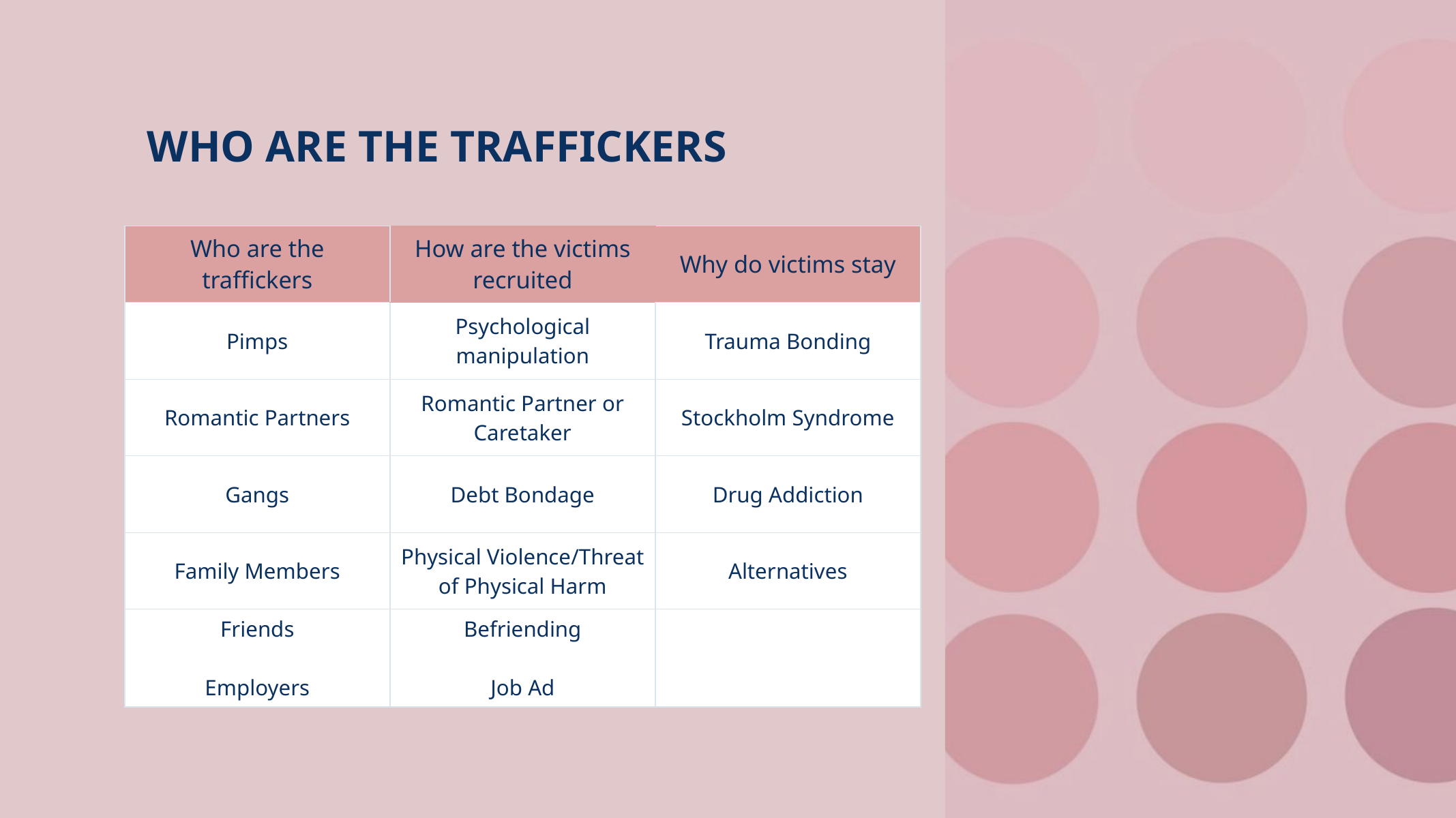

WHO ARE THE TRAFFICKERS
| Who are the traffickers | How are the victims recruited | Why do victims stay |
| --- | --- | --- |
| Pimps | Psychological manipulation | Trauma Bonding |
| Romantic Partners | Romantic Partner or Caretaker | Stockholm Syndrome |
| Gangs | Debt Bondage | Drug Addiction |
| Family Members | Physical Violence/Threat of Physical Harm | Alternatives |
| Friends   Employers | Befriending   Job Ad | |

## Slide 15
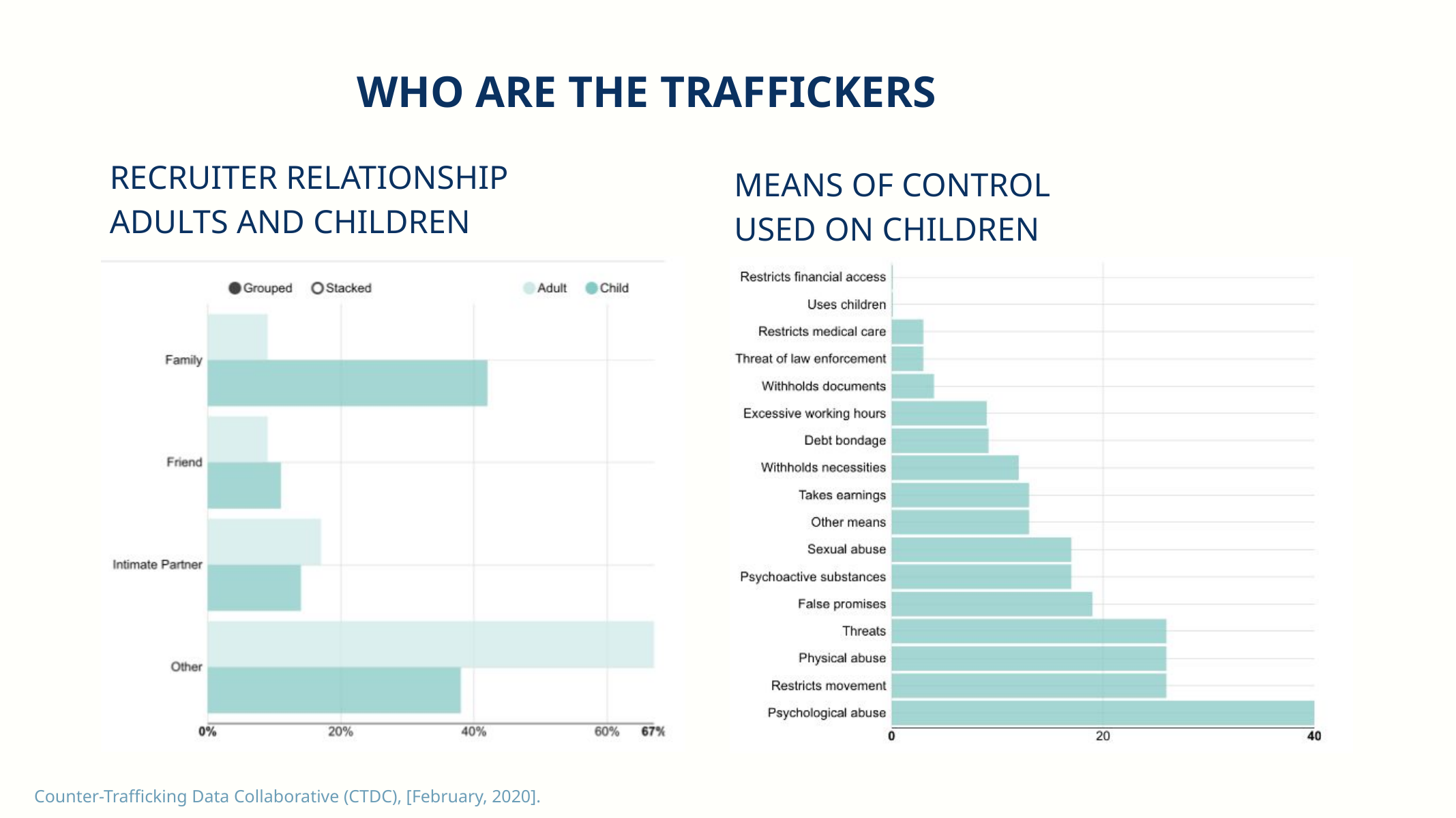

WHO ARE THE TRAFFICKERS
RECRUITER RELATIONSHIP ADULTS AND CHILDREN
MEANS OF CONTROL USED ON CHILDREN
Counter-Trafficking Data Collaborative (CTDC), [February, 2020].

## Slide 16
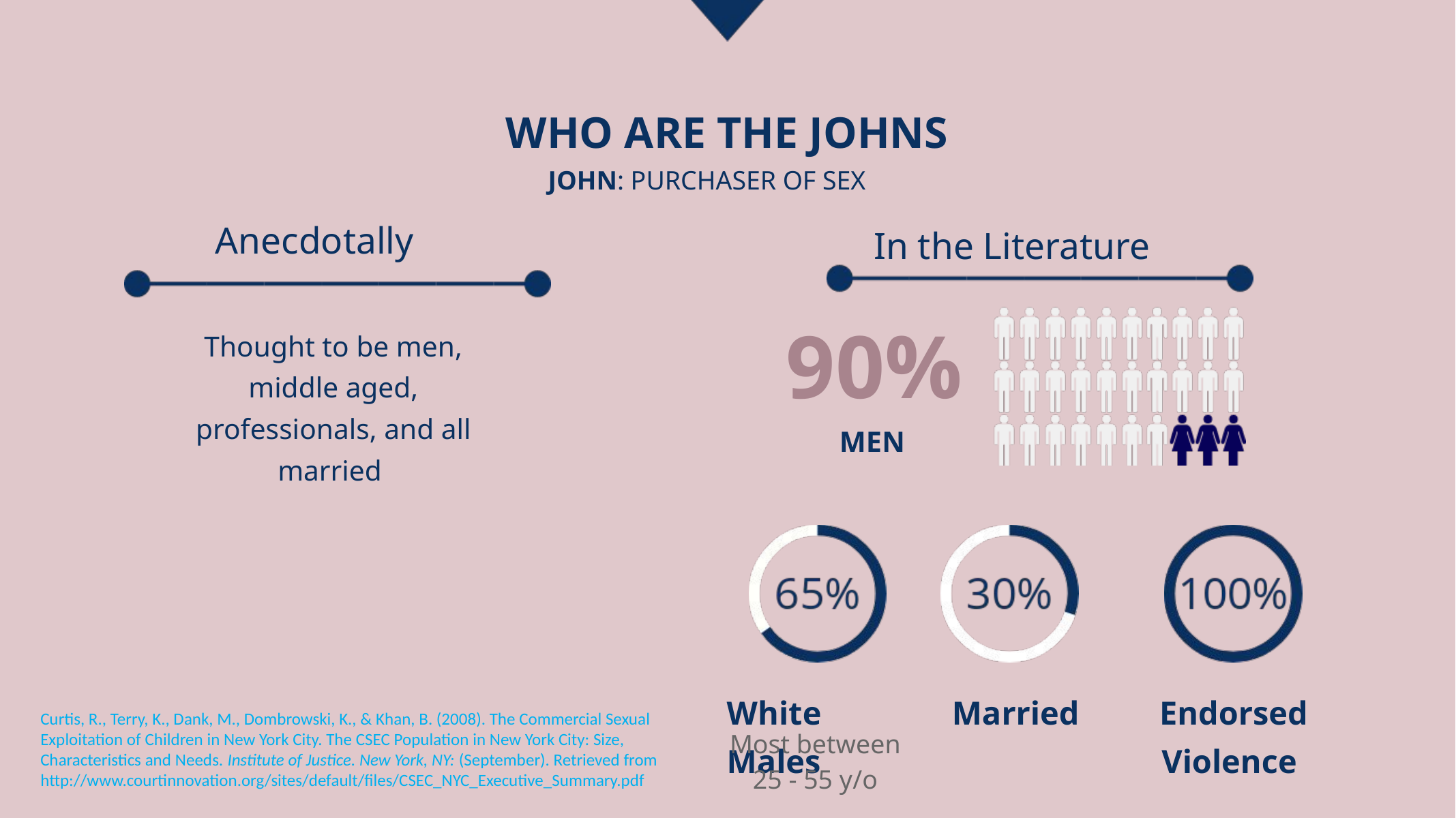

WHO ARE THE JOHNS
JOHN: PURCHASER OF SEX
Anecdotally
Thought to be men, middle aged, professionals, and all married
In the Literature
90%
MEN
White Males
    Married
Endorsed Violence
Most between 25 - 55 y/o
Curtis, R., Terry, K., Dank, M., Dombrowski, K., & Khan, B. (2008). The Commercial Sexual Exploitation of Children in New York City. The CSEC Population in New York City: Size, Characteristics and Needs. Institute of Justice. New York, NY: (September). Retrieved from http://www.courtinnovation.org/sites/default/files/CSEC_NYC_Executive_Summary.pdf

## Slide 17
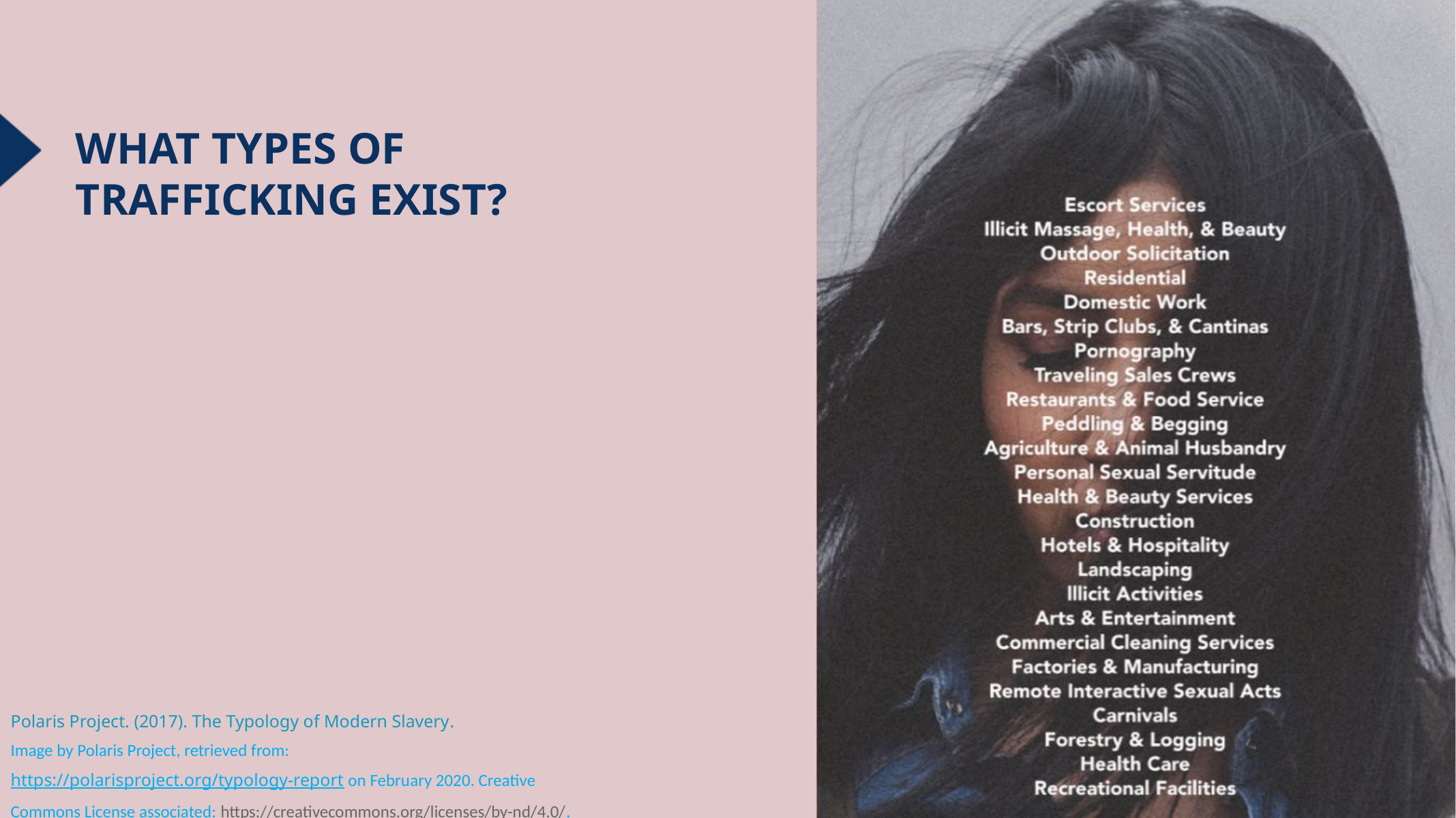

WHAT TYPES OF TRAFFICKING EXIST?
Polaris Project. (2017). The Typology of Modern Slavery.
Image by Polaris Project, retrieved from: https://polarisproject.org/typology-report on February 2020. Creative Commons License associated: https://creativecommons.org/licenses/by-nd/4.0/.

## Slide 18
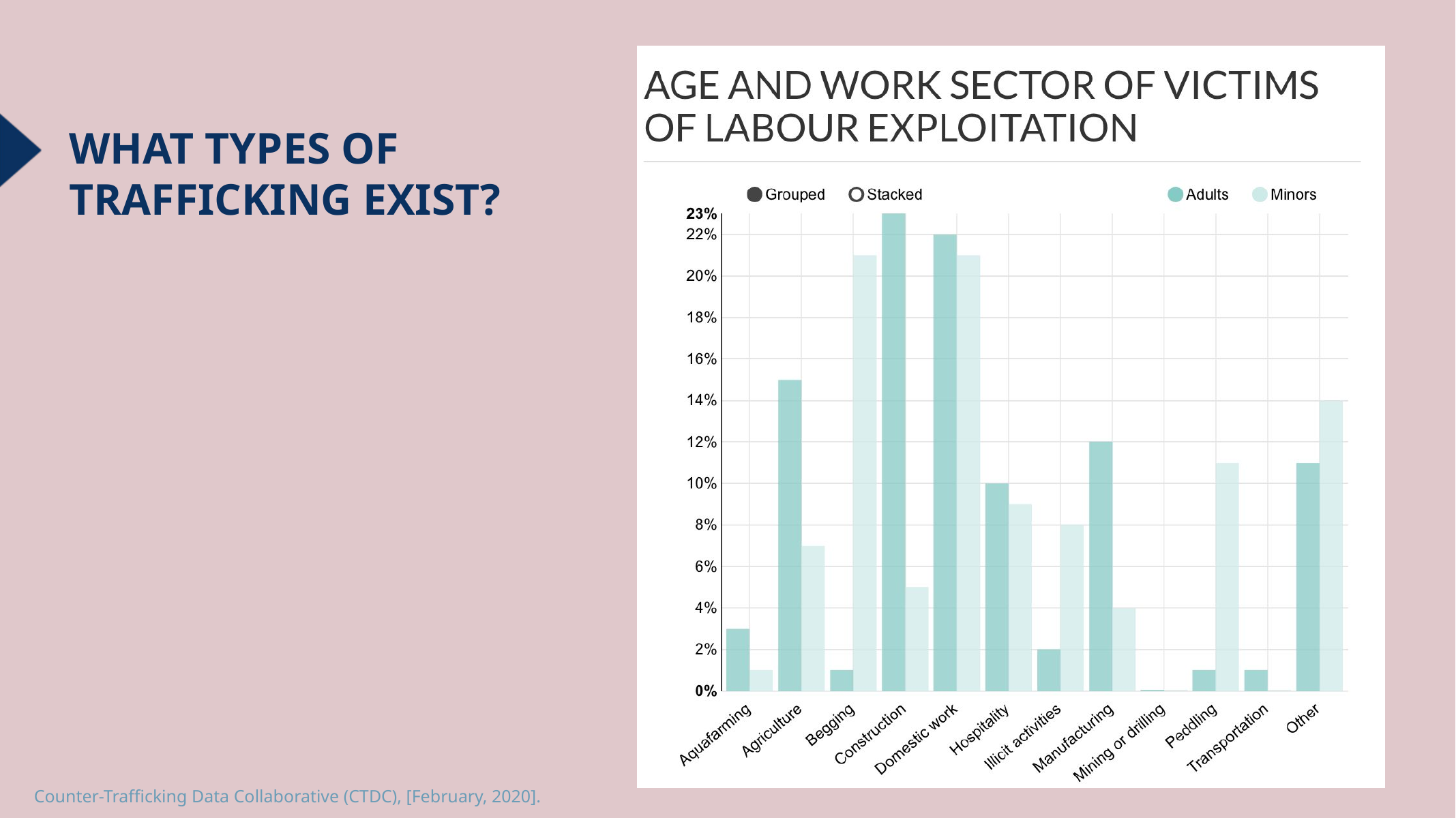

WHAT TYPES OF TRAFFICKING EXIST?
Counter-Trafficking Data Collaborative (CTDC), [February, 2020].

## Slide 19
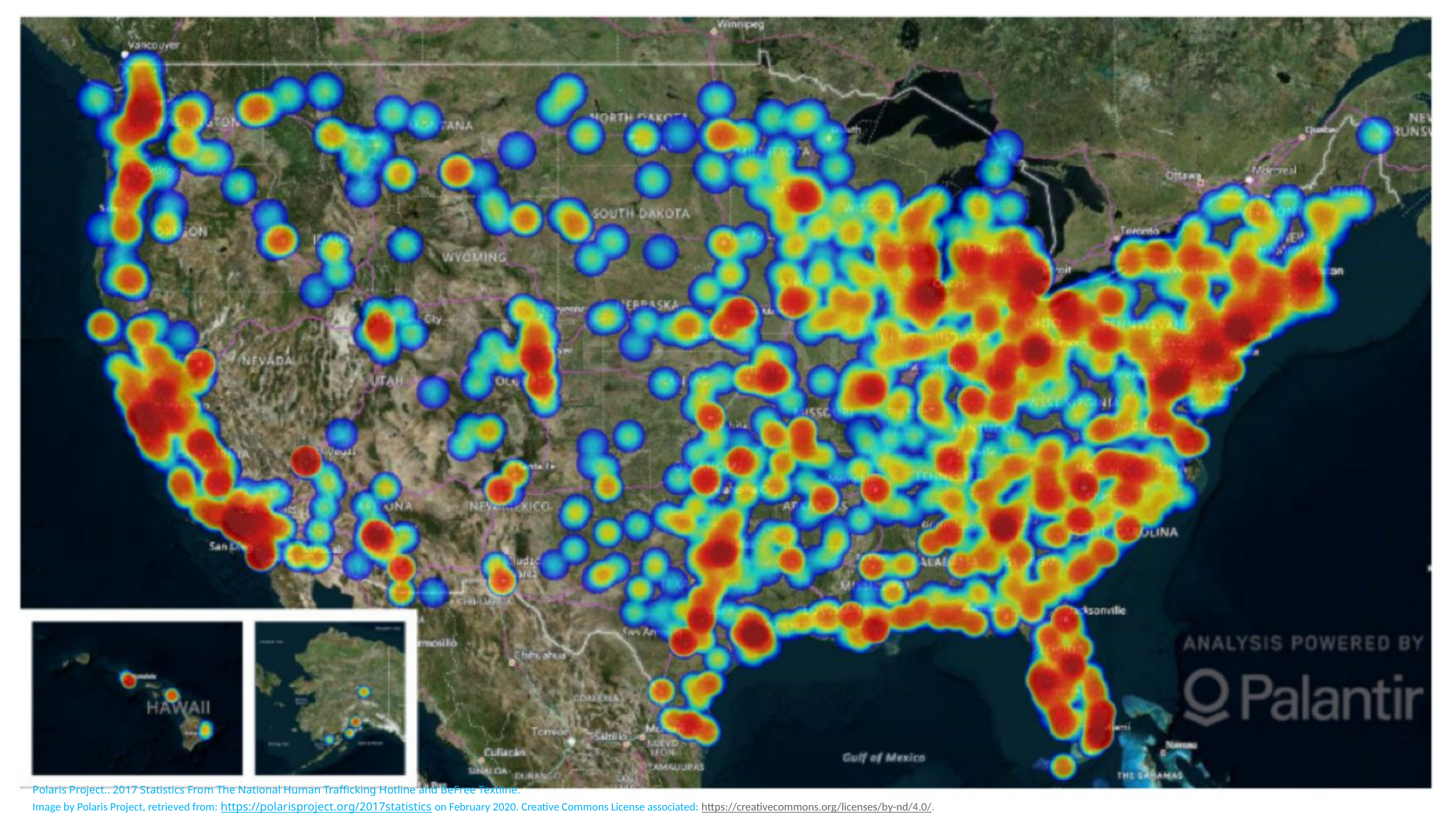

Polaris Project.. 2017 Statistics From The National Human Trafficking Hotline and BeFree Textline.
Image by Polaris Project, retrieved from: https://polarisproject.org/2017statistics on February 2020. Creative Commons License associated: https://creativecommons.org/licenses/by-nd/4.0/.

## Slide 20
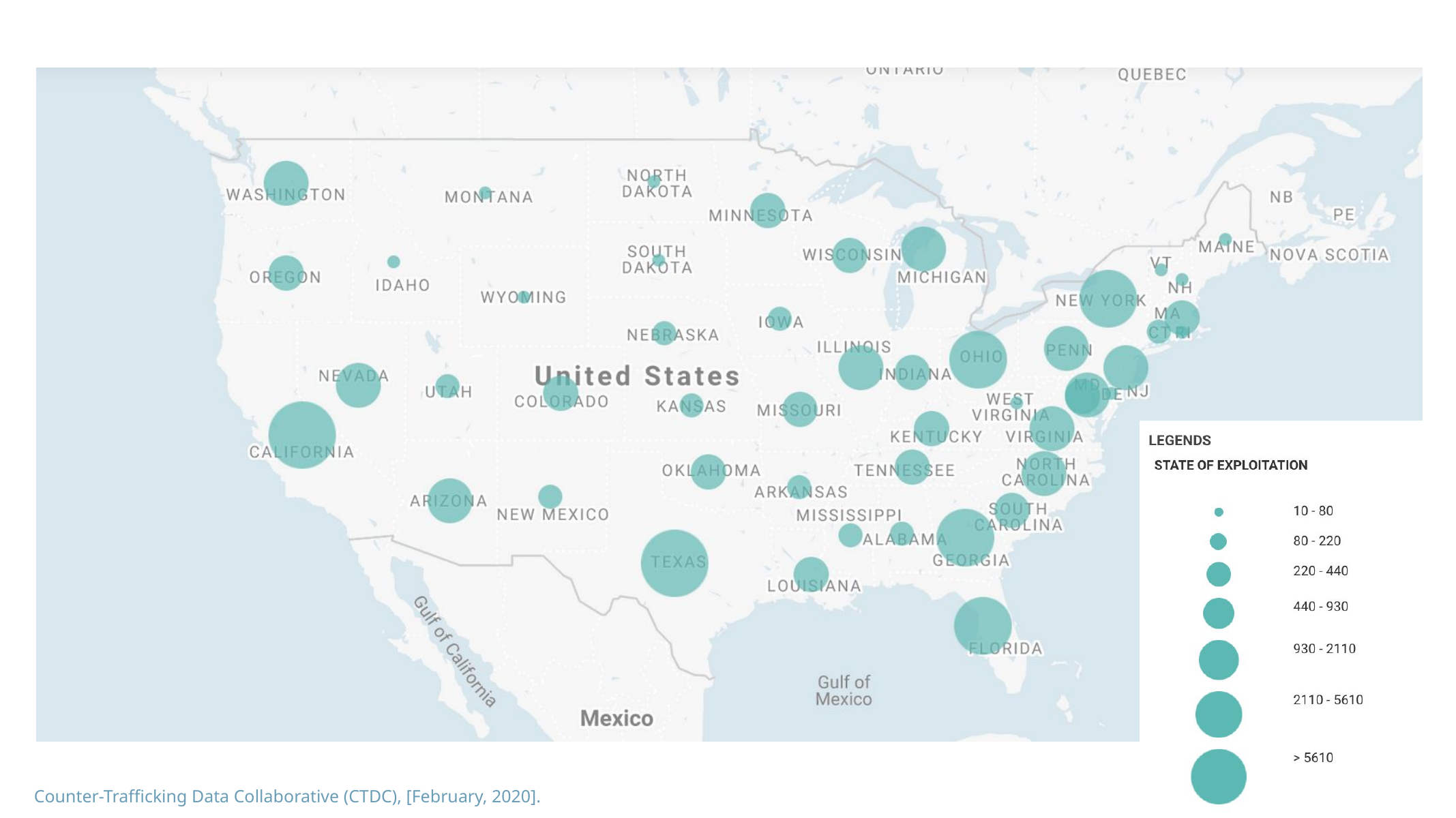

Counter-Trafficking Data Collaborative (CTDC), [February, 2020].

## Slide 21
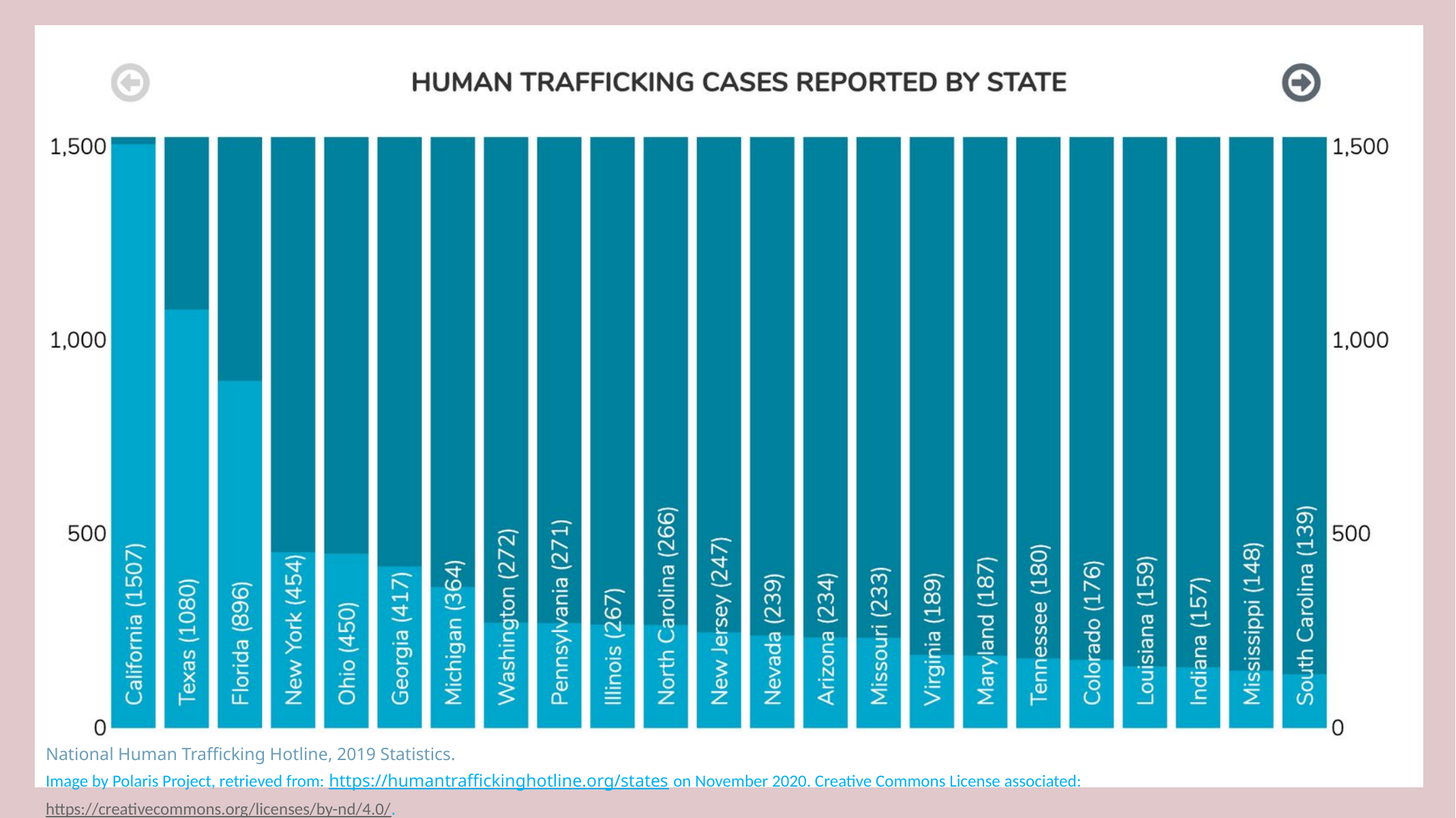

National Human Trafficking Hotline, 2019 Statistics.
Image by Polaris Project, retrieved from: https://humantraffickinghotline.org/states on November 2020. Creative Commons License associated: https://creativecommons.org/licenses/by-nd/4.0/.

## Slide 22
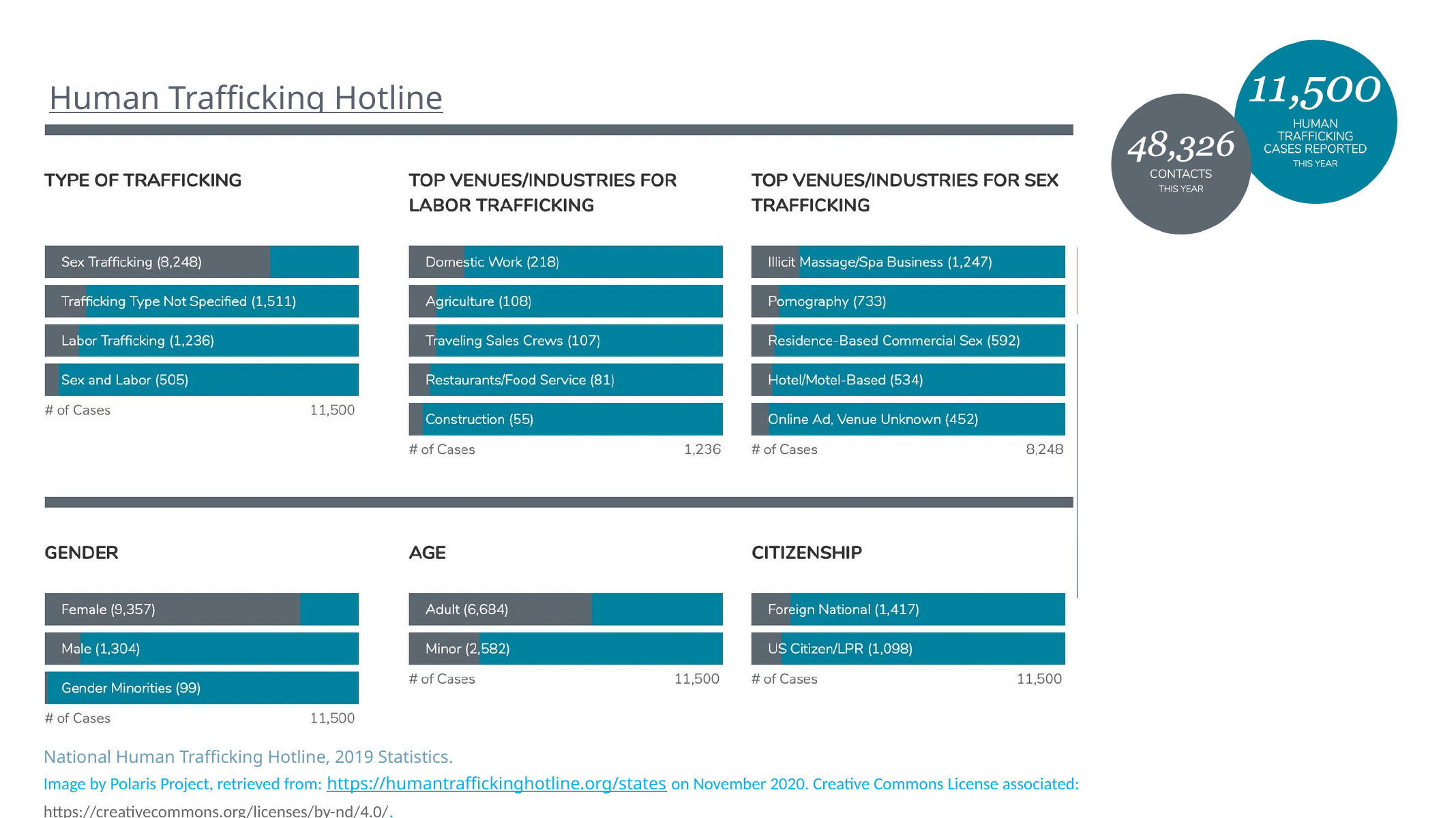

Human Trafficking Hotline
National Human Trafficking Hotline, 2019 Statistics.
Image by Polaris Project, retrieved from: https://humantraffickinghotline.org/states on November 2020. Creative Commons License associated: https://creativecommons.org/licenses/by-nd/4.0/.

## Slide 23
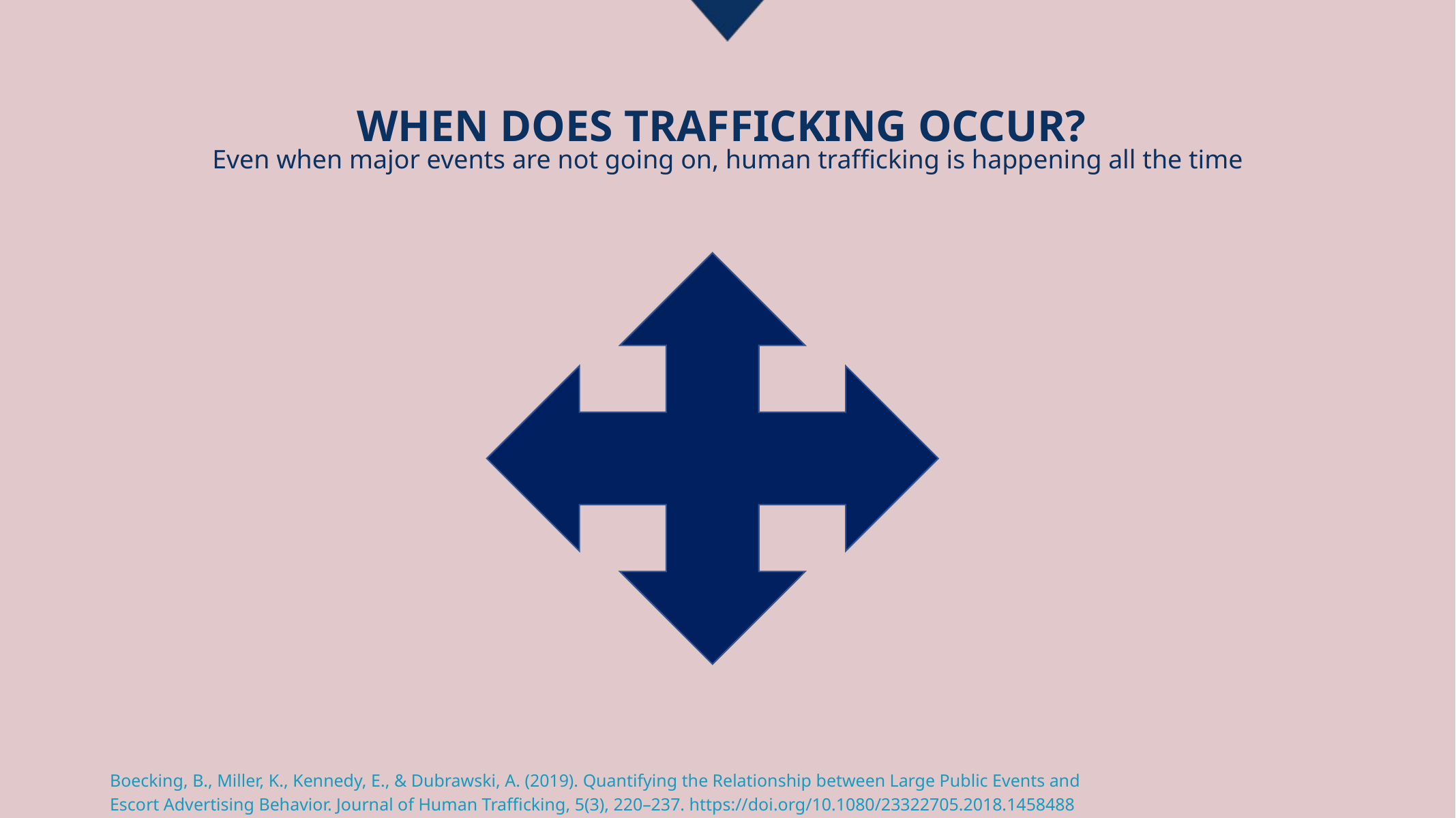

WHEN DOES TRAFFICKING OCCUR?
Even when major events are not going on, human trafficking is happening all the time
Boecking, B., Miller, K., Kennedy, E., & Dubrawski, A. (2019). Quantifying the Relationship between Large Public Events and Escort Advertising Behavior. Journal of Human Trafficking, 5(3), 220–237. https://doi.org/10.1080/23322705.2018.1458488

## Slide 24
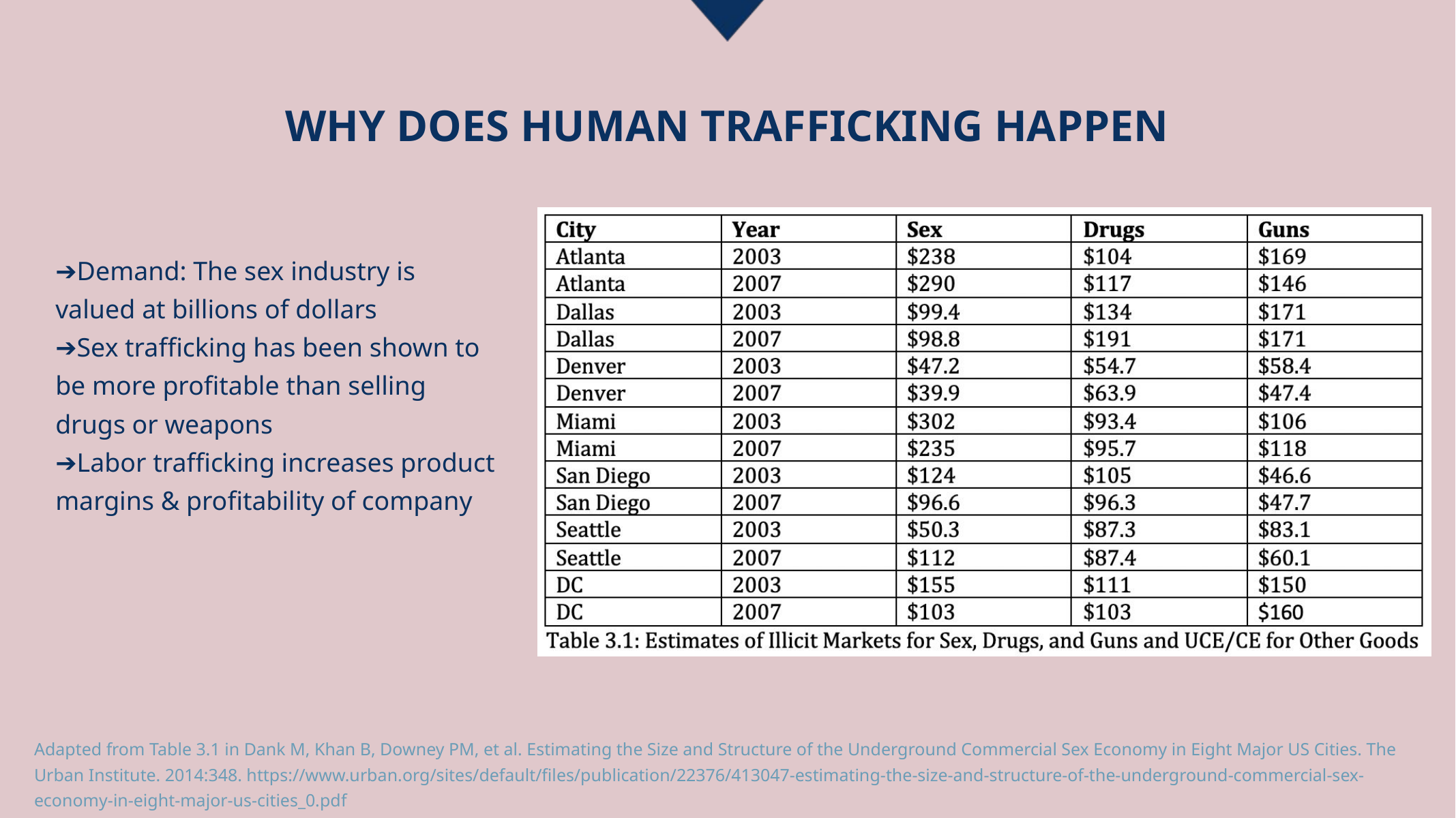

WHY DOES HUMAN TRAFFICKING HAPPEN
➔Demand: The sex industry is valued at billions of dollars
➔Sex trafficking has been shown to be more profitable than selling drugs or weapons
➔Labor trafficking increases product margins & profitability of company
Adapted from Table 3.1 in Dank M, Khan B, Downey PM, et al. Estimating the Size and Structure of the Underground Commercial Sex Economy in Eight Major US Cities. The Urban Institute. 2014:348. https://www.urban.org/sites/default/files/publication/22376/413047-estimating-the-size-and-structure-of-the-underground-commercial-sex-economy-in-eight-major-us-cities_0.pdf

## Slide 25
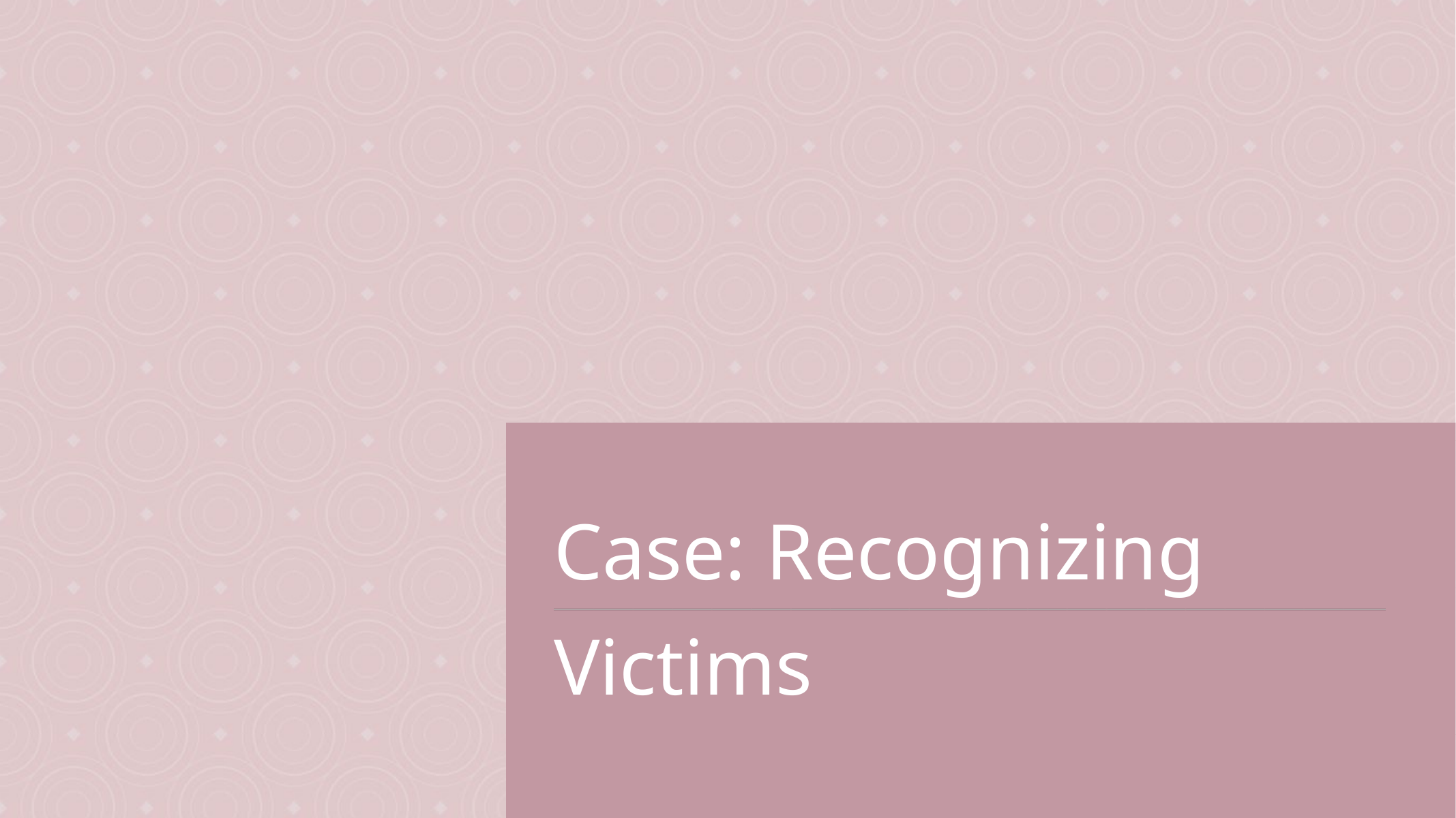

Case: Recognizing Victims

## Slide 26
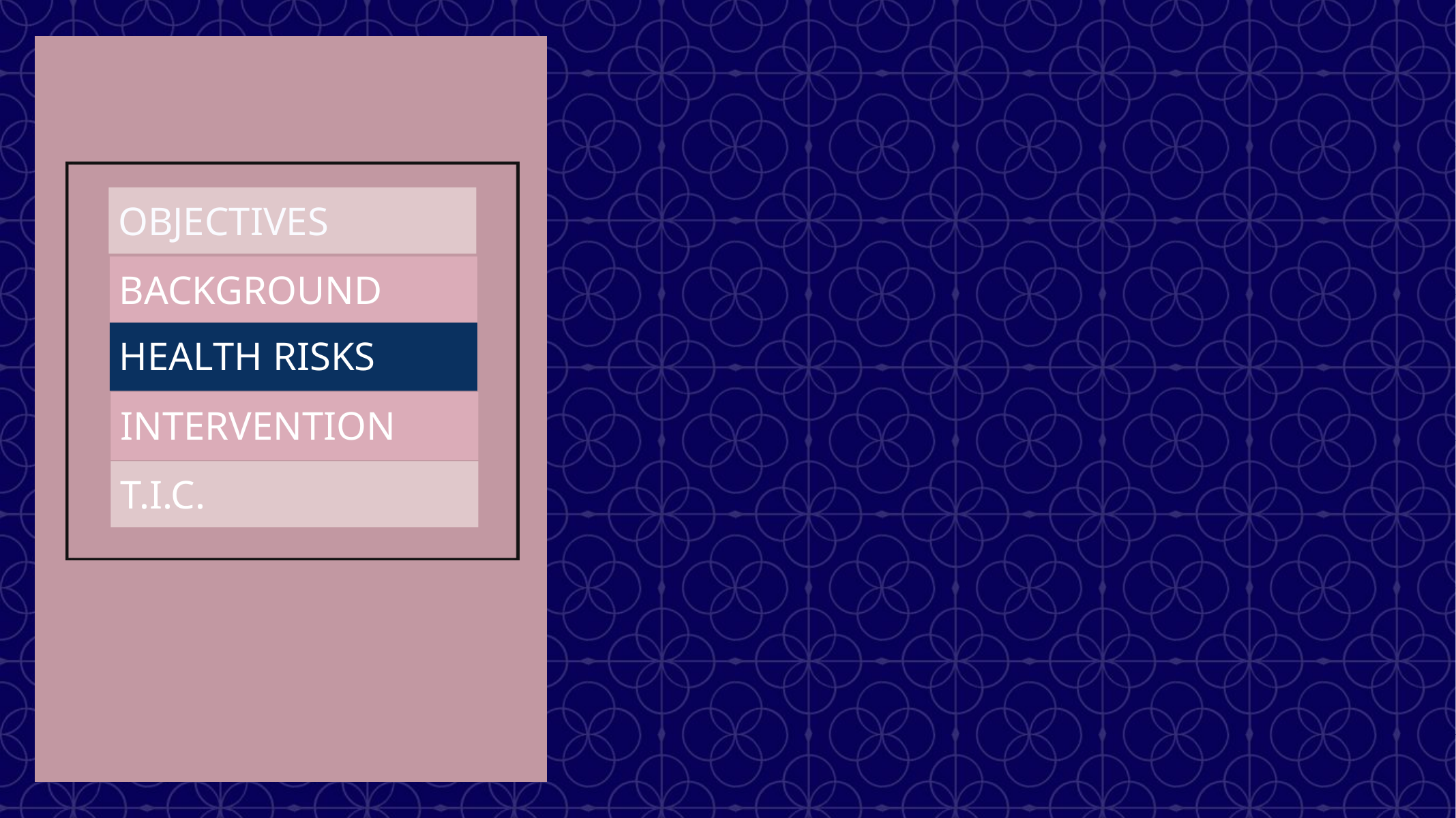

OBJECTIVES
BACKGROUND
HEALTH RISKS
INTERVENTION
T.I.C.

## Slide 27
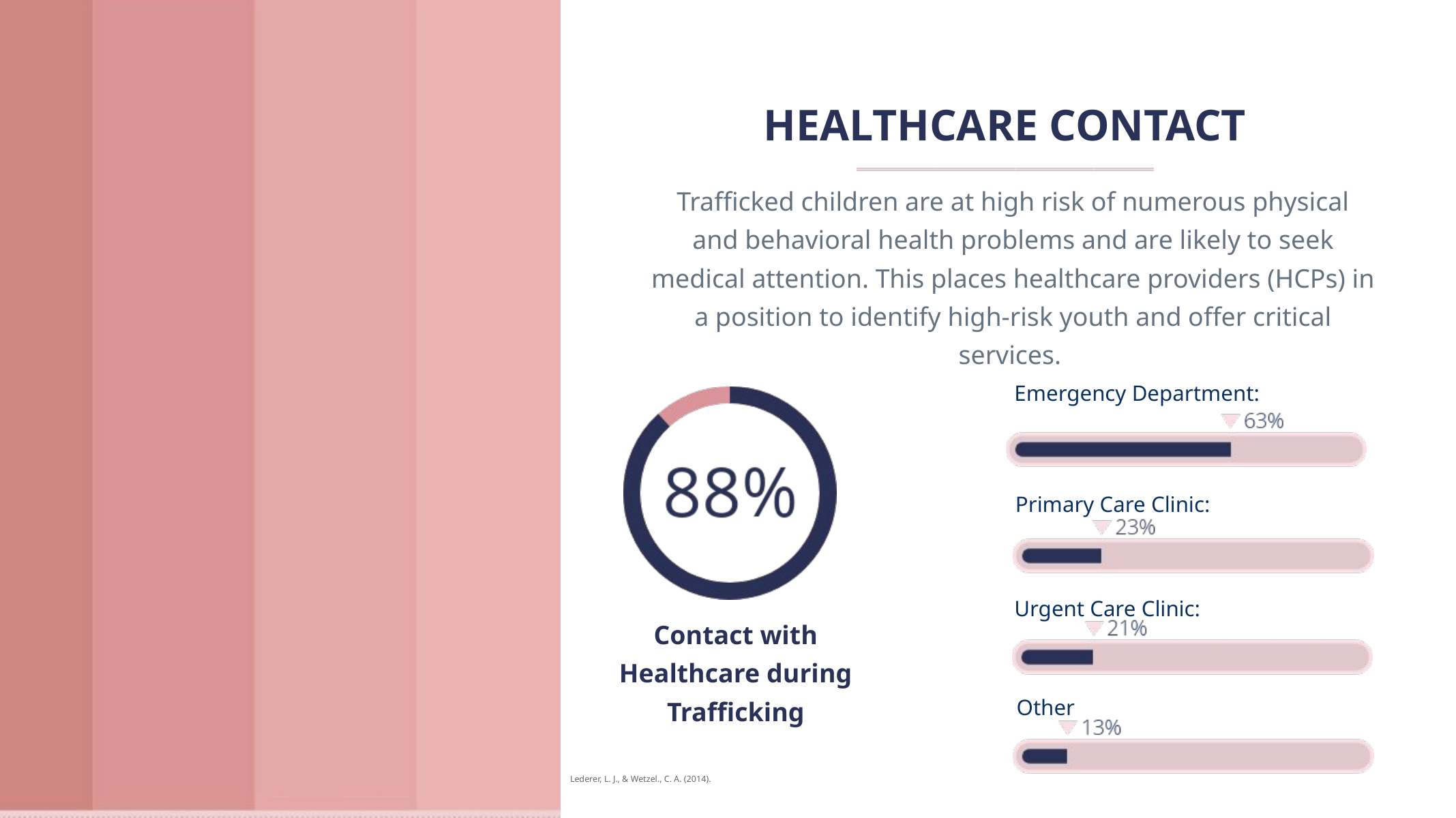

HEALTHCARE CONTACT
Trafficked children are at high risk of numerous physical and behavioral health problems and are likely to seek medical attention. This places healthcare providers (HCPs) in a position to identify high-risk youth and offer critical services.
Emergency Department:
Primary Care Clinic:
Urgent Care Clinic:
Contact with Healthcare during Trafficking
Other
 Lederer, L. J., & Wetzel., C. A. (2014).

## Slide 28
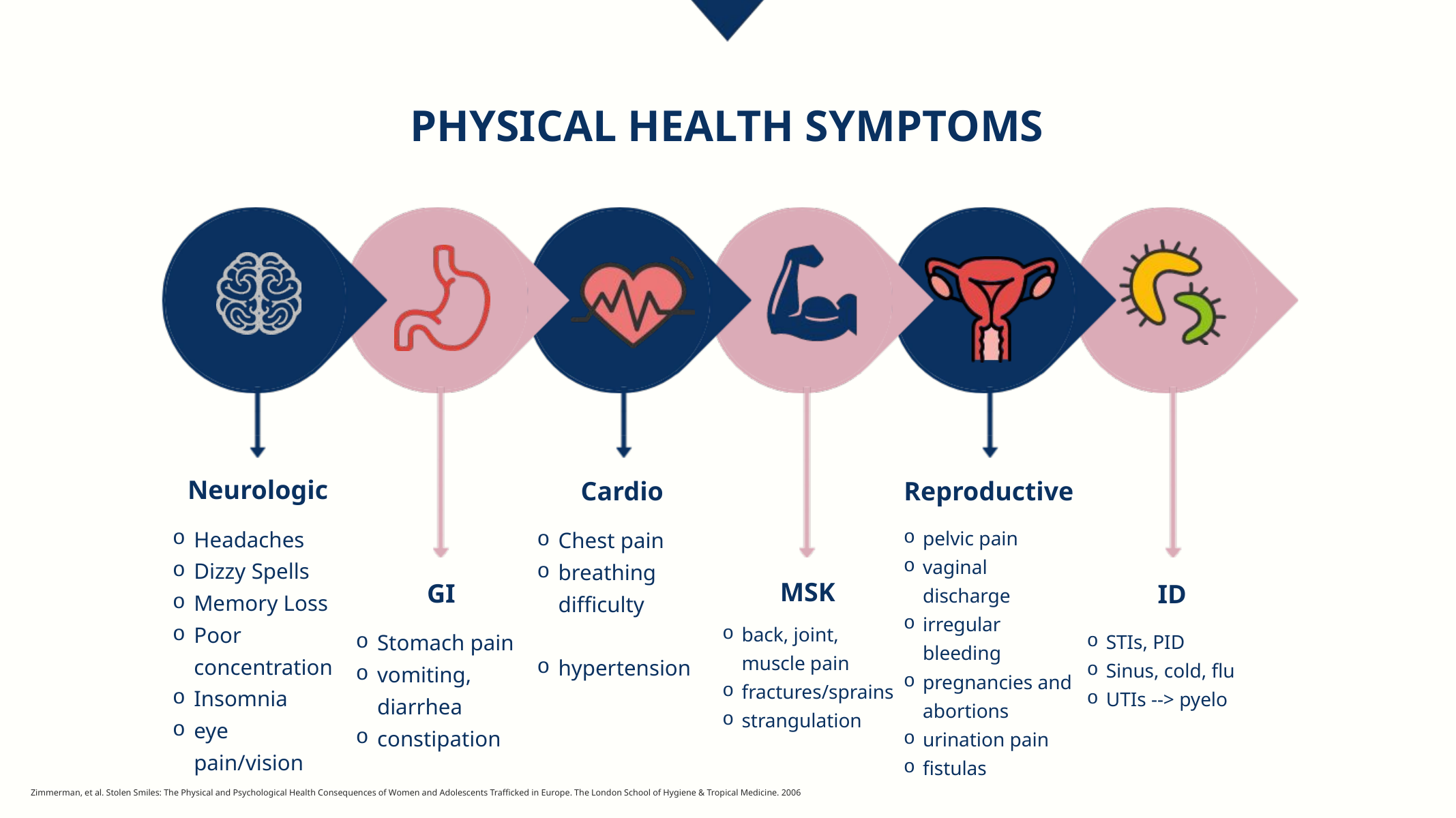

PHYSICAL HEALTH SYMPTOMS
Neurologic
Cardio
Reproductive
Headaches
Dizzy Spells
Memory Loss
Poor concentration
Insomnia
eye pain/vision
Chest pain
breathing difficulty
hypertension
pelvic pain
vaginal discharge
irregular bleeding
pregnancies and abortions
urination pain
fistulas
MSK
GI
ID
back, joint, muscle pain
fractures/sprains
strangulation
Stomach pain
vomiting, diarrhea
constipation
STIs, PID
Sinus, cold, flu
UTIs --> pyelo
Zimmerman, et al. Stolen Smiles: The Physical and Psychological Health Consequences of Women and Adolescents Trafficked in Europe. The London School of Hygiene & Tropical Medicine. 2006

## Slide 29
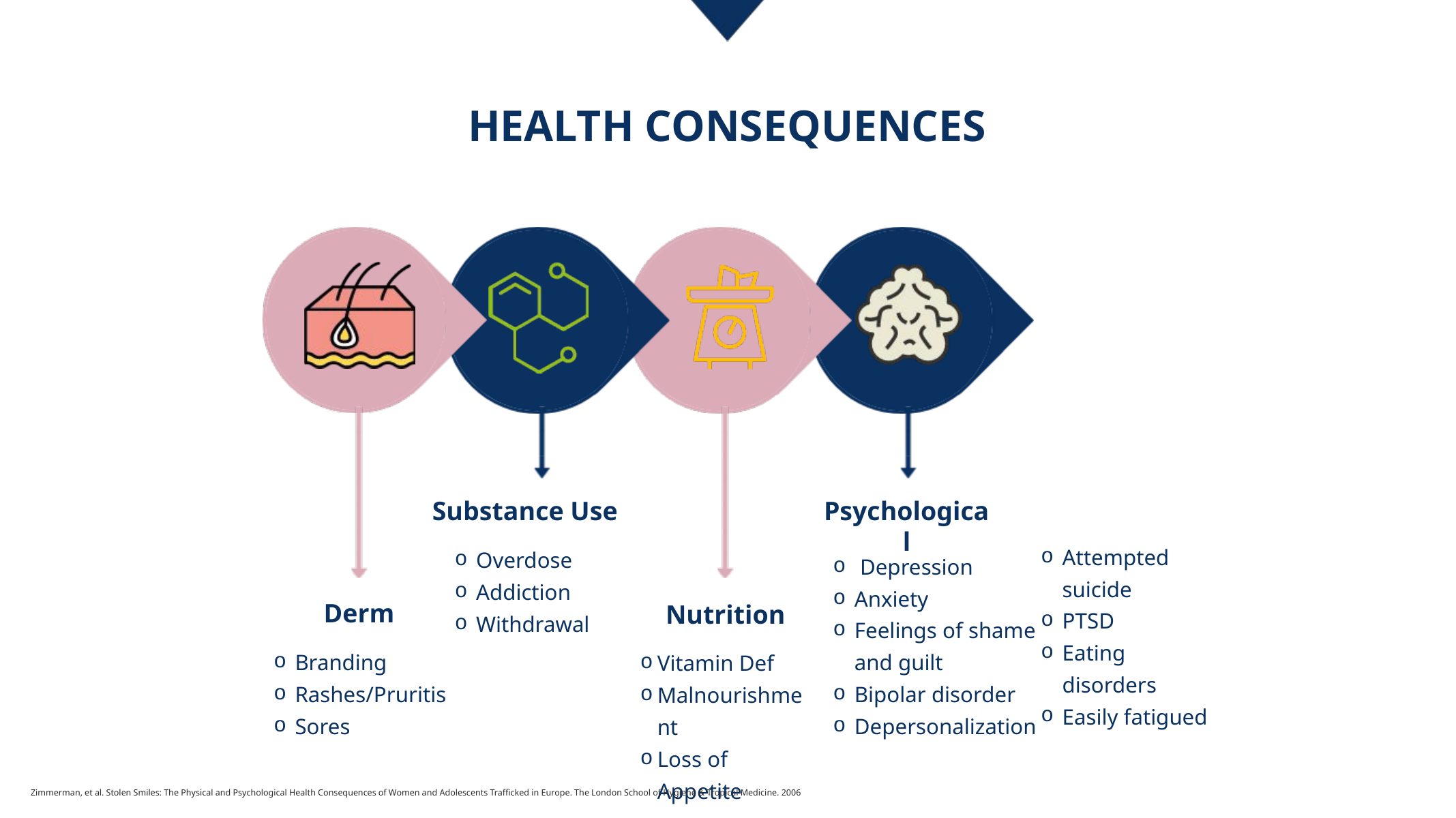

HEALTH CONSEQUENCES
Substance Use
Psychological
Attempted suicide
PTSD
Eating disorders
Easily fatigued
Overdose
Addiction
Withdrawal
 Depression
Anxiety
Feelings of shame and guilt
Bipolar disorder
Depersonalization
Derm
Nutrition
Branding
Rashes/Pruritis
Sores
Vitamin Def
Malnourishment
Loss of Appetite
Zimmerman, et al. Stolen Smiles: The Physical and Psychological Health Consequences of Women and Adolescents Trafficked in Europe. The London School of Hygiene & Tropical Medicine. 2006

## Slide 30
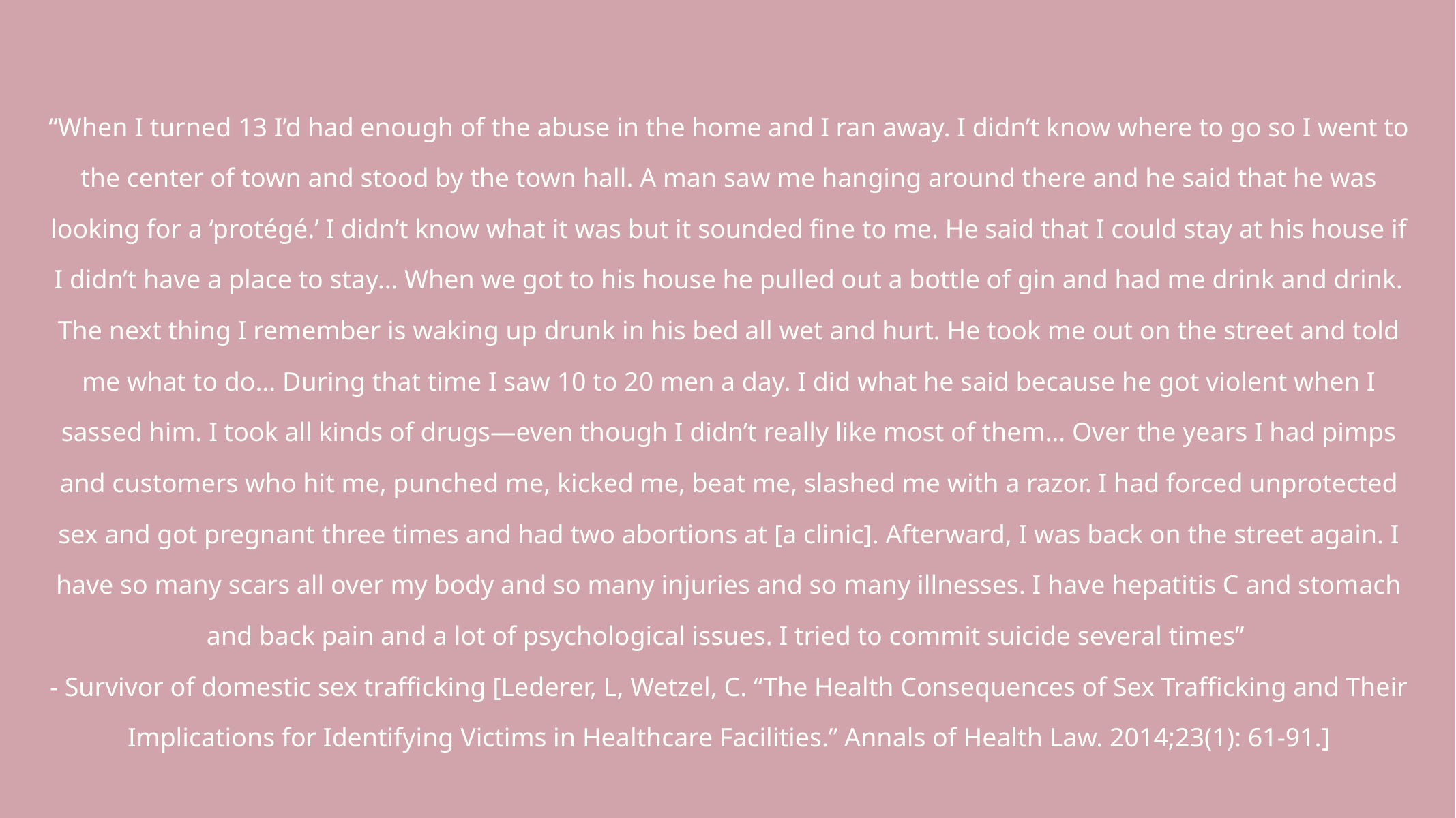

“When I turned 13 I’d had enough of the abuse in the home and I ran away. I didn’t know where to go so I went to the center of town and stood by the town hall. A man saw me hanging around there and he said that he was looking for a ‘protégé.’ I didn’t know what it was but it sounded fine to me. He said that I could stay at his house if I didn’t have a place to stay… When we got to his house he pulled out a bottle of gin and had me drink and drink. The next thing I remember is waking up drunk in his bed all wet and hurt. He took me out on the street and told me what to do… During that time I saw 10 to 20 men a day. I did what he said because he got violent when I sassed him. I took all kinds of drugs—even though I didn’t really like most of them… Over the years I had pimps and customers who hit me, punched me, kicked me, beat me, slashed me with a razor. I had forced unprotected sex and got pregnant three times and had two abortions at [a clinic]. Afterward, I was back on the street again. I have so many scars all over my body and so many injuries and so many illnesses. I have hepatitis C and stomach and back pain and a lot of psychological issues. I tried to commit suicide several times”
- Survivor of domestic sex trafficking [Lederer, L, Wetzel, C. “The Health Consequences of Sex Trafficking and Their Implications for Identifying Victims in Healthcare Facilities.” Annals of Health Law. 2014;23(1): 61-91.]

## Slide 31
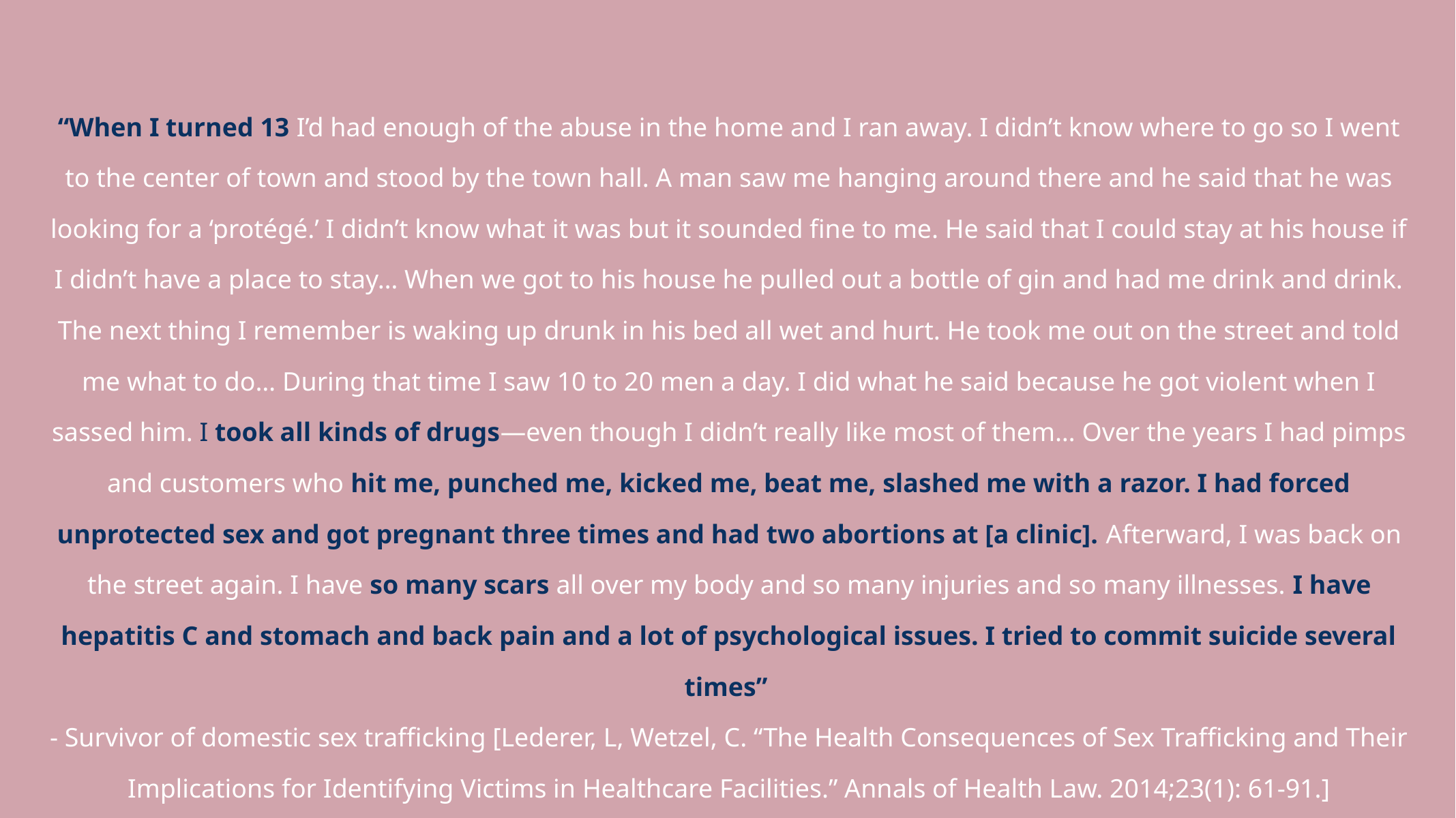

“When I turned 13 I’d had enough of the abuse in the home and I ran away. I didn’t know where to go so I went to the center of town and stood by the town hall. A man saw me hanging around there and he said that he was looking for a ‘protégé.’ I didn’t know what it was but it sounded fine to me. He said that I could stay at his house if I didn’t have a place to stay… When we got to his house he pulled out a bottle of gin and had me drink and drink. The next thing I remember is waking up drunk in his bed all wet and hurt. He took me out on the street and told me what to do… During that time I saw 10 to 20 men a day. I did what he said because he got violent when I sassed him. I took all kinds of drugs—even though I didn’t really like most of them… Over the years I had pimps and customers who hit me, punched me, kicked me, beat me, slashed me with a razor. I had forced unprotected sex and got pregnant three times and had two abortions at [a clinic]. Afterward, I was back on the street again. I have so many scars all over my body and so many injuries and so many illnesses. I have hepatitis C and stomach and back pain and a lot of psychological issues. I tried to commit suicide several times”
- Survivor of domestic sex trafficking [Lederer, L, Wetzel, C. “The Health Consequences of Sex Trafficking and Their Implications for Identifying Victims in Healthcare Facilities.” Annals of Health Law. 2014;23(1): 61-91.]

## Slide 32
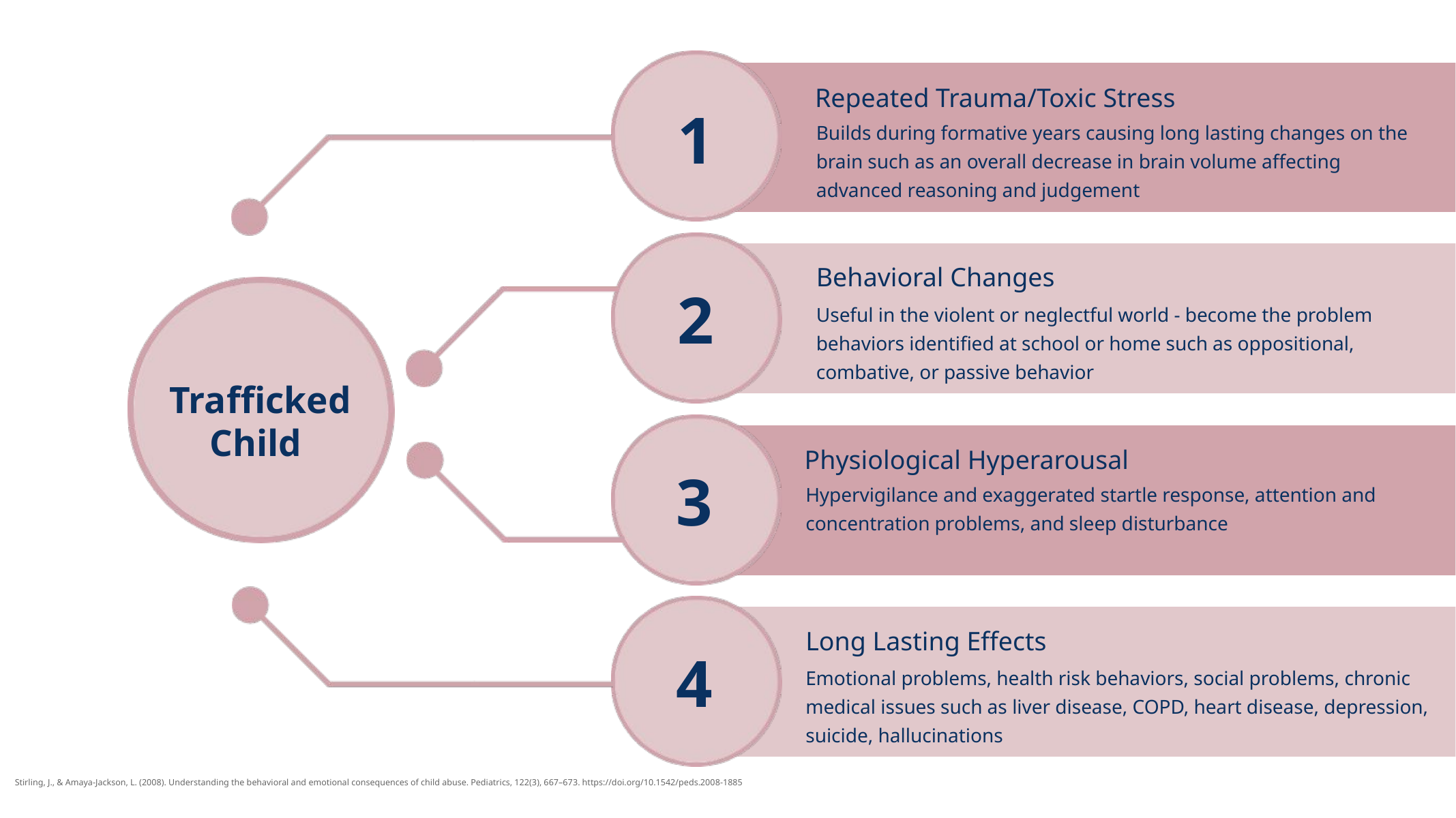

Repeated Trauma/Toxic Stress
1
Builds during formative years causing long lasting changes on the brain such as an overall decrease in brain volume affecting advanced reasoning and judgement
Behavioral Changes
2
Useful in the violent or neglectful world - become the problem behaviors identified at school or home such as oppositional, combative, or passive behavior
Trafficked Child
Physiological Hyperarousal
3
Hypervigilance and exaggerated startle response, attention and concentration problems, and sleep disturbance
Long Lasting Effects
4
Emotional problems, health risk behaviors, social problems, chronic medical issues such as liver disease, COPD, heart disease, depression, suicide, hallucinations
Stirling, J., & Amaya-Jackson, L. (2008). Understanding the behavioral and emotional consequences of child abuse. Pediatrics, 122(3), 667–673. https://doi.org/10.1542/peds.2008-1885

## Slide 33
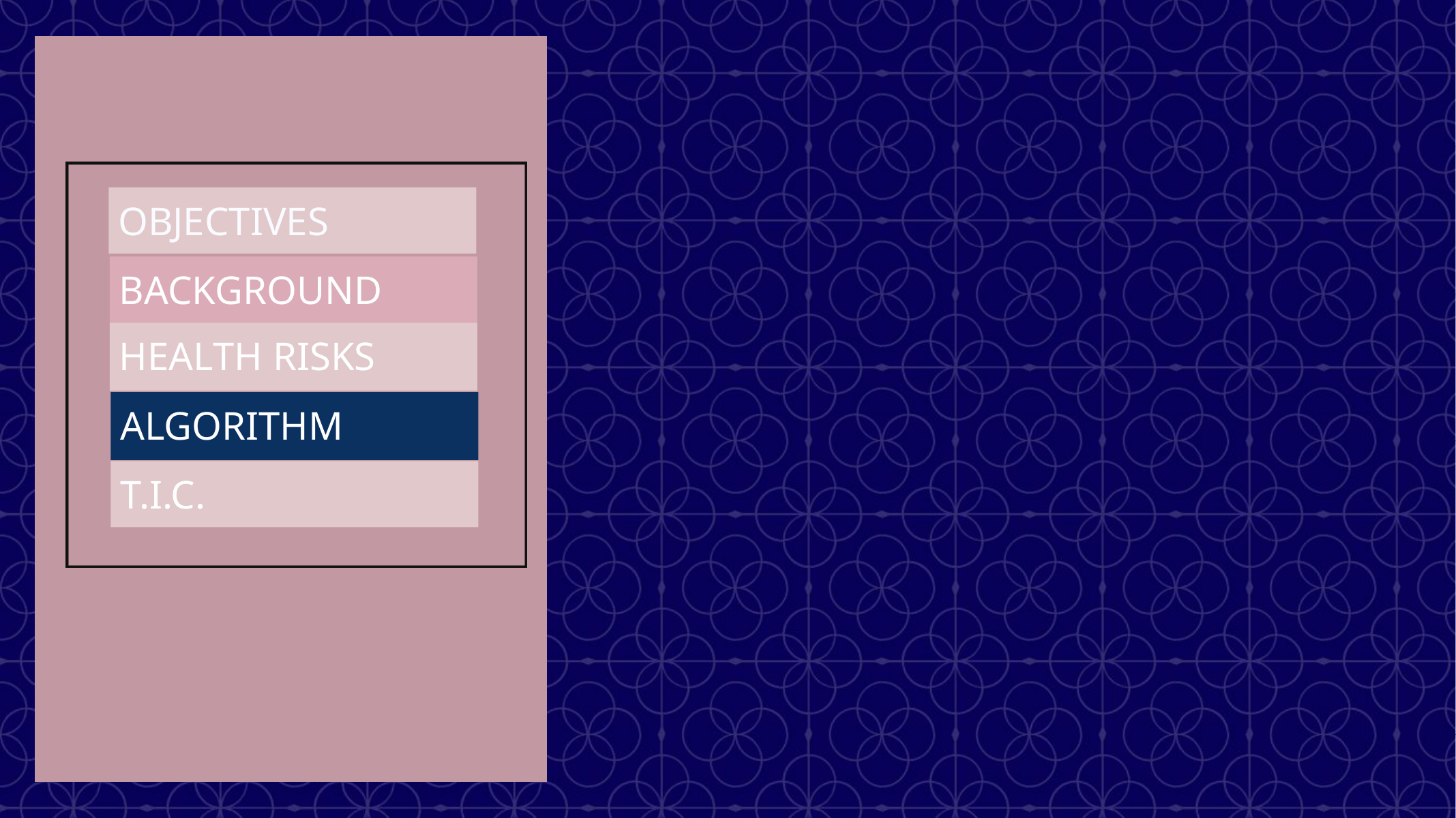

OBJECTIVES
BACKGROUND
HEALTH RISKS
ALGORITHM
T.I.C.

## Slide 34
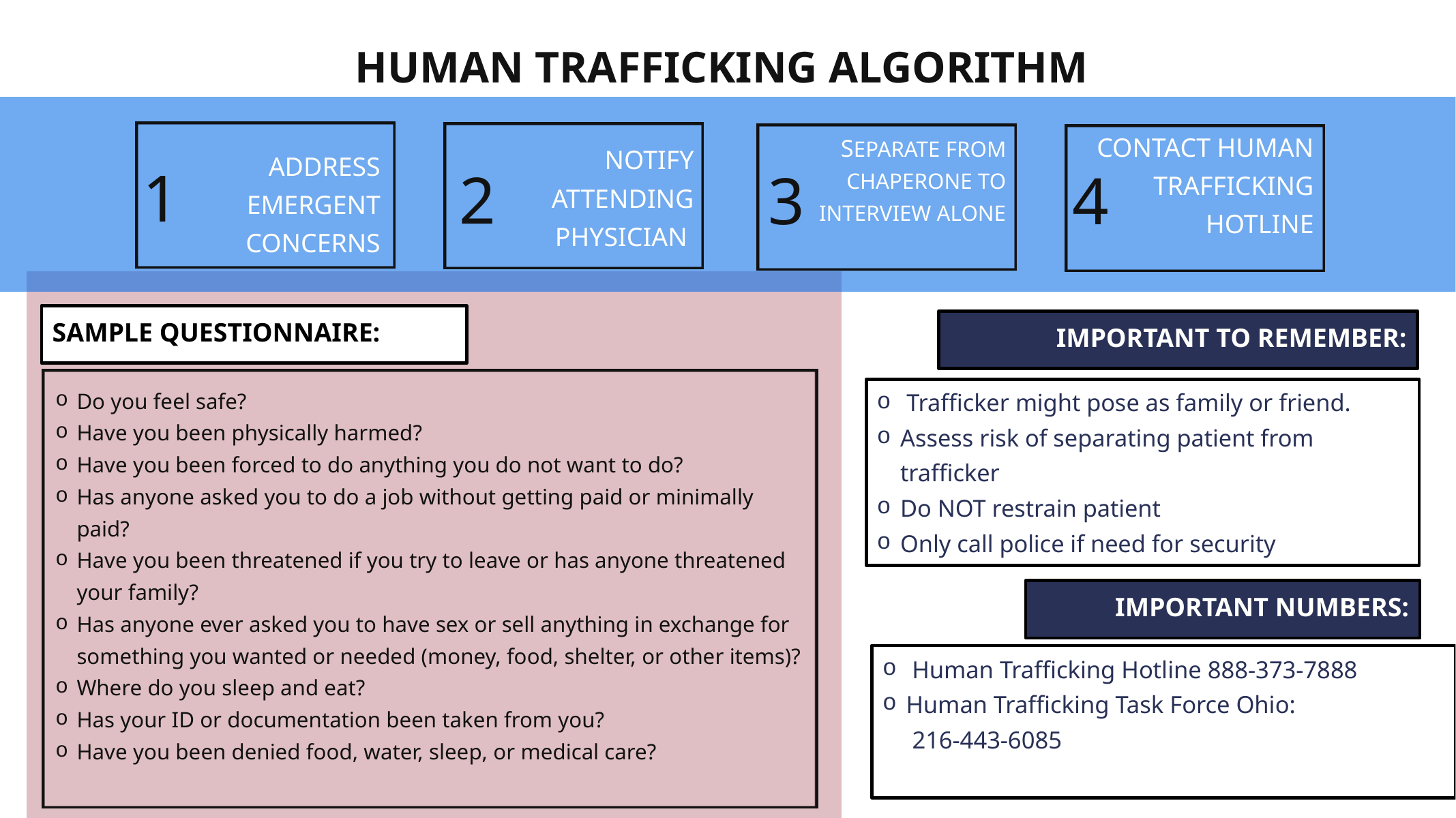

HUMAN TRAFFICKING ALGORITHM
CONTACT HUMAN TRAFFICKING HOTLINE
SEPARATE FROM CHAPERONE TO INTERVIEW ALONE
NOTIFY ATTENDING PHYSICIAN
ADDRESS EMERGENT CONCERNS
1
2
3
4
SAMPLE QUESTIONNAIRE:
IMPORTANT TO REMEMBER:
Do you feel safe?
Have you been physically harmed?
Have you been forced to do anything you do not want to do?
Has anyone asked you to do a job without getting paid or minimally paid?
Have you been threatened if you try to leave or has anyone threatened your family?
Has anyone ever asked you to have sex or sell anything in exchange for something you wanted or needed (money, food, shelter, or other items)?
Where do you sleep and eat?
Has your ID or documentation been taken from you?
Have you been denied food, water, sleep, or medical care?
 Trafficker might pose as family or friend.
Assess risk of separating patient from trafficker
Do NOT restrain patient
Only call police if need for security
IMPORTANT NUMBERS:
 Human Trafficking Hotline 888-373-7888
Human Trafficking Task Force Ohio:
 216-443-6085

## Slide 35
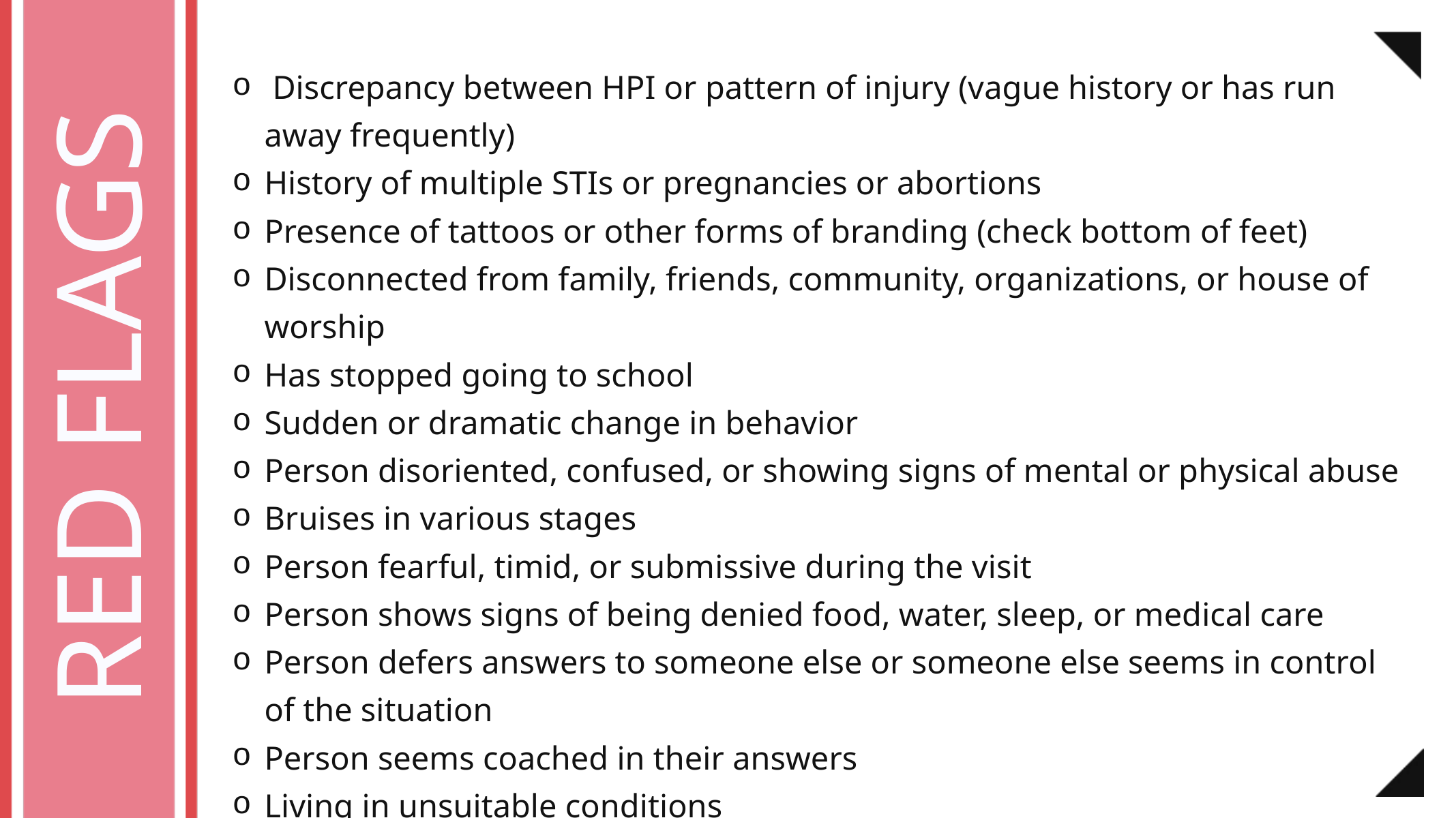

Discrepancy between HPI or pattern of injury (vague history or has run away frequently)
History of multiple STIs or pregnancies or abortions
Presence of tattoos or other forms of branding (check bottom of feet)
Disconnected from family, friends, community, organizations, or house of worship
Has stopped going to school
Sudden or dramatic change in behavior
Person disoriented, confused, or showing signs of mental or physical abuse
Bruises in various stages
Person fearful, timid, or submissive during the visit
Person shows signs of being denied food, water, sleep, or medical care
Person defers answers to someone else or someone else seems in control of the situation
Person seems coached in their answers
Living in unsuitable conditions
Lack of personal possessions
RED FLAGS

## Slide 36
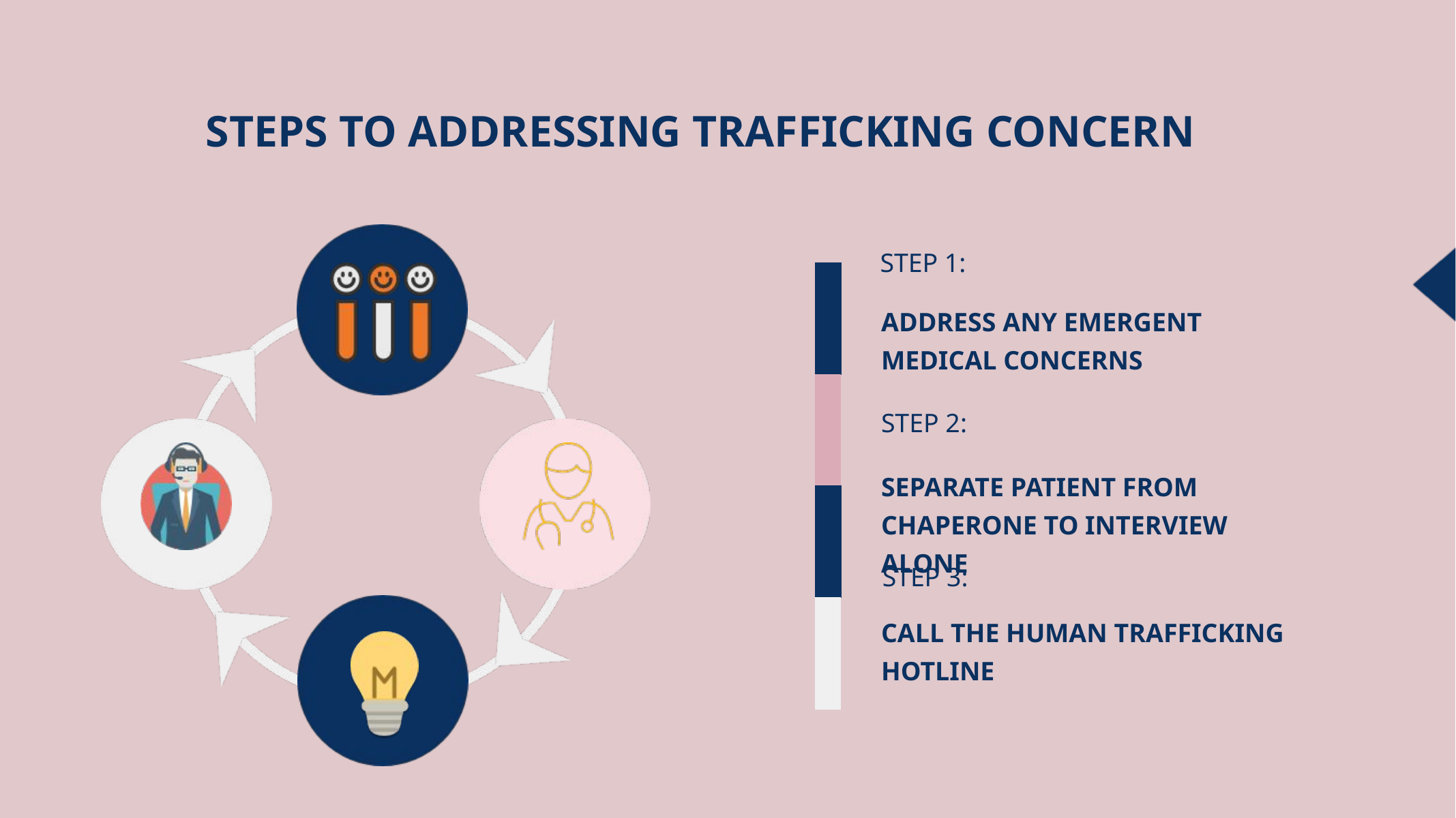

STEPS TO ADDRESSING TRAFFICKING CONCERN
STEP 1:
ADDRESS ANY EMERGENT MEDICAL CONCERNS
STEP 2:
SEPARATE PATIENT FROM CHAPERONE TO INTERVIEW ALONE
STEP 3:
CALL THE HUMAN TRAFFICKING HOTLINE

## Slide 37
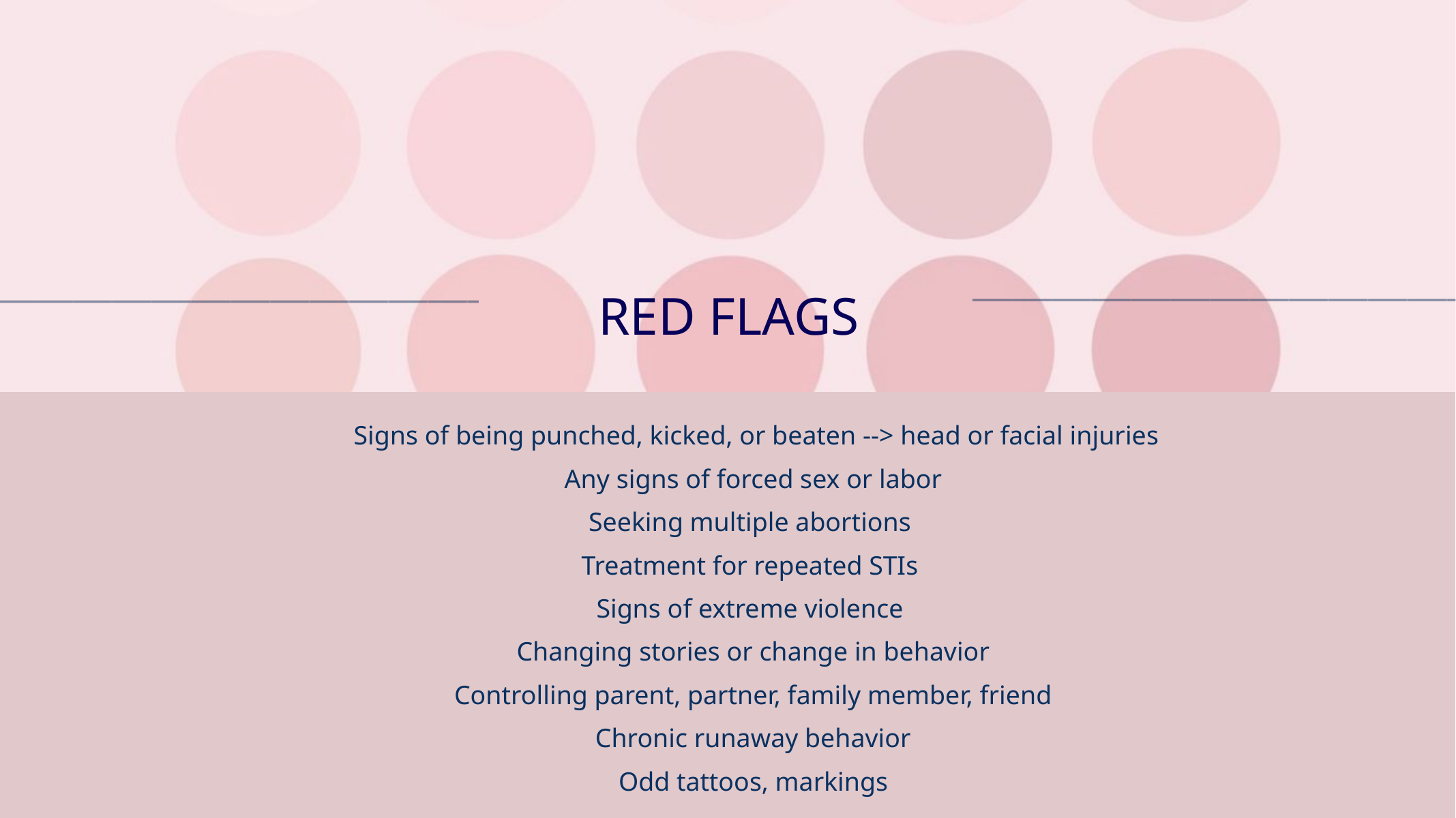

RED FLAGS
 Signs of being punched, kicked, or beaten --> head or facial injuries
Any signs of forced sex or labor
Seeking multiple abortions
Treatment for repeated STIs
Signs of extreme violence
Changing stories or change in behavior
Controlling parent, partner, family member, friend
Chronic runaway behavior
Odd tattoos, markings

## Slide 38
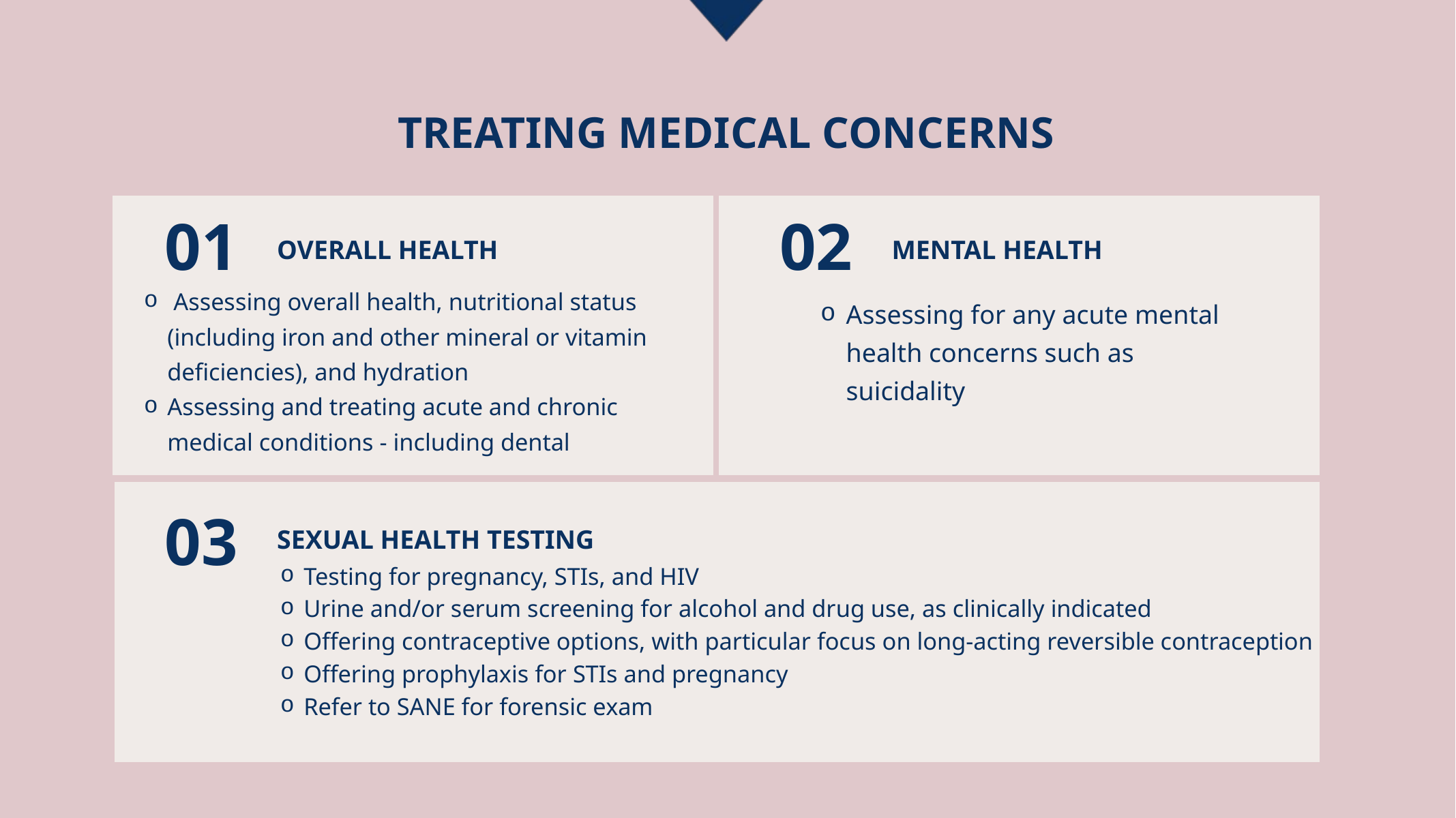

TREATING MEDICAL CONCERNS
01
02
OVERALL HEALTH
MENTAL HEALTH
 Assessing overall health, nutritional status (including iron and other mineral or vitamin deficiencies), and hydration
Assessing and treating acute and chronic medical conditions - including dental
Assessing for any acute mental health concerns such as suicidality
03
SEXUAL HEALTH TESTING
Testing for pregnancy, STIs, and HIV
Urine and/or serum screening for alcohol and drug use, as clinically indicated
Offering contraceptive options, with particular focus on long-acting reversible contraception
Offering prophylaxis for STIs and pregnancy
Refer to SANE for forensic exam
45%Achievement 1
Subtitle Here
Lorem ipsum dolor sit amet, consectetur adipiscing elit.
45%
Subtitle Here
Lorem ipsum dolor sit amet, consectetur adipiscing elit.
50%
75%
Subtitle Here
75%
Lorem ipsum dolor sit amet, consectetur adipiscing elit.

## Slide 39
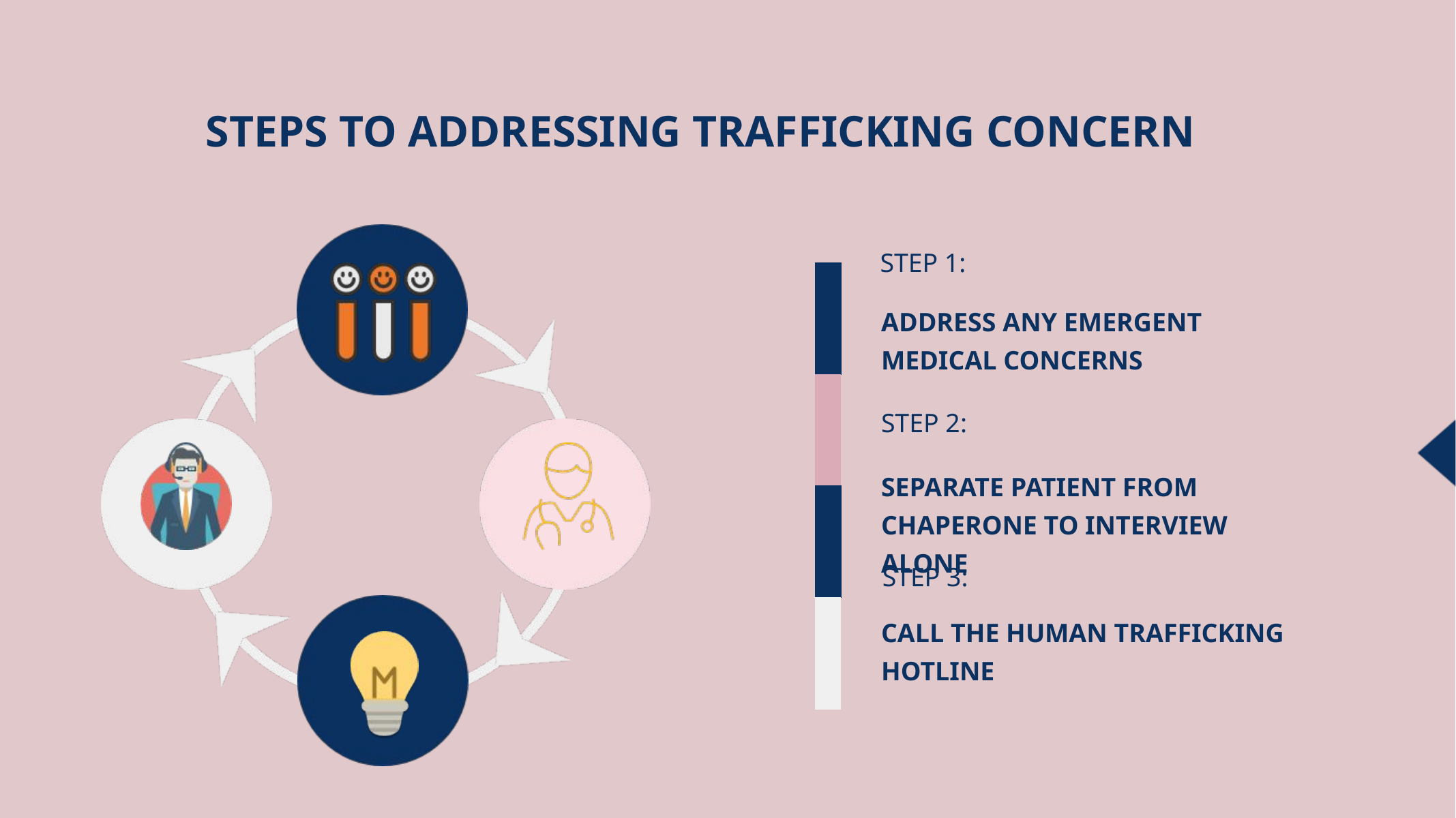

STEPS TO ADDRESSING TRAFFICKING CONCERN
STEP 1:
ADDRESS ANY EMERGENT MEDICAL CONCERNS
STEP 2:
SEPARATE PATIENT FROM CHAPERONE TO INTERVIEW ALONE
STEP 3:
CALL THE HUMAN TRAFFICKING HOTLINE

## Slide 40
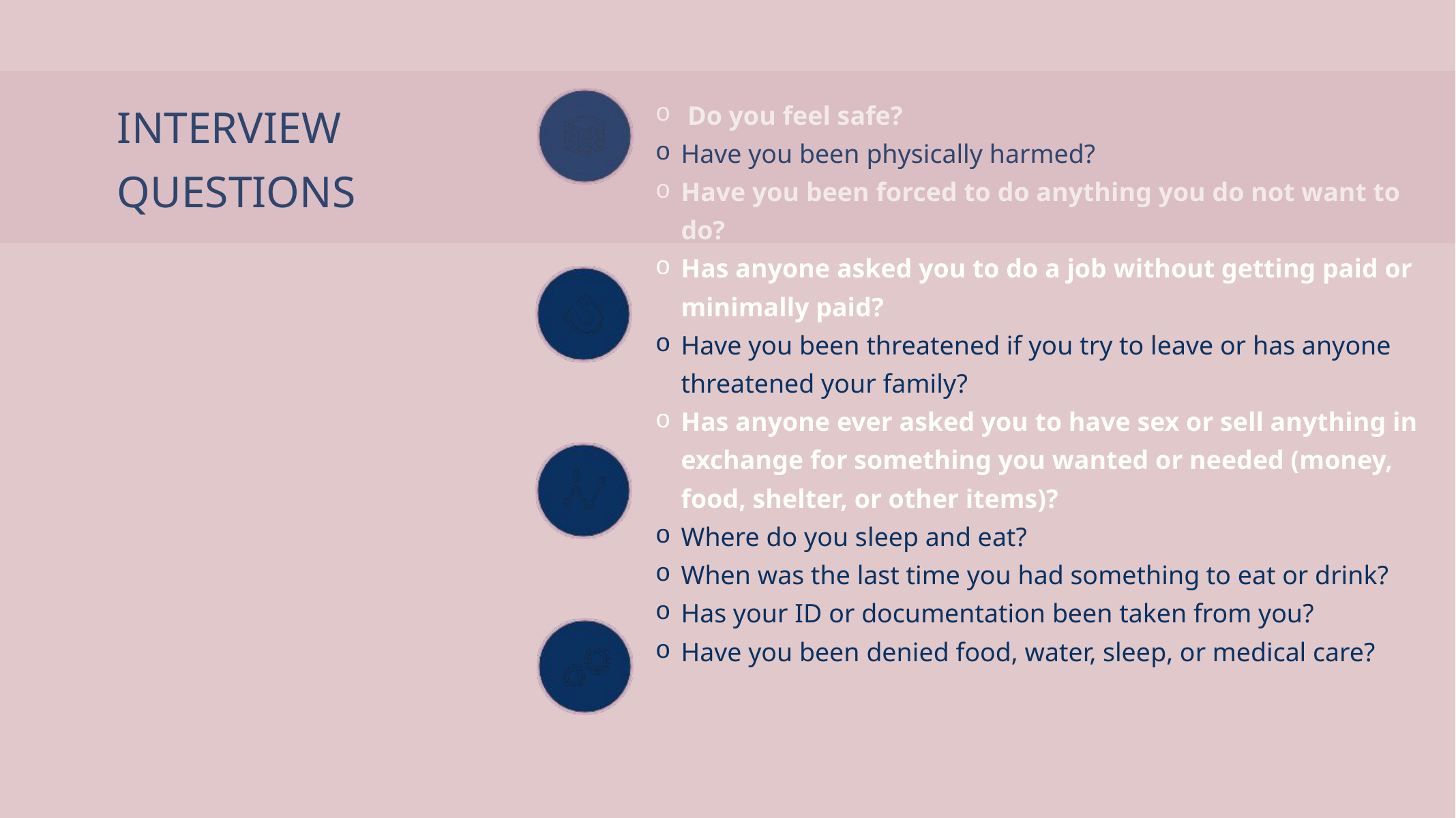

Do you feel safe?
Have you been physically harmed?
Have you been forced to do anything you do not want to do?
Has anyone asked you to do a job without getting paid or minimally paid?
Have you been threatened if you try to leave or has anyone threatened your family?
Has anyone ever asked you to have sex or sell anything in exchange for something you wanted or needed (money, food, shelter, or other items)?
Where do you sleep and eat?
When was the last time you had something to eat or drink?
Has your ID or documentation been taken from you?
Have you been denied food, water, sleep, or medical care?
INTERVIEW QUESTIONS

## Slide 41
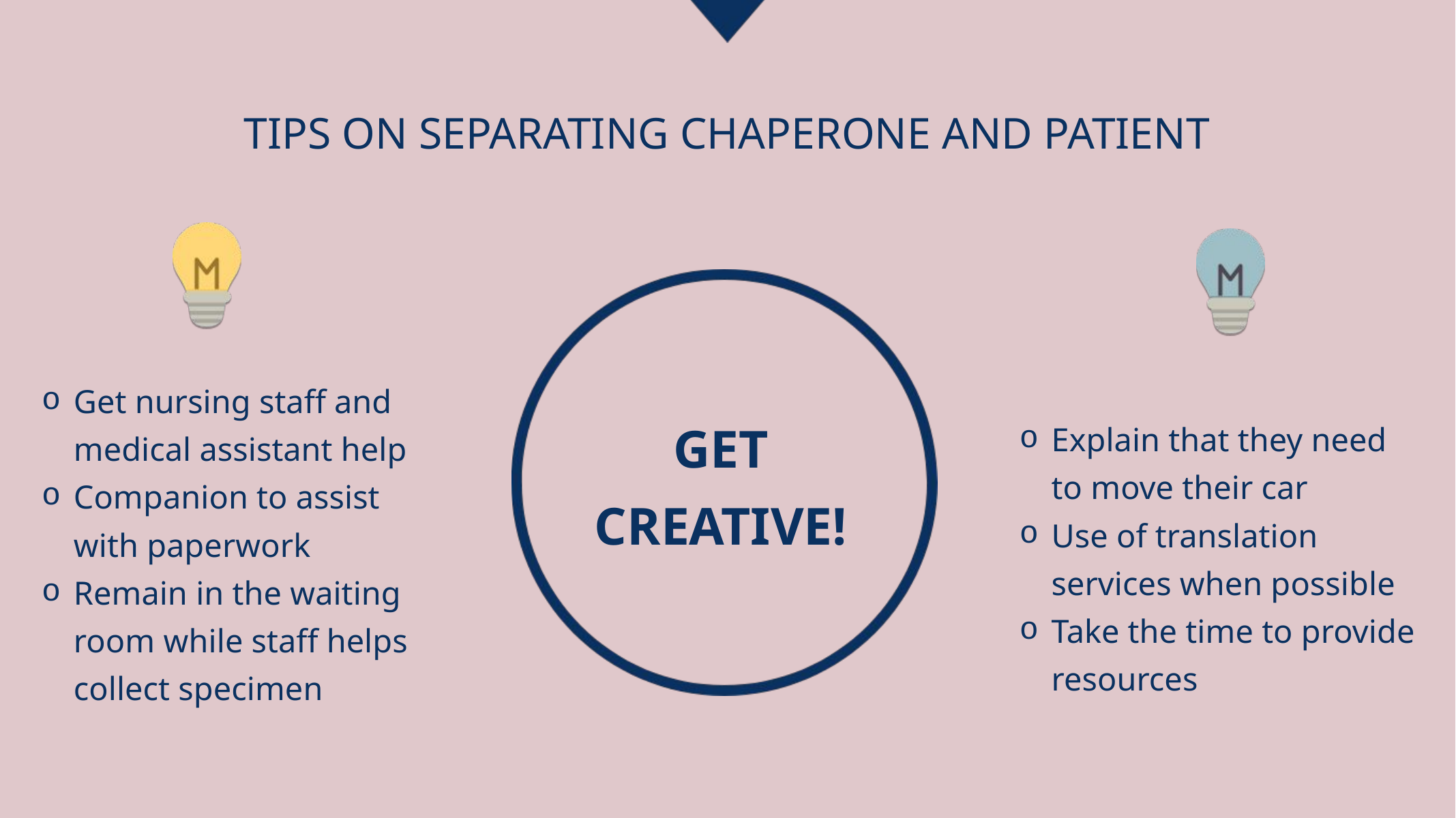

TIPS ON SEPARATING CHAPERONE AND PATIENT
Get nursing staff and medical assistant help
Companion to assist with paperwork
Remain in the waiting room while staff helps collect specimen
GET CREATIVE!
Explain that they need to move their car
Use of translation services when possible
Take the time to provide resources

## Slide 42
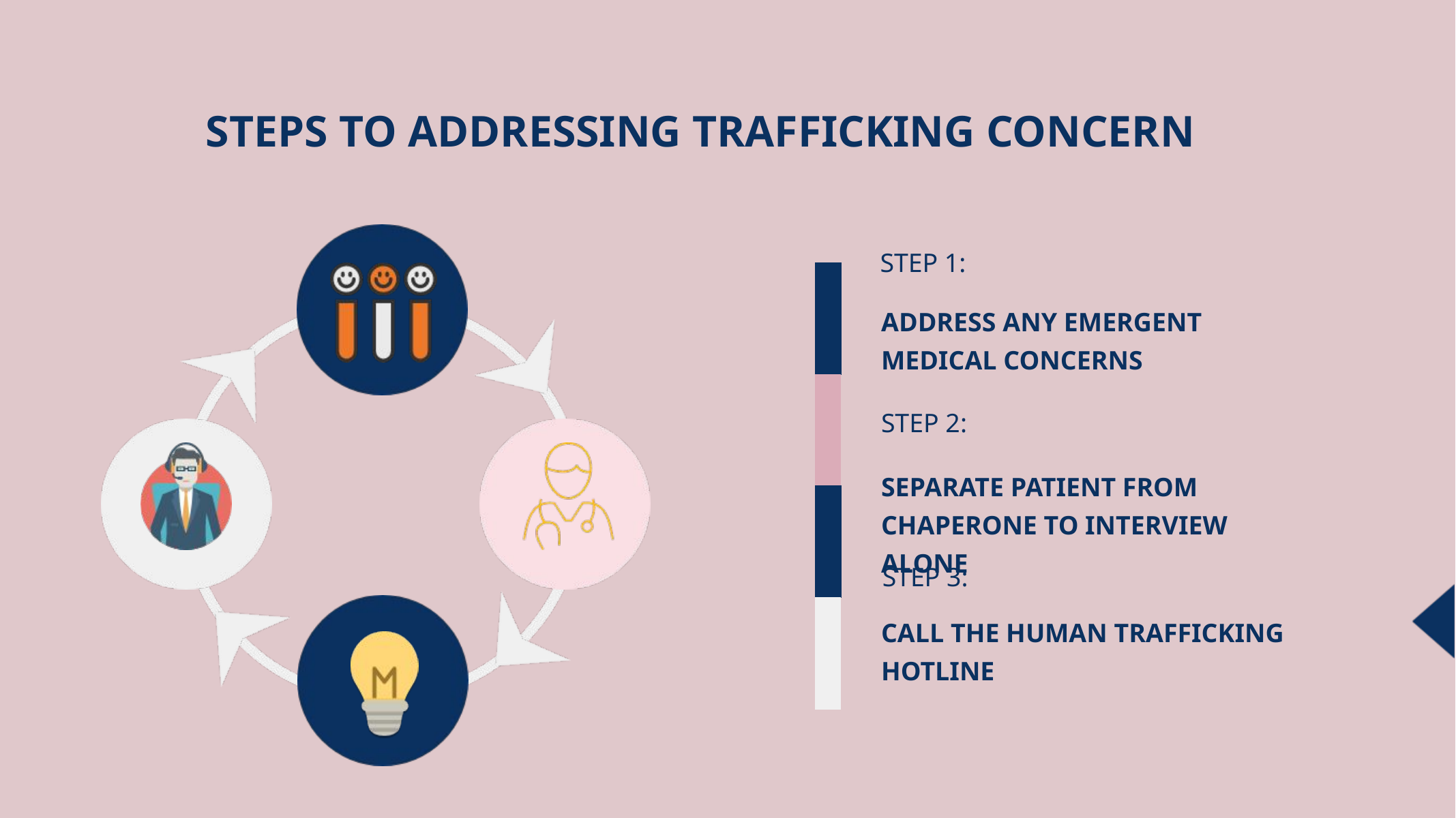

STEPS TO ADDRESSING TRAFFICKING CONCERN
STEP 1:
ADDRESS ANY EMERGENT MEDICAL CONCERNS
STEP 2:
SEPARATE PATIENT FROM CHAPERONE TO INTERVIEW ALONE
STEP 3:
CALL THE HUMAN TRAFFICKING HOTLINE

## Slide 43
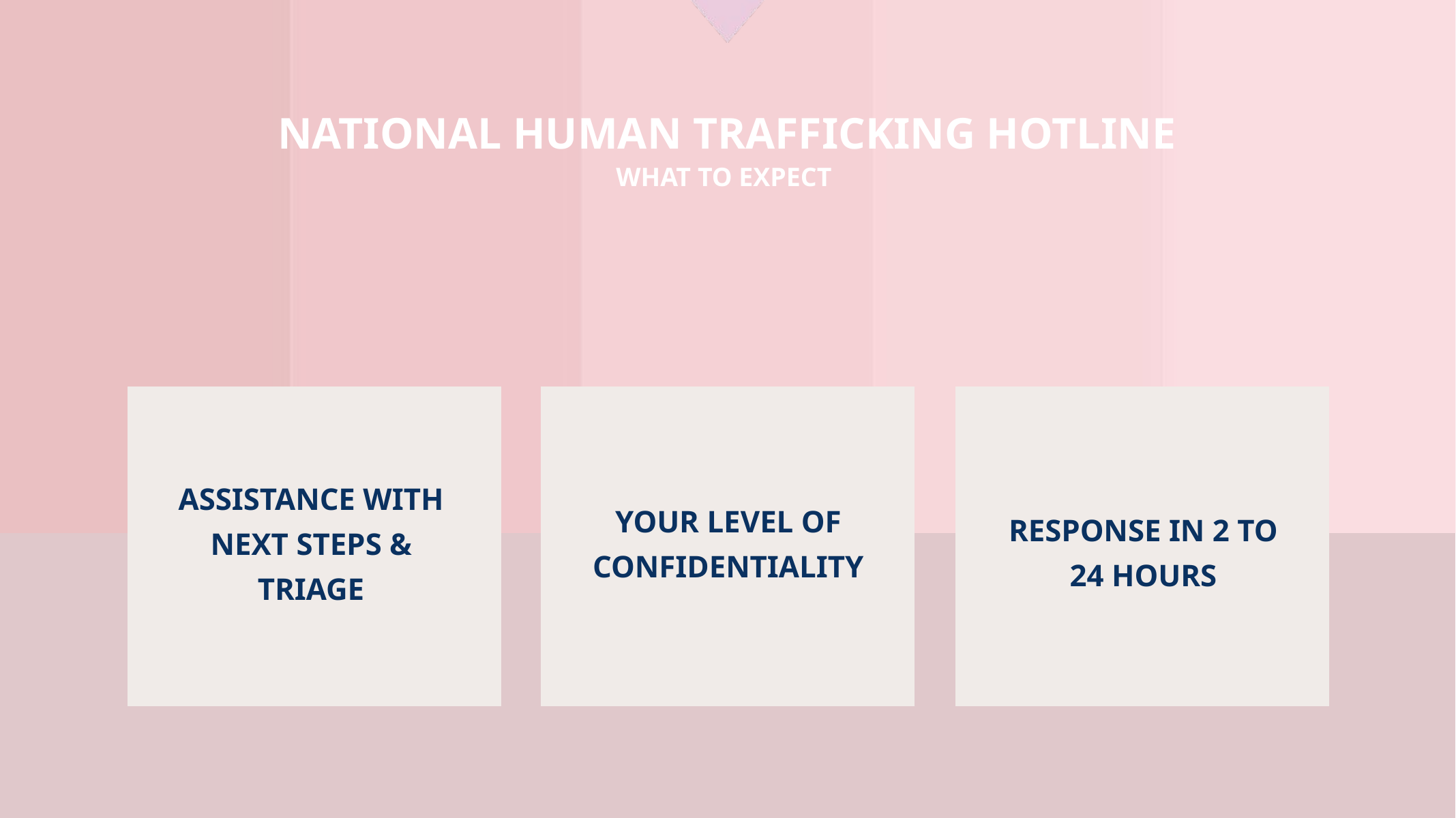

NATIONAL HUMAN TRAFFICKING HOTLINE
WHAT TO EXPECT
ASSISTANCE WITH NEXT STEPS & TRIAGE
YOUR LEVEL OF CONFIDENTIALITY
RESPONSE IN 2 TO 24 HOURS

## Slide 44
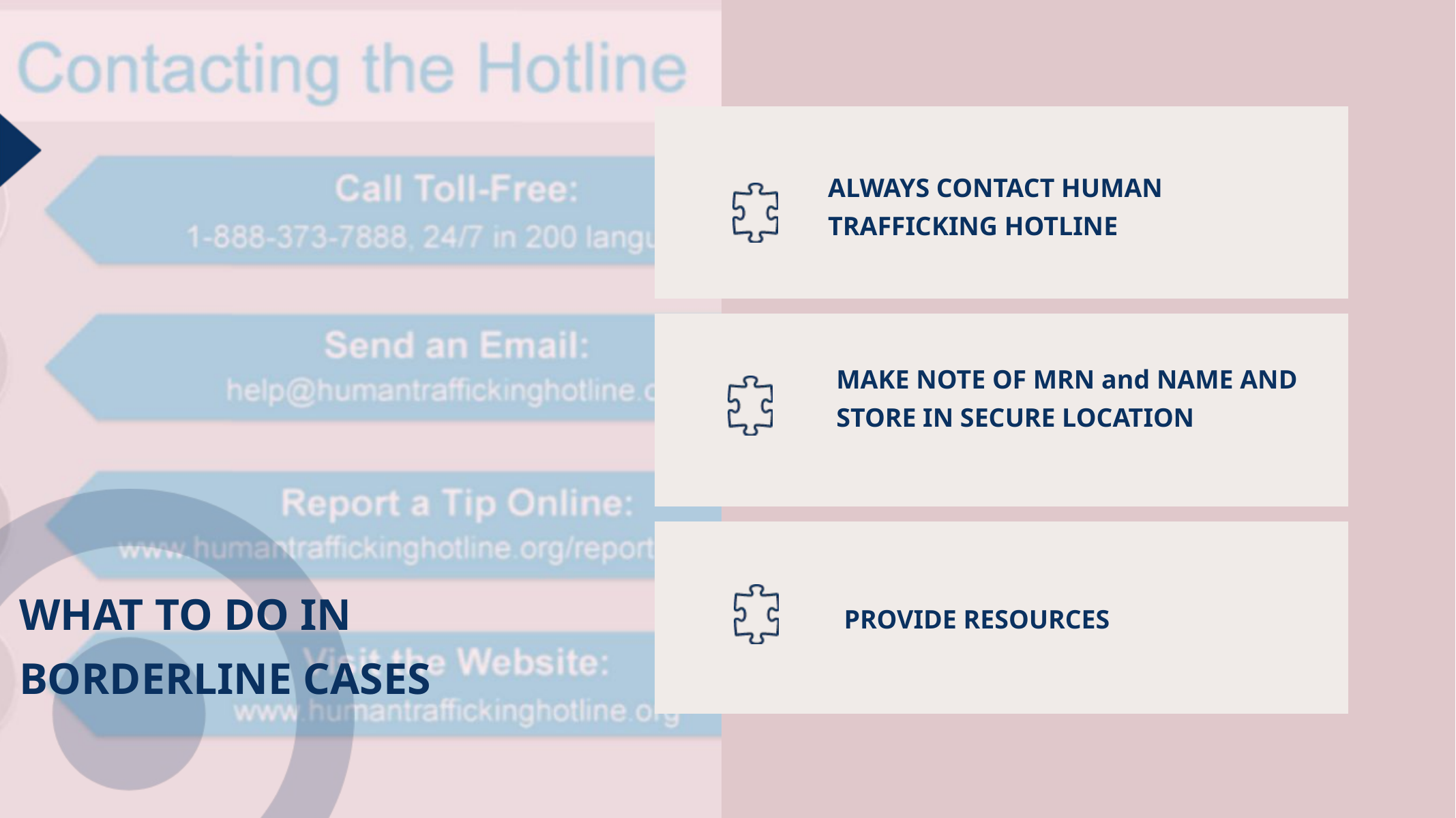

ALWAYS CONTACT HUMAN TRAFFICKING HOTLINE
MAKE NOTE OF MRN and NAME AND STORE IN SECURE LOCATION
WHAT TO DO IN BORDERLINE CASES
PROVIDE RESOURCES

## Slide 45
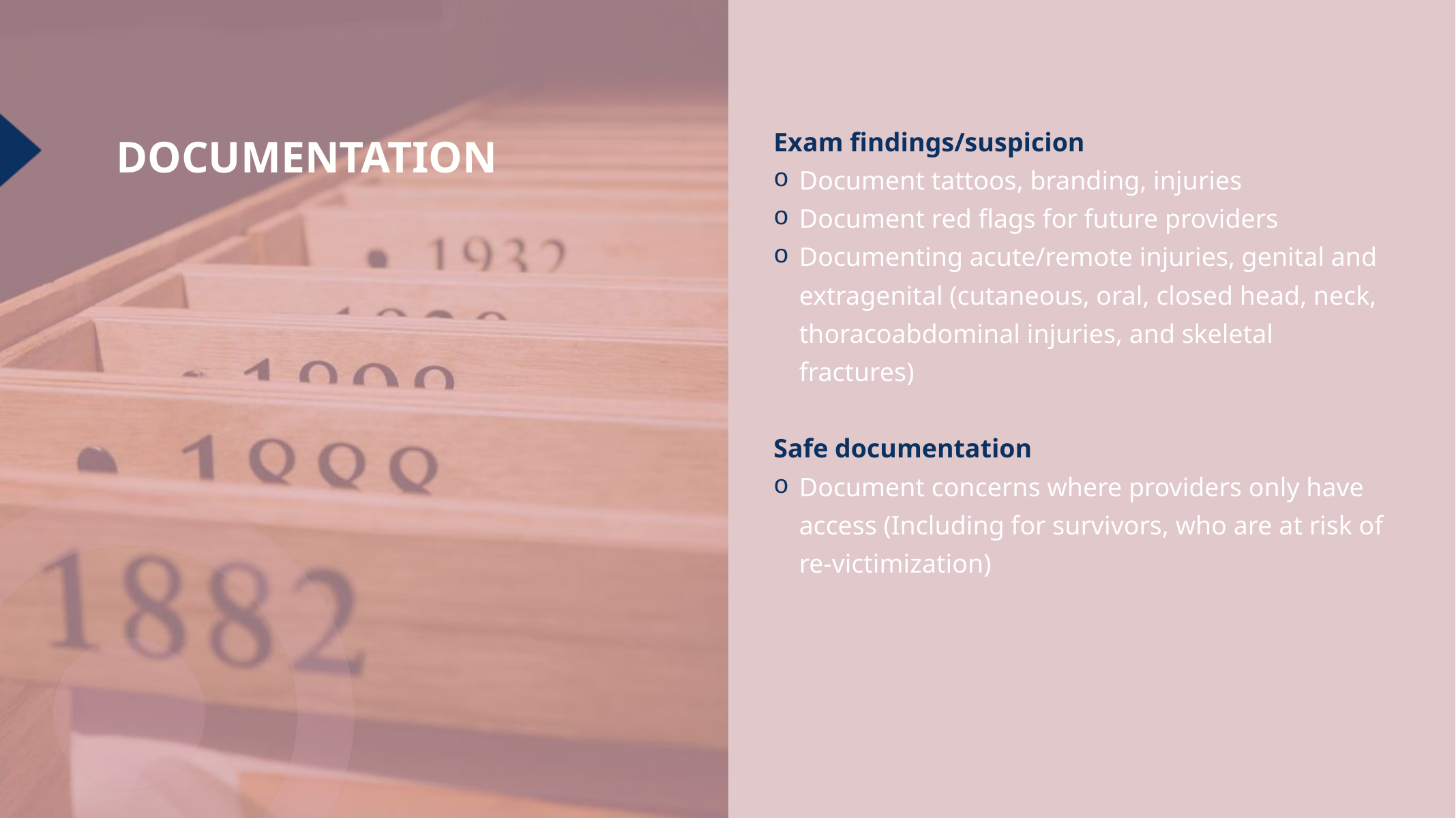

DOCUMENTATION
Exam findings/suspicion
Document tattoos, branding, injuries
Document red flags for future providers
Documenting acute/remote injuries, genital and extragenital (cutaneous, oral, closed head, neck, thoracoabdominal injuries, and skeletal fractures)
Safe documentation
Document concerns where providers only have access (Including for survivors, who are at risk of re-victimization)

## Slide 46
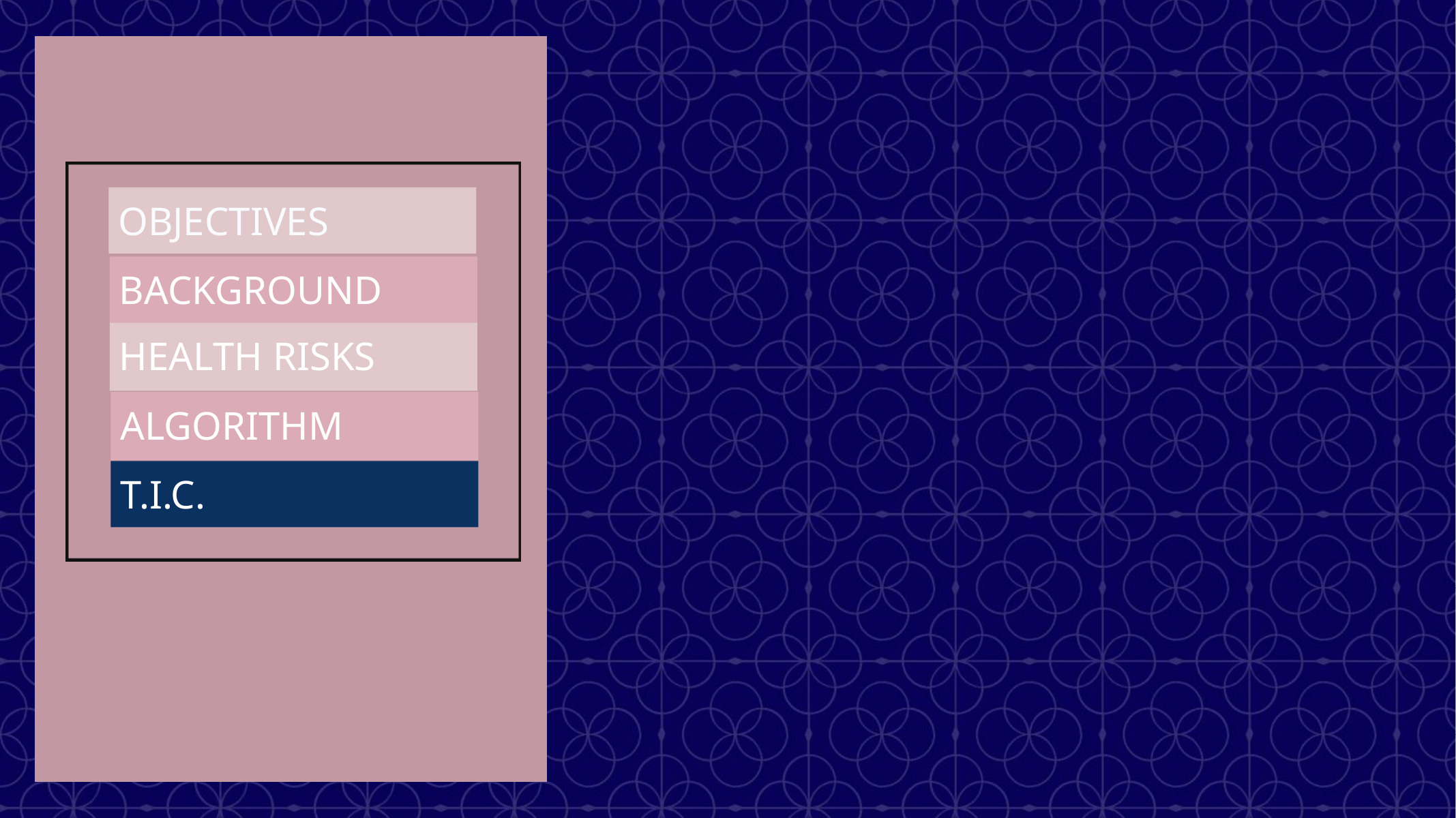

OBJECTIVES
BACKGROUND
HEALTH RISKS
ALGORITHM
T.I.C.

## Slide 47
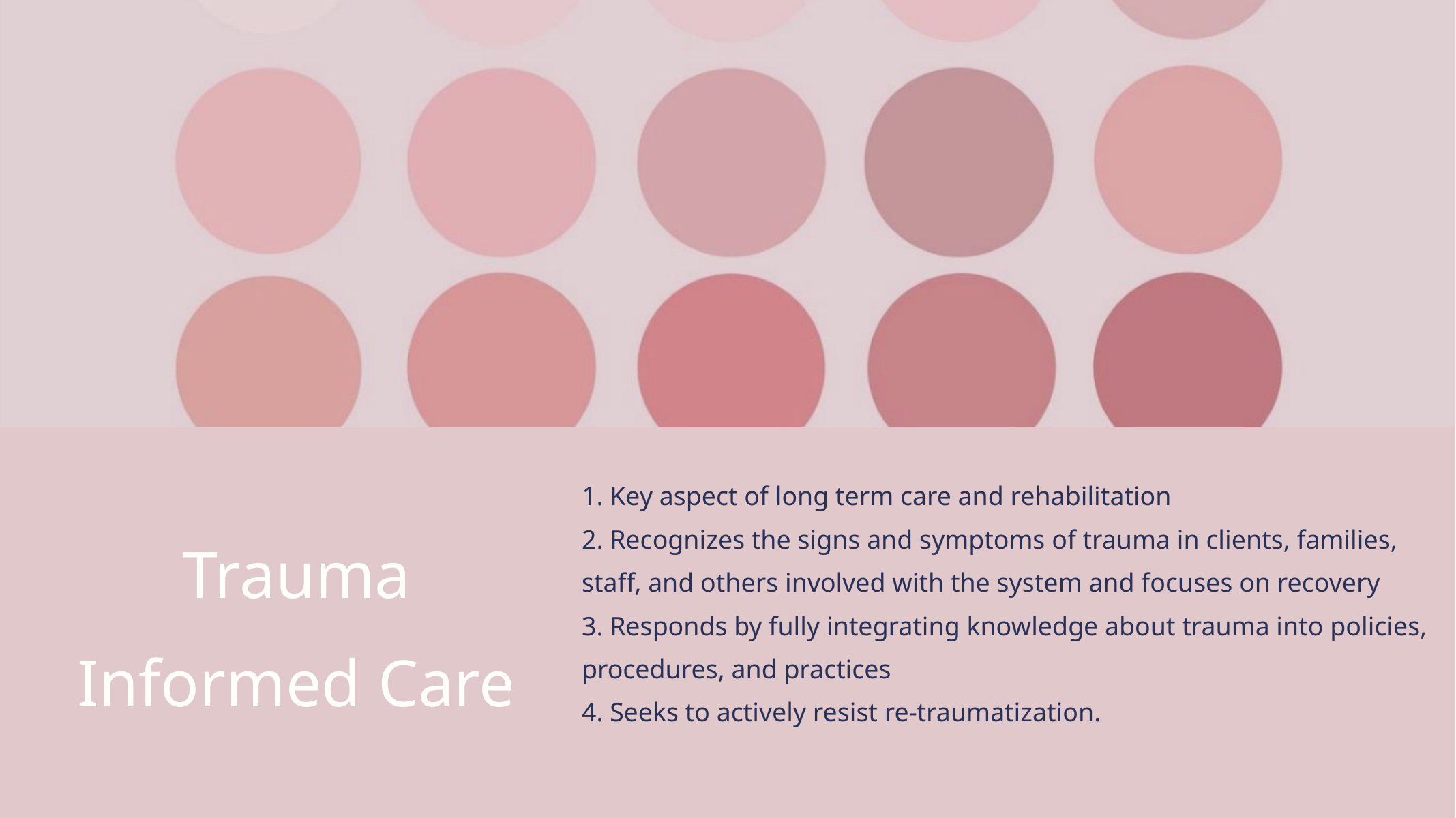

1. Key aspect of long term care and rehabilitation
2. Recognizes the signs and symptoms of trauma in clients, families, staff, and others involved with the system and focuses on recovery
3. Responds by fully integrating knowledge about trauma into policies, procedures, and practices
4. Seeks to actively resist re-traumatization.
Trauma Informed Care

## Slide 48
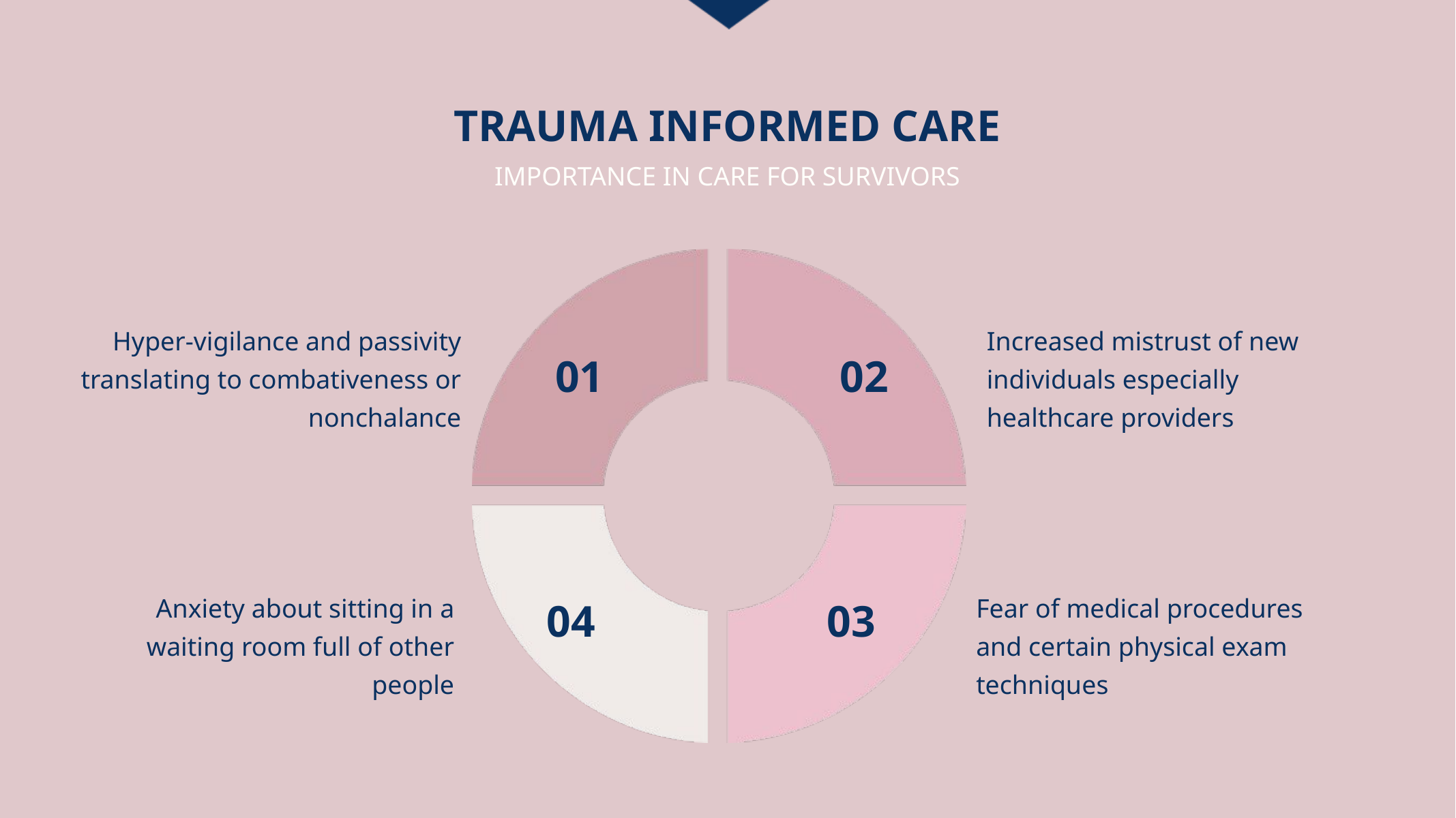

TRAUMA INFORMED CARE
IMPORTANCE IN CARE FOR SURVIVORS
Hyper-vigilance and passivity translating to combativeness or nonchalance
Increased mistrust of new individuals especially healthcare providers
01
02
Anxiety about sitting in a waiting room full of other people
Fear of medical procedures and certain physical exam techniques
04
03

## Slide 49
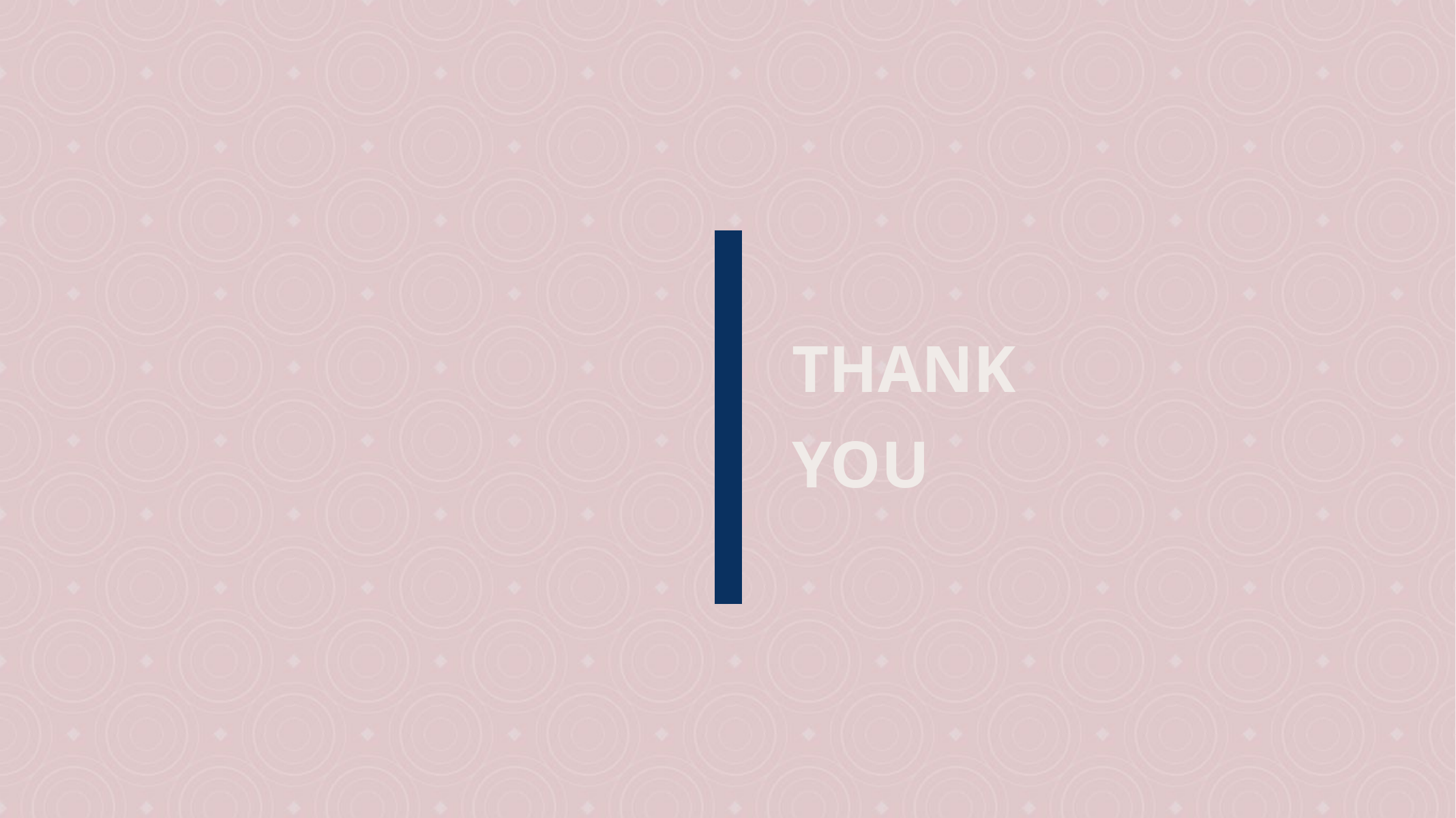

THANK
YOU

## Slide 50
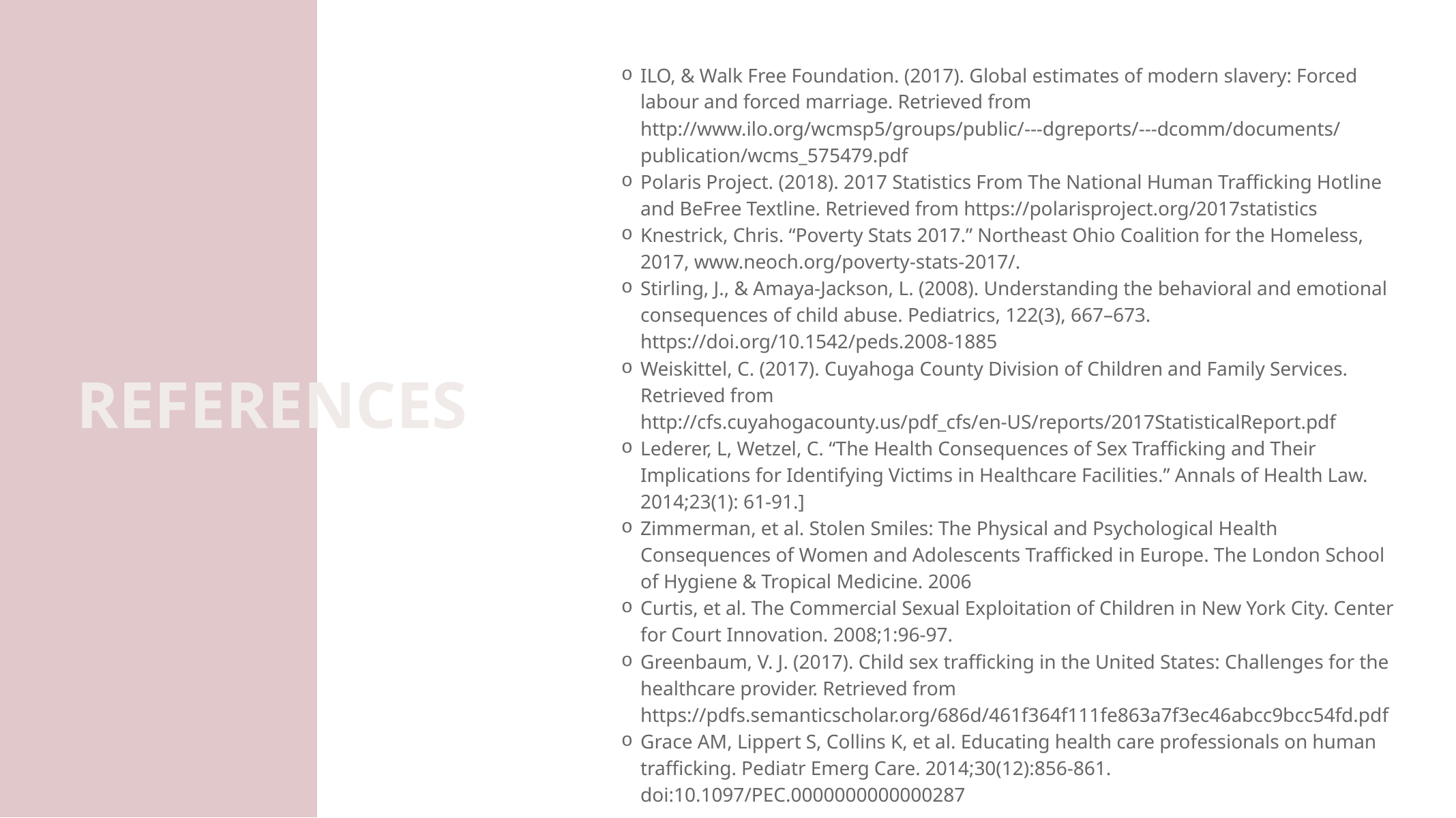

ILO, & Walk Free Foundation. (2017). Global estimates of modern slavery: Forced labour and forced marriage. Retrieved from http://www.ilo.org/wcmsp5/groups/public/---dgreports/---dcomm/documents/publication/wcms_575479.pdf
Polaris Project. (2018). 2017 Statistics From The National Human Trafficking Hotline and BeFree Textline. Retrieved from https://polarisproject.org/2017statistics
Knestrick, Chris. “Poverty Stats 2017.” Northeast Ohio Coalition for the Homeless, 2017, www.neoch.org/poverty-stats-2017/.
Stirling, J., & Amaya-Jackson, L. (2008). Understanding the behavioral and emotional consequences of child abuse. Pediatrics, 122(3), 667–673. https://doi.org/10.1542/peds.2008-1885
Weiskittel, C. (2017). Cuyahoga County Division of Children and Family Services. Retrieved from http://cfs.cuyahogacounty.us/pdf_cfs/en-US/reports/2017StatisticalReport.pdf
Lederer, L, Wetzel, C. “The Health Consequences of Sex Trafficking and Their Implications for Identifying Victims in Healthcare Facilities.” Annals of Health Law. 2014;23(1): 61-91.]
Zimmerman, et al. Stolen Smiles: The Physical and Psychological Health Consequences of Women and Adolescents Trafficked in Europe. The London School of Hygiene & Tropical Medicine. 2006
Curtis, et al. The Commercial Sexual Exploitation of Children in New York City. Center for Court Innovation. 2008;1:96-97.
Greenbaum, V. J. (2017). Child sex trafficking in the United States: Challenges for the healthcare provider. Retrieved from https://pdfs.semanticscholar.org/686d/461f364f111fe863a7f3ec46abcc9bcc54fd.pdf
Grace AM, Lippert S, Collins K, et al. Educating health care professionals on human trafficking. Pediatr Emerg Care. 2014;30(12):856-861. doi:10.1097/PEC.0000000000000287
References
REFERENCES
