# Supplementary material for: A Human Trafficking Educational Program and Point-of-Care Reference Tool for Pediatric Residents
Source: MedEdPORTAL. 2021 Sep 13;17:11179. doi: 10.15766/mep_2374-8265.11179 (PMC8435556; doi:10.15766/mep_2374-8265.11179)
Supplement: Supplementary file 1 — Preceptor Guide.docxPediatric Human Trafficking Presentation.pptxAlgorithm Card Editable.pptxAlgorithm Card.pdfPre- and Postsession Knowledge Assessment.docxKnowledge Assessment with Answers.docx [file mep_2374-8265.11179-s001.zip › C. Algorithm Card Editable.pptx]

## Slide 1
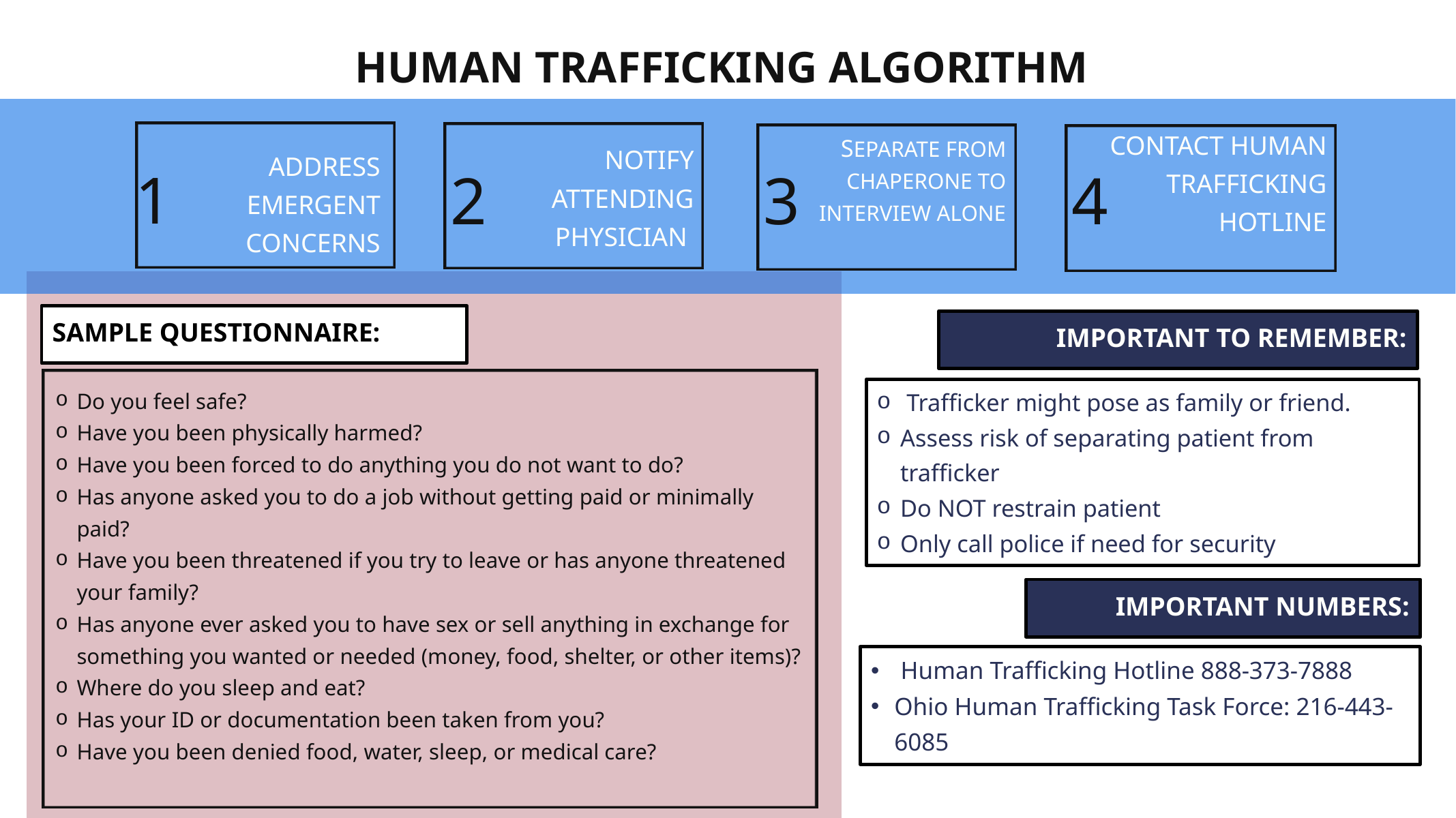

HUMAN TRAFFICKING ALGORITHM
CONTACT HUMAN TRAFFICKING HOTLINE
SEPARATE FROM CHAPERONE TO INTERVIEW ALONE
NOTIFY ATTENDING PHYSICIAN
ADDRESS EMERGENT CONCERNS
1
2
3
4
SAMPLE QUESTIONNAIRE:
IMPORTANT TO REMEMBER:
Do you feel safe?
Have you been physically harmed?
Have you been forced to do anything you do not want to do?
Has anyone asked you to do a job without getting paid or minimally paid?
Have you been threatened if you try to leave or has anyone threatened your family?
Has anyone ever asked you to have sex or sell anything in exchange for something you wanted or needed (money, food, shelter, or other items)?
Where do you sleep and eat?
Has your ID or documentation been taken from you?
Have you been denied food, water, sleep, or medical care?
 Trafficker might pose as family or friend.
Assess risk of separating patient from trafficker
Do NOT restrain patient
Only call police if need for security
IMPORTANT NUMBERS:
 Human Trafficking Hotline 888-373-7888
Ohio Human Trafficking Task Force: 216-443-6085

## Slide 2
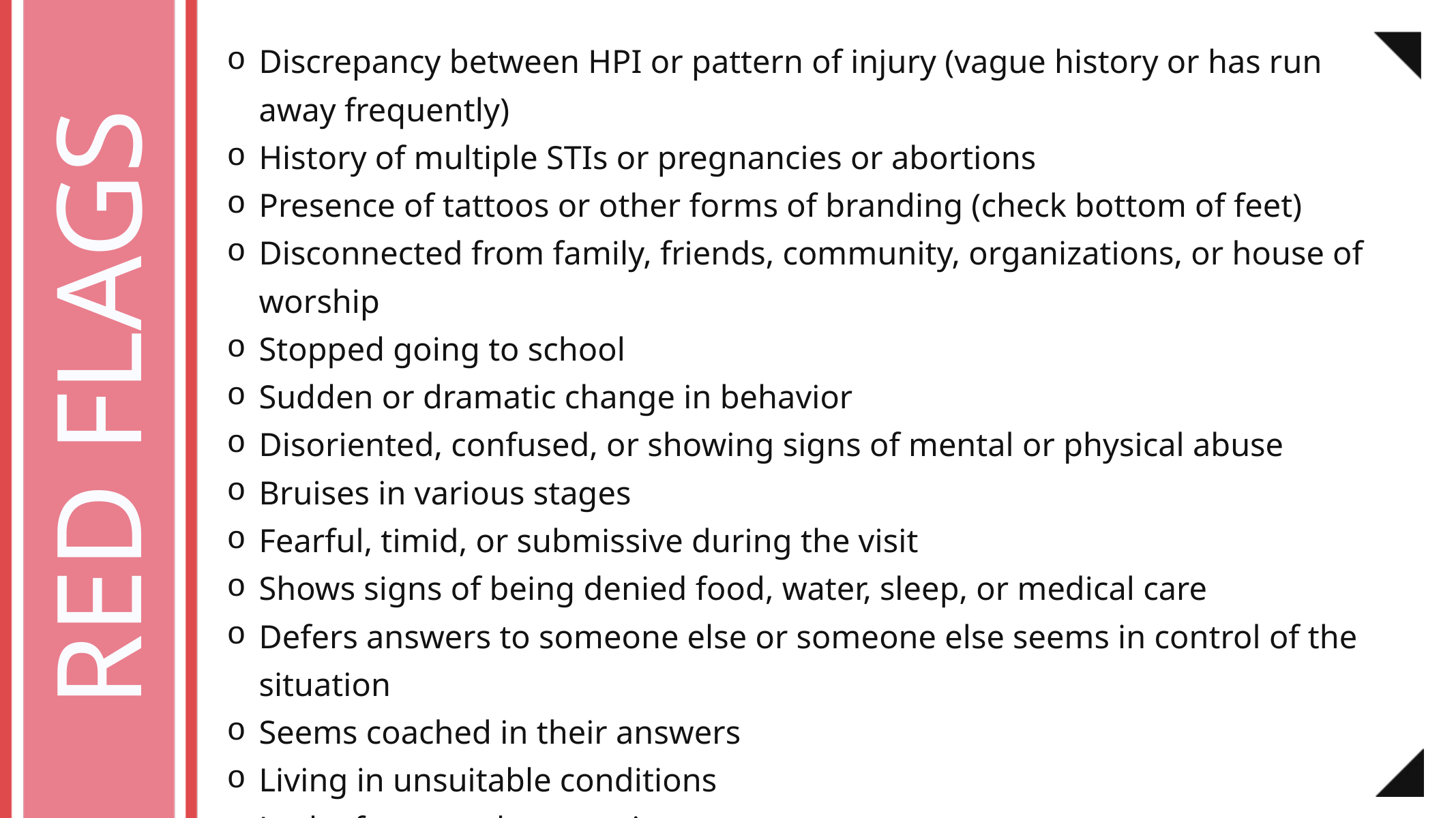

Discrepancy between HPI or pattern of injury (vague history or has run away frequently)
History of multiple STIs or pregnancies or abortions
Presence of tattoos or other forms of branding (check bottom of feet)
Disconnected from family, friends, community, organizations, or house of worship
Stopped going to school
Sudden or dramatic change in behavior
Disoriented, confused, or showing signs of mental or physical abuse
Bruises in various stages
Fearful, timid, or submissive during the visit
Shows signs of being denied food, water, sleep, or medical care
Defers answers to someone else or someone else seems in control of the situation
Seems coached in their answers
Living in unsuitable conditions
Lack of personal possessions
RED FLAGS
