# Supplementary material for: A Human Trafficking Educational Program and Point-of-Care Reference Tool for Pediatric Residents
Source: MedEdPORTAL. 2021 Sep 13;17:11179. doi: 10.15766/mep_2374-8265.11179 (PMC8435556; doi:10.15766/mep_2374-8265.11179)
Supplement: Supplementary file 1 — Preceptor Guide.docxPediatric Human Trafficking Presentation.pptxAlgorithm Card Editable.pptxAlgorithm Card.pdfPre- and Postsession Knowledge Assessment.docxKnowledge Assessment with Answers.docx [file mep_2374-8265.11179-s001.zip › D. Algorithm Card.pdf]

HUMAN TRAFFICKING ALGORITHM

1. ADDRESS EMERGENT CONCERNS
2. NOTIFY ATTENDING PHYSICIAN
3. SEPARATE FROM CHAPERONE TO INTERVIEW ALONE
4. CONTACT HUMAN TRAFFICKING HOTLINE

SAMPLE QUESTIONNAIRE:

- Do you feel safe?
- Have you been physically harmed?
- Have you been forced to do anything you do not want to do?
- Has anyone asked you to do a job without getting paid or minimally paid?
- Have you been threatened if you try to leave or has anyone threatened your family?
- Has anyone ever asked you to have sex or sell anything in exchange for something you wanted or needed (money, food, shelter, or other items)?
- Where do you sleep and eat?
- Has your ID or documentation been taken from you?
- Have you been denied food, water, sleep, or medical care?

IMPORTANT TO REMEMBER:

- Trafficker might pose as family or friend.
- Assess risk of separating patient from trafficker
- Do NOT restrain patient
- Only call police if need for security

IMPORTANT NUMBERS:

- Human Trafficking Hotline 888-373-7888
- Ohio Human Trafficking Task Force: 216-443-6085

RED FLAGS

- Discrepancy between HPI or pattern of injury (vague history or has run away frequently)
- History of multiple STIs or pregnancies or abortions
- Presence of tattoos or other forms of branding (check bottom of feet)
- Disconnected from family, friends, community, organizations, or house of worship
- Has stopped going to school
- Sudden or dramatic change in behavior
- Person disoriented, confused, or showing signs of mental or physical abuse
- Bruises in various stages
- Person fearful, timid, or submissive during the visit
- Person shows signs of being denied food, water, sleep, or medical care
- Person defers answers to someone else or someone else seems in control of the situation
- Person seems coached in their answers
- Living in unsuitable conditions
- Lack of personal possessions
