# Supplementary material for: A Human Trafficking Educational Program and Point-of-Care Reference Tool for Pediatric Residents
Source: MedEdPORTAL. 2021 Sep 13;17:11179. doi: 10.15766/mep_2374-8265.11179 (PMC8435556; doi:10.15766/mep_2374-8265.11179)
Supplement: Supplementary file 1 — Preceptor Guide.docxPediatric Human Trafficking Presentation.pptxAlgorithm Card Editable.pptxAlgorithm Card.pdfPre- and Postsession Knowledge Assessment.docxKnowledge Assessment with Answers.docx [file mep_2374-8265.11179-s001.zip › F. Knowledge Assessment with Answers.docx]

**Human Trafficking Pediatric Educational Survey**

1. Please check your year in Residency Training
   [ ] First year [ ] Second year [ ] Third year [ ] Fourth year
2. A 16 year old girl presents to the ED complaining of abdominal pain and tests positive for chlamydia. During your interview, you find out she is having sex with multiple adult men.
   1. How would you best classify this patient? *(only check one)*[ ] Teen sex worker [ ] Child abuse victim
      [ ] Sex trafficking victim [ ] Promiscuous teenager
      [ ] Street youth/Runaway [ ] I don’t know

You notify the police and they tell you she is on the missing persons list. When she finds out you contacted the authorities she becomes angry and states it is her choice to live this way and does not want her parents to find out.

- 1. Given this information, would you change how you classified this patient?
     [ ] yes [ ] no
     1. If yes, how would you best classify this patient? *(only check one)*
        [ ] Teen sex worker [ ] Child abuse victim
        [ ] Sex trafficking victim [ ] Promiscuous teenager
        [ ] Street youth/Runaway [ ] I don’t know

1. A 17 year old homeless boy presents for medical treatment. The boy has a history of running away, is suspected for gang involvement, and has been charged with a number of misdemeanors. He has a history of sexual abuse. On exam he has areas of physical bruising. He also tests positive for gonorrhea.
   1. How would you best classify this patient?
      [ ] Street youth/Runaway [ ] Potential teen sex worker
      [ ] Potential human trafficking victim [ ] Illegal immigrant
      [ ] I don’t know

      What is the next best step in caring for this patient after treating immediate medical needs?
      [ ] Counsel on risks of gang involvement and unsafe sex and discharge

[ ] Discharge as patient is at age of legal consent and is able to make his own decisions
[ ] Assess patient’s immediate safety and notify the National Human Trafficking Hotline about your concerns
[ ] Consult social work to provide resources for homeless youth shelters and support
[ ] I don’t know

1. True or False
   1. The travel, transfer or movement across state or national borders differentiates human trafficking from the commercial sexual exploitation of children *(false)*
   2. The majority of child sex trafficking victims in America are U.S. citizens *(true)*
   3. Greater than 90 percent of child trafficking victims are female *(false)*
   4. It is very likely a physician will encounter a human trafficking victim in the ED *(true)*
   5. It is unlikely a physician will encounter a human trafficking victim in the primary care setting *(false)*
   6. Sexual health issues are the most common short- and long-term health consequences of human trafficking *(false)*
   7. If a teenager consents to trading sex for something of monetary value, this cannot be considered sex trafficking *(false)*
2. Which of the following are considered risk factors for of becoming a trafficking victim? (*select all that apply)*
   1. Physical or sexual abuse
   2. Interaction with the foster care system
   3. Family history of mental illness
   4. Reliance on public benefits
   5. Running away from home
3. Have you had any training on human trafficking in the past?
   *This may include formal in-person training, modules, lectures or extensive personal reading on the subject.*
    [ ] yes [ ] no
   1. If yes, did this training include information specific to the trafficking of children?
      [ ] yes [ ] no
4. How comfortable do you feel:
   1. Defining human trafficking
      [ ] Very uncomfortable [ ] Uncomfortable [ ] Neutral [ ] Comfortable [ ] Very Comfortable
   2. Recognizing the signs of human trafficking in one of your patients
      [ ] Very uncomfortable [ ] Uncomfortable [ ] Neutral [ ] Comfortable [ ] Very Comfortable
   3. Knowing who to call if you think your patient is a victim of human trafficking
      [ ] Very uncomfortable [ ] Uncomfortable [ ] Neutral [ ] Comfortable [ ] Very Comfortable
   4. Describing the health consequences of human trafficking
      [ ] Very uncomfortable [ ] Uncomfortable [ ] Neutral [ ] Comfortable [ ] Very Comfortable
   5. How likely do you think you are to come across a human trafficking victim as a pediatric resident?

[ ] Very unlikely [ ] Unlikely [ ] Neutral [ ] Likely [ ] Very Likely

If you have in any way been triggered or made uncomfortable by these survey questions, we urge you to contact the following resources:

National Suicide Prevention Lifeline: 1-800-273-8255,<https://suicidepreventionlifeline.org/>

Crisis Text Line: Text HOME to 741741,<https://www.crisistextline.org/>

National Human Trafficking Hotline: 1-888-373-7888,<https://humantraffickinghotline.org/>
